# The Distribution and Dispersal of Large Haploblocks in a Superspecies

**DOI:** 10.1111/mec.17731

**Published:** 2025-03-17

**Authors:** Darren Irwin, Staffan Bensch, Caleigh Charlebois, Gabriel David, Armando Geraldes, Sandeep Kumar Gupta, Bettina Harr, Paul Holt, Jessica H. Irwin, Vladimir V. Ivanitskii, Irina M. Marova, Yongchao Niu, Sampath Seneviratne, Ashutosh Singh, Yongjie Wu, Shangmingyu Zhang, Trevor D. Price

**Affiliations:** ^1^ Department of Zoology and Biodiversity Research Centre University of British Columbia Vancouver British Columbia Canada; ^2^ Department of Biology Lund University Lund Sweden; ^3^ Key Laboratory of Zoological Systematics and Evolution, Institute of Zoology Chinese Academy of Sciences Beijing China; ^4^ University of Chinese Academy of Sciences Beijing China; ^5^ Wildlife Institute of India Dehradun India; ^6^ Max‐Planck‐Institut für Evolutionsbiologie Germany; ^7^ Tongzhou district, Beijing China; ^8^ Department of Biology Lomonosov Moscow State University Moscow Russia; ^9^ Biozeron Shenzhen, Inc. Shenzhen China; ^10^ Department of Zoology & Environment Sciences, Faculty of Science University of Colombo Colombo Sri Lanka; ^11^ Salim Ali Centre for Ornithology and Natural History Coimbatore India; ^12^ Key Laboratory of Bioresources and Ecoenvironment (Ministry of Education), College of Life Sciences Sichuan University Chengdu Sichuan China; ^13^ Department of Ecology and Evolution The University of Chicago Chicago Illinois USA

**Keywords:** hybridization, *Phylloscopus plumbeitarsus*, *Phylloscopus trochiloides*, reproductive isolation, ring species, speciation

## Abstract

Haploblocks are regions of the genome that coalesce to an ancestor as a single unit. Differentiated haplotypes in these regions can result from the accumulation of mutational differences in low‐recombination chromosomal regions, especially when selective sweeps occur within geographically structured populations. We introduce a method to identify large well‐differentiated haploblock regions (LHBRs), based on the variance in standardised heterozygosity (ViSHet) of single nucleotide polymorphism (SNP) genotypes among individuals, calculated across a genomic region (500 SNPs in our case). We apply this method to the greenish warbler ( *Phylloscopus trochiloides* ) ring species, using a newly assembled reference genome and genotypes at more than 1 million SNPs among 257 individuals. Most chromosomes carry a single distinctive LHBR, containing 4–6 distinct haplotypes that are associated with geography, enabling detection of hybridisation events and transition zones between differentiated populations. LHBRs have exceptionally low within‐haplotype nucleotide variation and moderately low between‐haplotype nucleotide distance, suggesting their establishment through recurrent selective sweeps at varying geographic scales. Meiotic drive is potentially a powerful mechanism of producing such selective sweeps, and the LHBRs are likely to often represent centromeric regions where recombination is restricted. Links between populations enable introgression of favoured haplotypes and we identify one haploblock showing a highly discordant distribution compared to most of the genome, being present in two distantly separated geographic regions that are at similar latitudes in both east and central Asia. Our results set the stage for detailed studies of haploblocks, including their genomic location, gene content and contribution to reproductive isolation.

## 1 Introduction

Every site in the genome coalesces to a common ancestor, but because of recombination, different sites often coalesce to different ancestors, which may be far apart in both space and time. In the absence of recombination, however, physically linked sites share the same history of genealogical coalescence to the same ancestors. Genomic regions that show such shared ancestral genealogy are termed haplotype blocks, or more concisely haploblocks, and considerable work is being invested in understanding their size, detection, origin and maintenance (Shipilina et al. 2023). Some haploblocks are large and contain just a few segregating haplotypes that are well differentiated from each other, implying long coalescent times. Classic examples include chromosomal inversions, which can arise through mutation and then result in suppressed recombination between the inverted and original versions. These can rise to intermediate frequencies and be maintained by forms of balancing selection, and may persist for millions of years, even persisting across species boundaries (Hager et al. 2022; Todesco et al. 2020). If gene flow moves combinations of alleles that are favoured in one population into another where they are disfavoured, then a local inversion that captures locally adapted alleles can be favoured (Hooper and Price 2017; Kirkpatrick and Barton 2006). The noninverted version of the region, which does not recombine with the original version, subsequently increases in frequency and accumulates genetic differences. Alternatively, haploblocks may arise in allopatry through selection and/or drift, and in such cases large blocks are expected in regions with inherently low recombination (Shipilina et al. 2023). Introgression can then bring divergent haploblocks together in the same population. In this case, haploblocks should be especially common in chromosomal regions with inherently low recombination (N. Wang et al. 2023; Z. Wang et al. 2022).

Once haploblocks have arisen for any reason, they may be transferred between populations. Given that a complete loss of hybrid fitness generally takes millions of years (Coyne and Orr 2004; T. D. Price and Bouvier 2002; Weir and Price 2011), opportunities for genetic exchange include not only occasional dispersal events but also changes in range that bring populations in and out of contact. Introgression of haploblocks between populations may be limited by selection; for example, if hybrids have low fitness due to previously untested alleles being brought together, they may also simply break down through recombination in the recipient population. However, haploblocks may persist intact if they carry sets of alleles that are favoured in the recipient population. We now know that genetic exchange between divergent taxa is common, with most examples concerning the transfer of single genes (Aguillon et al. 2022; Edelman and Mallet 2021; Taylor and Larson 2019). The accumulation of many genetic differences between well‐differentiated haploblocks makes it seem likely that they have much potential to influence the speciation process in both negative and positive ways.

In this paper, we place the origin, maintenance and spread of large haploblocks in a geographical context. Across space, regions of the genome that are well differentiated (i.e., those with high *F*_ST_; which is the proportion of total population variation that is explained by between‐population differences) often show low absolute differences between populations (i.e., low *D*_xy_) (Cruickshank and Hahn 2014; Irwin et al. 2016, 2018). One prominent explanation for this finding is recurrent selective sweeps, some of which occurred early and crossed between populations, thereby reducing *D*_xy_, and some of which occurred later, reducing variation within populations, thereby increasing *F*_ST_ (this is the ‘sweep‐before‐differentiation’ model of Irwin et al. 2016, 2018). Haploblocks are well suited to have arisen in this manner, given the large number of linked sites that are potentially subject to adaptive mutations. If this is the main mechanism of haploblock origin, we expect absolute differentiation to be lower in haploblocks than in the rest of the genome. That prediction contrasts with the expectation if an inversion captured differentially adapted alleles and protected them from gene flow and recombination while the rest of the genome continued to be exchanged between populations. In that case, we would expect absolute differentiation to be higher in haploblocks than in the rest of the genome (Lundberg et al. 2017). We refer to these two hypotheses regarding haploblock origin and development as the ‘sweep‐before‐differentiation’ and ‘inversion’ hypotheses.

A thorough analysis of the origin and role of large well‐differentiated haploblocks in speciation will require close inspection of the functions of each one. However, tests for the geographical origin and subsequent introgression across taxa can utilise superspecies—defined as a monophyletic group of geographically structured but ecologically similar forms (Mayr and Diamond 2001)—to examine the haploblock distribution and connections between populations. This is an especially promising approach when degrees of introgression vary between different taxa in the superspecies complex because reproductive isolation, geographical separation, or dispersal routes differ among taxa in ways that promote or restrict interbreeding, and hence gene transfer.

**FIGURE 1:**
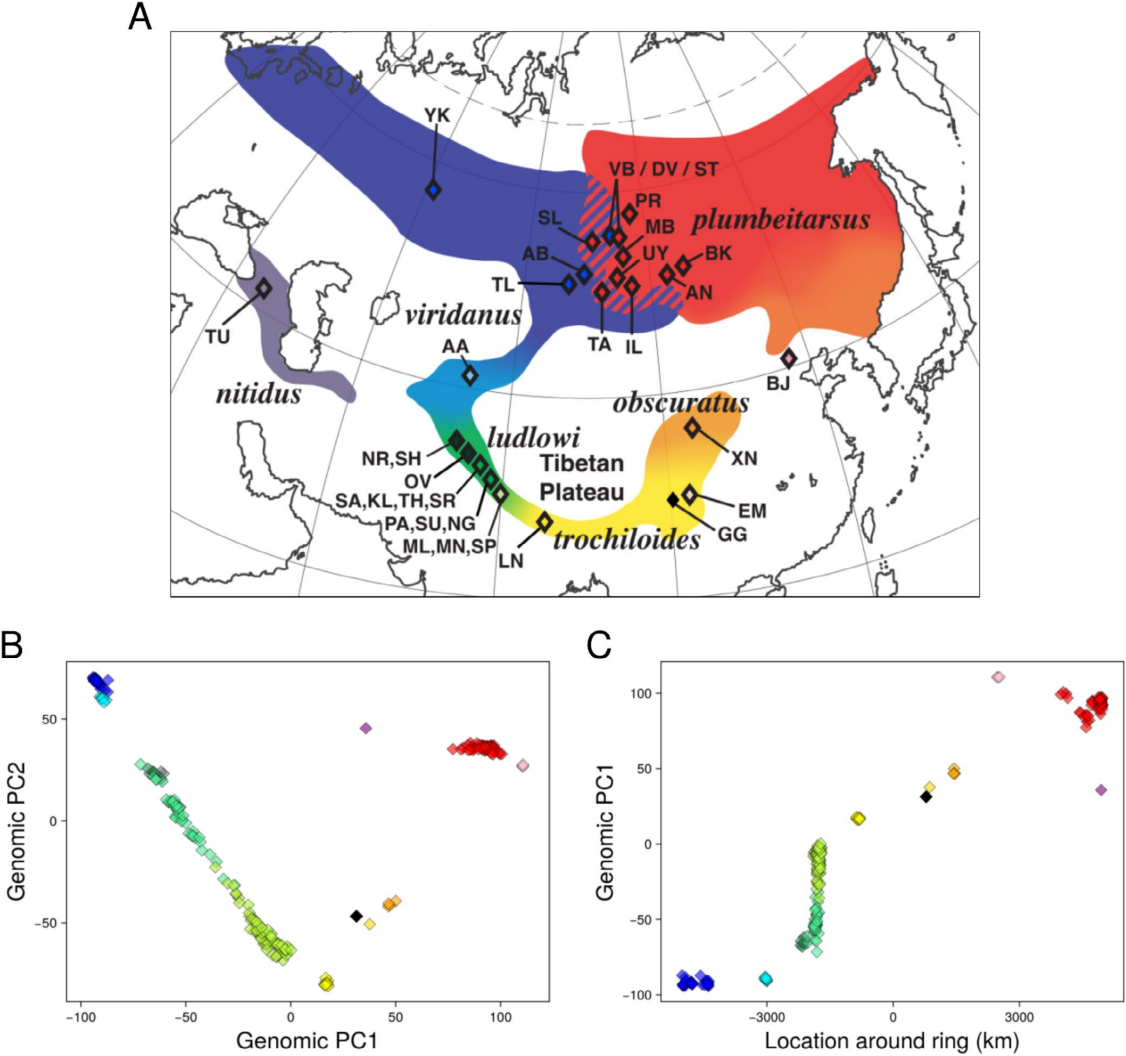
Greenish warblers show strong geographic structure, with west Siberian (*P. t. viridanus*) and east Siberian (*P. t. plumbeitarsus*) populations showing the most differentiation and populations to the south show stepwise progression in genetic signatures through subspecies *ludlowi*, *trochiloides* and *obscuratus*. (A) Map of sampling locations and subspecies ranges, (B) scatter plot of the first two principal components of genomic variation (PC1 captures 12.1% of the total variance, and PC2 6.4%), with each diamond representing one individual coloured according to the phenotype and map, and the black diamond showing the PCA coordinates of the reference genome. (C) The association of PC1 with location around the ring (measured from west Siberia down and around the ring to east Siberia). Note the western taxon *nitidus* is represented by two individuals in panel B (the two grey symbols near the upper left cluster of green symbols) but is not included in panel C because *nitidus* is outside of the main ring.

Here we study the range‐wide distribution of large haploblocks in the greenish warbler ( *Phylloscopus trochiloides* ) superspecies across Asia, focusing on contact zones. The greenish warbler forms a classic ring species, defined as a ring of connected populations broken in one place by a species boundary (Mayr 1942, 1970; Cain 1954; Wake and Yanev 1986; Martens and Päckert 2007; Irwin and Wake 2016; Kuchta and Wake 2016; Pruett 2016, see Figure 1A). In this superspecies, two parapatric forms have achieved species status (i.e., near complete reproductive isolation), yet other locations of contact between neighbouring populations have resulted in transition zones (Alcaide et al. 2014; Irwin, Bensch, et al. 2001; Mayr 1942; Ticehurst 1938). All recognised ring species, including the greenish warbler, show evidence of periods of geographic division followed by secondary contact (Irwin, Bensch, et al. 2001; Irwin, Irwin, et al.^2001^; Kuchta and Wake 2016; Mayr 1970; Pruett 2016) as expected given climate fluctuations over the last 2 million years (Hewitt 1996, 2011). Genetic contact between populations might also be expected to occur because greenish warblers migrate to and from their winter quarters in southern Asia, resulting in opportunities for displaced individuals to occasionally enter different regions.

We first develop a method to identify large well‐differentiated haplotype block regions (LHBRs). While most approaches for identifying haploblocks rely on phased genomes (usually arrived at through a combination of long‐read sequencing and bioinformatic inference; Shipilina et al. 2023), our method is based on the quantification of a key characteristic: an individual with two copies of a certain haploblock type will have low average heterozygosity across that region, whereas individuals with two different types of the haploblock will have high average heterozygosity across that region. Hence, a region containing large haploblocks will have high variation in heterozygosity among individuals compared to most other parts of the genome, after controlling for variation in the average heterozygosity in each region. We calculate variance in standardised heterozygosity (ViSHet) in windows across the genome and set a cut‐off value to identify large haploblock regions. We then use Principal Components Analysis (PCA) on SNP genotypes to determine the number of distinct haploblock types for each LHBR. We use the LHBR genotypes to characterise geographic differentiation around the greenish warbler ring of populations and to assess the degree of interbreeding across the complex. We also weigh the evidence for both the sweep‐before‐differentiation and inversion hypotheses for haploblock development.

## 2 Materials and Methods

### 2.1 Study System

The greenish warblers (members of the *Phylloscopus trochiloides* complex) have featured prominently in the literature on speciation (Alcaide et al. 2014; Coyne and Orr 2004; Irwin, Bensch, et al. 2001; Martins et al. 2013; Mayr 1942, 1970; T. Price 2008; Ticehurst 1938). Ticehurst (1938) studied morphological variation in greenish warblers and concluded there were two distinct morphologically differentiated populations in central Siberia without local intermediates, yet these types were connected by a long chain of intergrading forms encircling the Tibetan Plateau to the south. Ticehurst grouped greenish warblers in this ring under five subspecies names (west Siberian *viridanus*, central Asian *ludlowi*, Himalayan *trochiloides*, central Chinese *obscuratus* and east Siberian *plumbeitarsus*). Subsequent analyses have supported Ticehurst's description of *viridanus* and *plumbeitarsus* as showing distinct differences while being connected by a gradient of intermediate characteristics through the south (Alcaide et al. 2014; Irwin, Bensch, et al. 2001; Irwin et al. 2005; Scordato 2018) (Figure 1A). Deep divisions in mitochondrial DNA around the ring occur in the north between *viridanus* and *plumbeitarsus*, and in the southwest within *ludlowi*. Genomic analyses have demonstrated highly restricted gene flow across the boundary in the north (hence *viridanus* and *plumbeitarsus* are considered biological species) and a narrow genomic transition zone in the southwest (Alcaide et al. 2014). A sixth named subspecies, *nitidus*, is outside of the main ring to the west. This is often considered a distinct species, the green warbler *Phylloscopus nitidus* , although it is phenotypically and genetically more similar to *viridanus* than *viridanus* is to *obscuratus* and *plumbeitarsus*.

### 2.2 Sampling

For the present study, we generated a new high‐quality greenish warbler reference genome and surveyed variation at more than 1 million genetic loci from 257 individuals. In addition to the 133 samples reported in Alcaide et al. (2014), we obtained 124 new samples: 59 from the Siberian contact zone between *viridanus* and *plumbeitarsus*; 62 from the region of steep genetic change within the southwestern part of the ring (i.e., within *ludlowi* and western *trochiloides*); and 3 from a newly discovered breeding location of greenish warblers on Dongling Mountain, Beijing, China (ebird.org), within the gap between *obscuratus* and *plumbeitarsus* on the eastern side of the ring (Figure 1A). Here we analyse genomic data from these 124 samples together with the 133 more broadly distributed samples (Table S1) that were previously included in the genomic differentiation analyses of Alcaide et al. (2014) and Irwin et al. (2016).

### 2.3 Reference Genome Assembly

We produced a high‐quality whole‐chromosome greenish warbler reference genome using a male individual collected at Gongga Mountain, Sichuan Province, China (29.5°N, 102.0°E), in June 2021. Assembly was conducted by Biozeron Shenzhen Inc. in Shenzhen, China, based on two types of sequences. First, two cells of PacBio HiFi circular consensus long reads (Wenger et al. 2019) were produced, resulting in a total of 49.18 Gb of sequence, with an average read length of 15,935 bp. These reads were constructed into draft phased contigs using FALCON and FALCON‐Unzip in the pb‐assembly tool suite version 0.0.8 (https://github.com/PacificBiosciences/pb‐assembly). Second, a Hi‐C proximity ligation library was prepared and sequenced using Illumina technology (Lieberman‐Aiden et al. 2009), resulting in 342,732,099 paired reads for inferring the proximity of different DNA sequences in the genome. Hic‐Pro version 2.11.1 (Servant et al. 2015) was used to filter and map these reads onto the draft contigs from the PacBio assembly. Then, Juicer version 1.6.2 (Durand et al. 2016a), 3D‐DNA version 180,114 (Dudchenko et al. 2017) and Juicebox version 1.11.8 (Durand et al. 2016b) were used to cluster contigs into chromosomes, validate contig orientation and remove ambiguous fragments.

We used D‐genies (Cabanettes and Klopp 2018) to infer large regions of homology between our greenish warbler genome scaffolds and the zebra finch *Taeniopygia guttata* genome (version 3.2.4, Genbank sample ABQF01000000, NCBI RefSeq assembly GCF_000151805; Warren et al. 2010) and we named greenish warbler scaffolds based on this homology. All chromosomes in the zebra finch assembly had a clear counterpart in the greenish warbler and are named accordingly (see Supporting Information for details).

To annotate the reference genome, we first ran RepeatModeler2 (Flynn et al. 2020) to detect repetitive element families present in the greenish warbler genome and create a library of consensus sequences representing each one. Then we used RepeatMasker (Smit et al. 2013), in conjunction with this consensus library, to annotate and soft‐mask repetitive regions in the reference assembly. Finally, we applied the BRAKER3 pipeline (Gabriel et al. 2024) to the masked reference assembly to estimate the locations of genes. Intrinsic evidence for gene annotation was provided to BRAKER3 in the form of an RNA‐seq library prepared from the same individual sequenced for the reference assembly. To prepare the genome for Genbank submission, we used NCBI FCS (Astashyn et al. 2024) to remove adaptors and contaminants from the sequence and GAG (Geib et al. 2018) to make matching alterations to the annotation file.

### 2.4 Genotyping

We conducted genotyping‐by‐sequencing (GBS; Elshire et al. 2011) of the 124 new samples according to the protocol of Alcaide et al. (2014). Two GBS libraries were prepared using these new samples. Sequences produced from all four GBS libraries (two produced new for this study; two from Alcaide et al. 2014) were demultiplexed using a custom script and trimmed using TRIMMOMATIC‐0.32 (Bolger et al. 2014; for details, see Irwin et al. 2018). We mapped all reads to our new greenish warbler reference genome using BWA‐MEM 0.7.17 (Li and Durbin 2009) on default settings. The programs Picard‐tools 1.97 (https://broadinstitute.github.io/picard/) and Samtools (Li et al. 2009) were then used to produce a BAM file for each individual containing the alignment of GBS reads to the reference genome. We used GATK 3.8 (McKenna et al. 2010) to call genotypes (with the HaplotypeCaller command) and combine genotypes from all individuals (using the GenotypeGVCFs command) into a single VCF (Variant Call Format) file.

We applied a series of filters to ensure that our analysis would be based on highly reliable genotypes. Indels, SNPs with more than 2 alleles, and SNPs with missing genotypes in more than 60% of individuals were removed using vcftools 0.1.12b (Danecek et al. 2011). SNPs with MQ (Mapping Quality) below 20 or with heterozygosity above 60% were removed using scripts provided by Owens et al. (2016).

To conduct all subsequent data analyses and visualisations, we developed a new package of functions, GenomicDiversity.jl (https://github.com/darreni/GenomicDiversity.jl), and a set of scripts in the Julia programming language (Bezanson et al. 2017). The data matrix imported into Julia had 2,431,709 SNPs (i.e., columns) from 305 individual greenish warbler GBS runs (i.e., rows; these include some multiple runs). In Julia, we conducted an additional series of filters to ensure the quality of the downstream analysis. We removed 11 duplicate runs and 33 individuals that were missing genotypes at more than 40% of the 2,431,709 SNPs. We then filtered out SNPs that were missing genotypes in more than 5% of these 261 individuals. After that, we removed individuals missing genotypes at more than 10% of the remaining SNPs. For the Z chromosome, we developed a novel filtering procedure to ensure that the Z chromosome PCA was not affected by W chromosome homologues (see Supporting Information for details). Finally, we included only those SNPs that occur on one of the major chromosome scaffolds (these are 1–15, 17–28, 1A, 4A and Z). These filtering steps resulted in a final data matrix of 257 greenish warbler individuals at 1,003,924 SNPs.

### 2.5 Identification of Large Haploblock Regions (LHBRs)

We define large haploblock regions (LHBRs) as being parts of the genome where individual genotypes show high association over a long sequence of a chromosome and where there are distinctly recognisable haplotypes over that long sequence. In such regions, most individuals can be clearly recognised as being either homozygous for a particular haplotype or heterozygous for two haplotypes. Recombination between haplotypes might complicate this inference for a few individuals, but in the clearest LHBRs, the great majority of individuals can be unambiguously assigned to homozygous or heterozygous haplotype groups. This contrasts with non‐LHBR parts of the genome, in which individuals cannot be clearly assigned to a single genotype description for a long sequence of a chromosome.

This definition of LHBRs leads to our automated approach for identifying them in the genome: First, we divide the genome into contiguous windows of 500 SNPs each. For each window, we calculate the mean heterozygosity (across all 500 SNPs) for each individual, and then divide those values by the mean of all individuals. This results in standardised heterozygosity for each individual and window. We then calculate, for each window, the variance in standardised heterozygosity (ViSHet). High values of this ViSHet statistic across the genome clearly identify regions that were also noticed as having strong haploblock structure during visual inspection of the dataset. We determined that a ViSHet value of 0.4 provided an appropriate threshold value to use to distinguish LHBRs for this study. This threshold results in 5.8% of the genome being in LHBRs. Contiguous windows with ViSHet values above 0.4 were treated as part of a single LHBR.

### 2.6 Determination of Haploblock Genotypes

At many LHBRs, individual standardised heterozygosity values fell into two clusters: low values corresponding to individuals essentially homozygous for that LHBR, and high values corresponding to heterozygotes for that LHBR. For each LHBR, we determined an appropriate threshold value of individual standardised heterozygosity to distinguish these categories, which, together with Principal Components Analysis (see below) enabled LHBR genotyping of individuals. We closely examined variation at the largest LHBR on each chromosome; LHBR genotypes could be clearly determined for the largest LHBR on 12 chromosomes (see Results).

### 2.7 Analyses of Genetic Relationships Using PCA

To visualise genomic relatedness among individuals, we used Principal Components Analysis (PCA) using the Singular Value Decomposition (SVD) method, as implemented in the MultivariateStats.jl package (https://juliastats.org/MultivariateStats.jl/dev/). For PCA based on SNP variation from the whole genome, missing genotypes were imputed using the K Nearest Neighbours (KNN) algorithm with *K* = 1 and a Euclidean distance metric, as implemented in the Impute.jl package (https://invenia.github.io/Impute.jl/latest/). This imputation was done for each scaffold separately, such that imputation would be influenced only by SNP variation on the same scaffold. For PCA based on a specific genomic region, missing genotypes were imputed using the SVD algorithm (Troyanskaya et al. 2001), also using the Impute.jl package. We generated plots of PC1 vs. PC2, with each individual represented by a symbol coloured according to subspecies/location (see Figure 1A). For some PCA plots, we added the position of the reference genome by applying the PC loadings to a vector containing entirely homozygous reference genotypes at the SNP locations.

For specific LHBRs, we conducted PCA on all individuals and examined PCA plots in two ways: first with all individuals, and then with just the individuals categorised as homozygous for that LHBR. This approach facilitated the inference of the number of distinct homozygous clusters (i.e., haploblock types) for that LHBR, as well as the inference of PCA clusters that correspond to heterozygotes between two haplotypes. The principal component (PC) values corresponding to these clusters were used to determine individual LHBR genotypes.

We generated a measure of location around the ring for the purpose of graphing how genomic PC1 varies with distance around the ring, assuming barriers to direct gene flow between *viridanus* and *plumbeitarsus* and across the interior of the ring. We used latitude/longitude coordinates of sampling sites (after removing the one *nitidus* site, which is outside the main ring) to generate a matrix of great‐circle distances between adjacent sites, and then used these distances to produce a matrix with distances measured only around the western, southern and eastern sides of the ring. We applied Principal Coordinates Analysis to this distance matrix, and the first PC axis was then used as a measure of location around the ring.

### 2.8 Visualisation of Genotypes

To enable visualisation of genotypic variation among individuals in specific chromosomal regions, we generated ‘genotype‐by‐individual’ plots using custom scripts. These plots show individuals in rows and SNPs in columns, with SNPs arranged in order of location on the chromosome. In each plot, individuals are ordered either according to location around the ring or according to LHBR membership group. To reduce the visual complexity of these plots and focus attention on the SNPs that are most informative about group differences, we included only those SNPs for which one variant showed greater than 50% frequency in at least one group and lower than 50% frequency in at least one group.

## 3 Results

### 3.1 Genomic Variation Around the Ring

Confirming results in Alcaide et al. (2014), principal components analysis applied to just over a million SNPs mapped to the greenish warbler genome (Table 1) shows that overall genomic variation among 257 individuals is related to geography in a way that is concordant with the ring species hypothesis (Figure 1B,C). West Siberian *viridanus* and east Siberian *plumbeitarsus* form well‐differentiated clusters which we consider to be different biological species where they meet in central Siberia, albeit with some introgression from west to east. One individual in the new sample falls within the large PCA space between *viridanus* and *plumbeitarsus* groups. As described in more detail below, this bird is a first‐generation backcross, providing the first direct confirmation that occasional hybridisation between these species is ongoing. The ring of populations to the south follows a stepwise progression from west to east through northern *viridanus* (dark blue) to southern *viridanus* (light blue) to *ludlowi* (dark and progressively lighter shades of green) to *trochiloides* (yellow) to *obscuratus* (orange) to the Beijing samples (pink) to *plumbeitarus* (red). Other gaps in the PCA distribution are likely explained in part by geographic gaps in our sampling. The overall pattern is one in which there is somewhat gradual or stepwise progression in the main axis of genomic variation, PC1, around the ring, whereas a secondary axis, PC2, changes from south to north (Figure 1).

**TABLE 1:** Assembly features of the new greenish warbler reference genome.

Assembly feature	Greenish warbler genome
Size of assembly	1.3 Gb
Scaffolds N50 size	77.9 Mb
Scaffolds N50 number	6
Longest scaffold	160.2 Mb
Contig N50 size	7.7 Mb
Contig N50 number	44
Longest contig	30.1 Mb
Complete BUSCOs^a^ (%)	99.3%

^a^ (Simão et al. 2015).

### 3.2 Haploblocks

**FIGURE 2:**
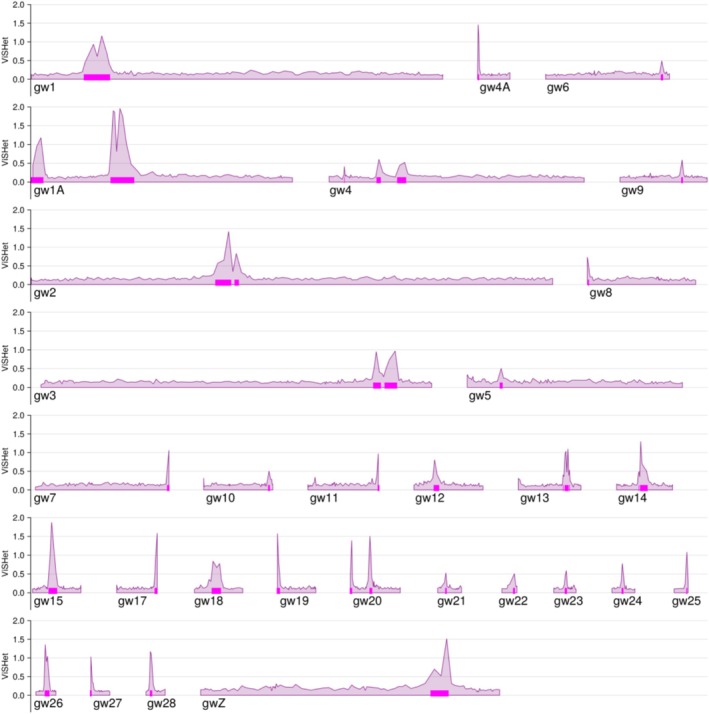
Windowed variance in standardised heterozygosity (ViSHet) varies dramatically across the genome. Magenta bars show large haplotype block regions (LHBRs), defined as windows with ViSHet > 0.4. This figure is based on genotypes of 257 greenish warblers at 1,003,924 SNPs and a window size of 500 SNPs.

Variance in standardised heterozygosity (ViSHet) varies dramatically across the genome (Figure 2). The distribution of LHBRs based on the threshold of ViSHet > 0.4 is nonrandom and significantly overdispersed. Twenty‐six chromosomes have just one LHBR, three have two, and one has three (*p* < 0.0001 by a chi‐square test comparing this distribution to that expected under the Poisson; this test is conservative because it does not account for differences in chromosome size, which would lead to a null expectation of more variance in LHBR number among chromosomes, compared to the Poisson).

### 3.3 Z Chromosome

**FIGURE 3:**
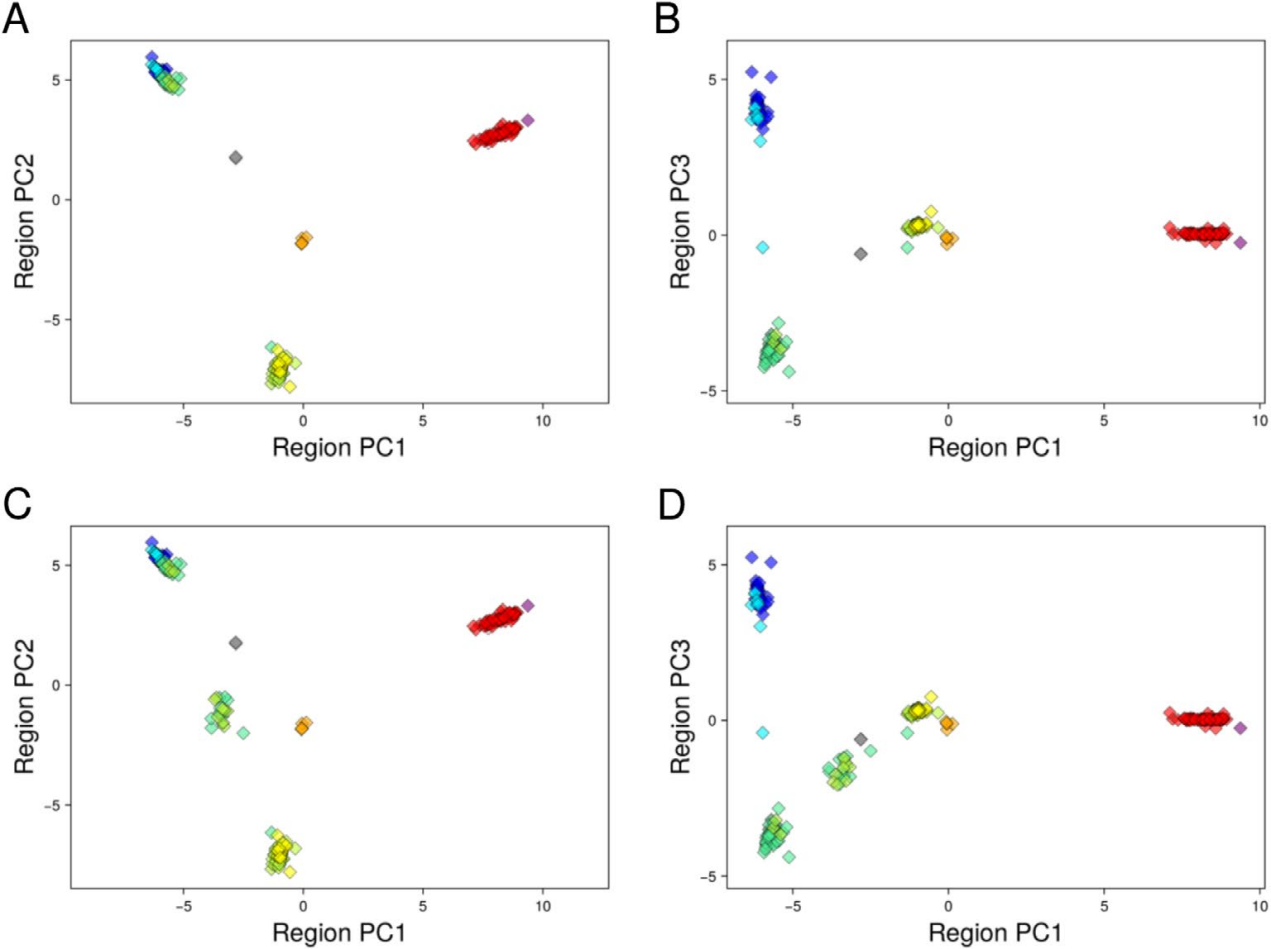
Principal components analysis (PCA) of variation in the Z chromosome LHBR. Each small diamond symbol represents a single individual, and colours correspond to sampling regions as in Figure 1. Panels A (PC1 vs. PC2) and B (PC1 vs. PC3) show only individuals with low individual heterozygosity in this LHBR. In contrast, all individuals are shown in panels C (PC1 vs. PC2) and D (PC1 vs. PC3), revealing an additional cluster that corresponds to heterozygotes between two homozygous clusters, as found in the southwest hybrid zone.

The Z chromosome is widely recognised as contributing to reproductive isolation in birds, and it often shows stronger differentiation between hybridising forms than the autosomes show (Ellegren et al. 2012; Hooper et al. 2019; Irwin 2018; Qvarnström and Bailey 2009). The one LHBR on the Z chromosome is large (5.38 Mb) and contains clearly distinct haplotype groups that are largely spatially disjunct. To show this, in Figure 3 we use PCA to visualise genetic relationships among individuals based on the variant sites in this LHBR. Graphing the low‐heterozygosity individuals for PC1 vs. PC2 (Figure 3A) and PC1 vs. PC3 (Figure 3B) demonstrates 6 separate groups corresponding to a *viridanus* type, a *nitidus* type, a northern *ludlowi* type, a southern *ludlowi* / *trochiloides* type, an *obscuratus* type and a *plumbeitarsus* type, such that the distribution of haplotypes is largely associated with geographic and taxonomic delineations. Individuals in these groups are mostly homozygous for these types when considering the entire LHBR, although some individual SNPs are heterozygous due to some variation within each major LHBR type.

**FIGURE 4:**
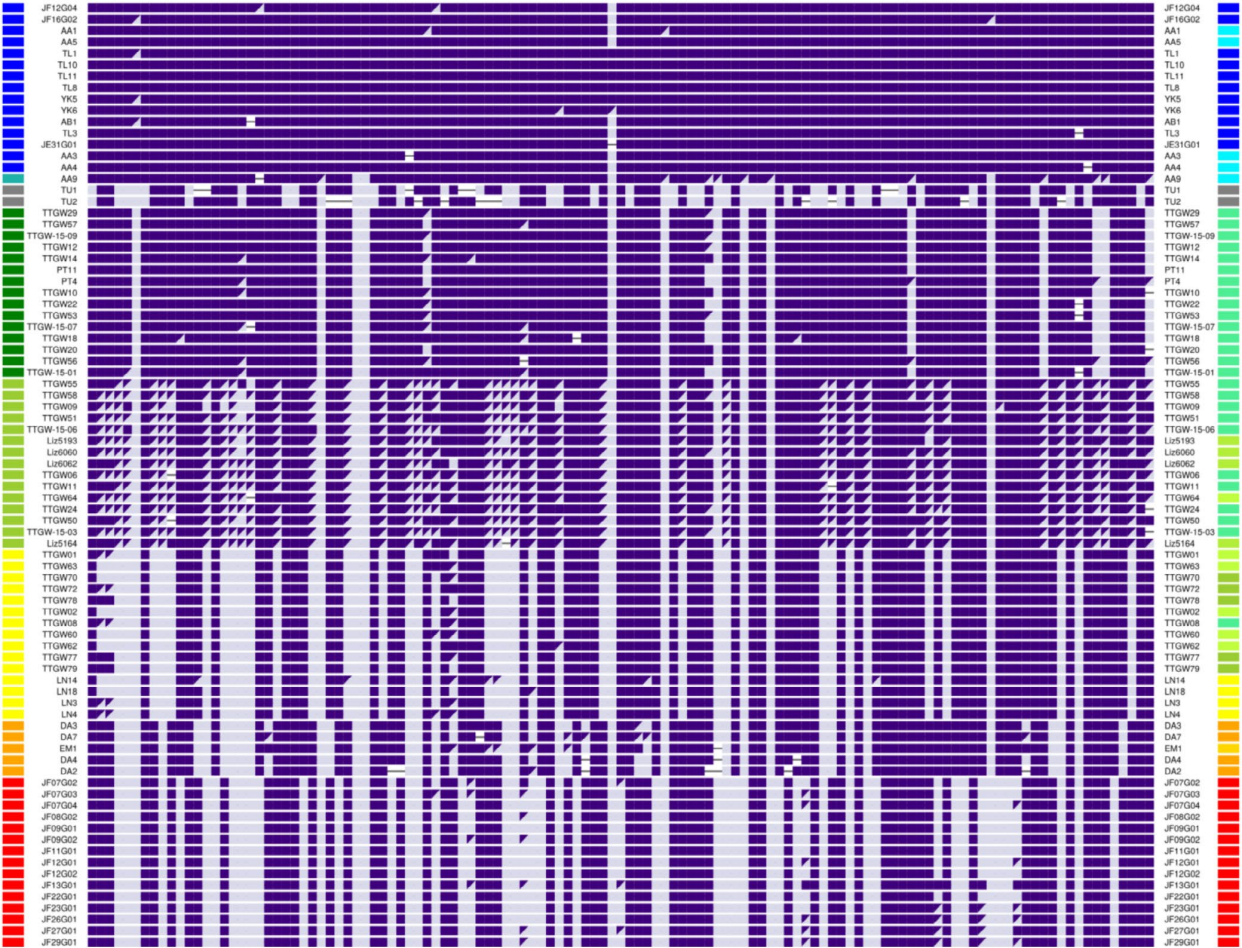
SNP genotypes within the Z chromosome LHBR, for a subsample of individuals from the PCA clusters shown in Figure 3A–C. Individuals are in rows and SNPs are in columns. Only those SNPs that are highly differentiated are shown (see Methods). Colours on the left side indicate PCA clusters, and colours on the right side indicate sampling sites. There are clear genotypic signature differences among 6 homozygous groups for this LHBR, and there is a large group of heterozygotes for the northern *ludlowi* and *trochiloides* haplotypes. To see the genotypes of all individuals in the study, see Figure S1.

When we include high‐heterozygosity individuals in the plots (Figure 3C,D), an additional cluster halfway between the northern *ludlowi* cluster and the southern *ludlowi* / *trochiloides* cluster corresponds to heterozygotes of these two LHBR types. The 7 PCA clusters (6 homozygous clusters and one heterozygous cluster) can be clearly seen as sets of linked genotypes in a genotype‐by‐individual plot (Figure 4; Figure S1). Strong haplotype structuring in this LHBR region contrasts with the much weaker geographic structuring and lack of clear haplotype groups seen in non‐LHBR regions (see Figure S2).

In the Z‐chromosome LHBR, the strong correspondence between the homozygous clusters and geographic regions points to restricted gene flow for this genomic region. However, the many heterozygotes between the highly divergent northern *ludlowi* type and the southern *ludlowi* / *trochiloides* type imply reproductive continuity between those groups and restricted recombination within the LHBR.

### 3.4 Autosomes

**FIGURE 5:**
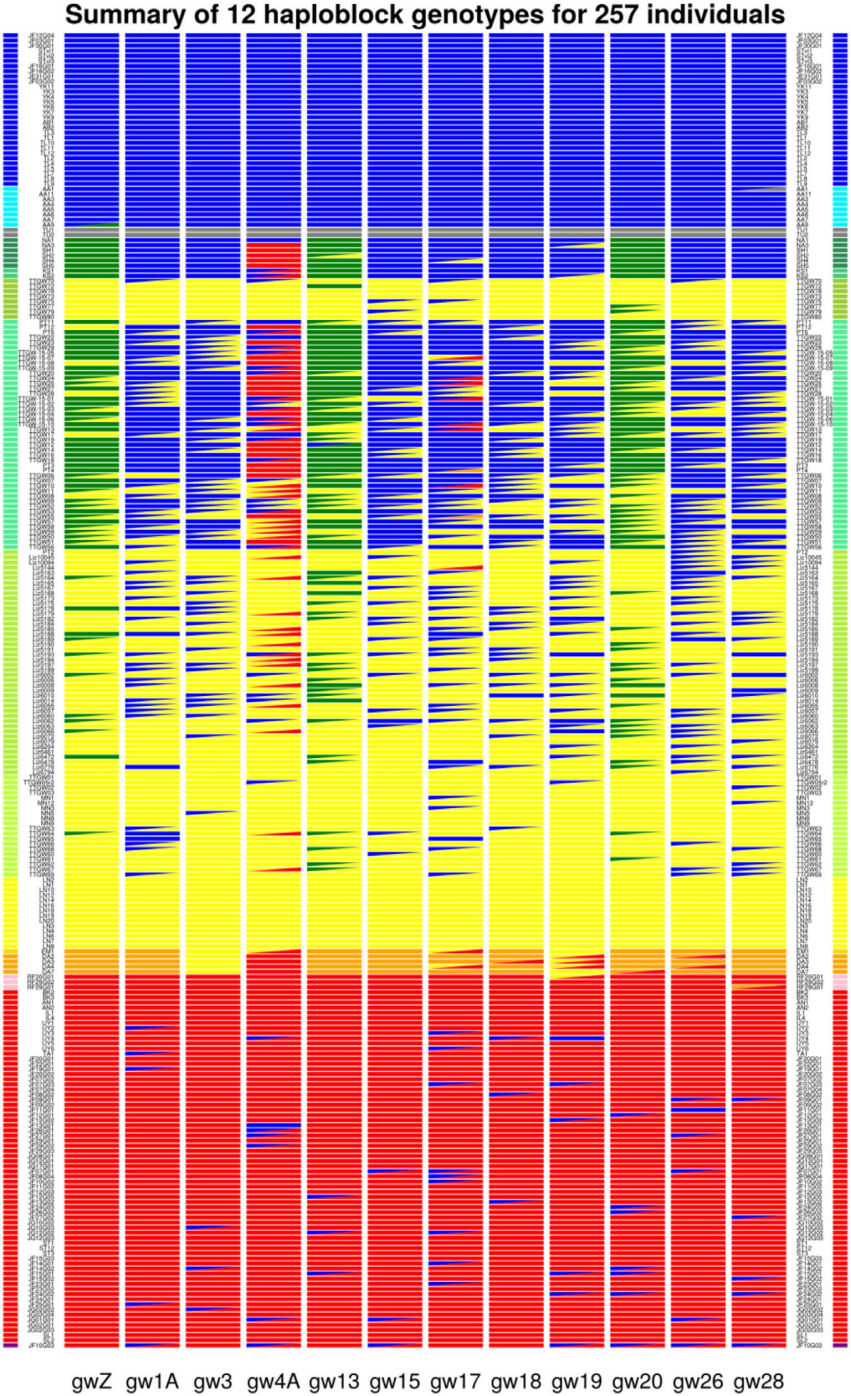
LHBR genotypes from 12 chromosomes and 257 individual greenish warblers, arranged in geographic order around the ring. Small columns of colour on the left and right sides indicate the sampling region of each individual (see map in Figure 1), with the Siberian hybrid represented by the lowest row of the figure. The 12 broad columns more central to the plot use colour to indicate LHBR genotypes of each individual (with colours representing 4 to 6 haplotypes per LHBR). LHBR homozygotes are indicated by a solid rectangle of one colour, whereas heterozygotes are represented by a rectangle split into two triangles of different colours.

We conducted similar analyses to those of the Z chromosome for the largest LHBR on 11 other chromosomes. Resulting genotypes for all individuals are illustrated in Figure 5. For each LHBR (in columns), different colours represent distinct haploblock types. Homozygotes are illustrated with a filled rectangle, whereas heterozygous LHBRs are illustrated with two triangles, each with the colour of one haploblock allele. Individuals (in rows) are arranged in their order around the greenish warbler ring of sampling locations. All these LHBRs have broadly similar aspects of their geographic pattern of haplotype distribution, with each having 4–6 distinct haplotypes. In every LHBR, there is a haplotype common in *viridanus* (haplotype coloured blue), another found in *nitidus* (grey), another common in *trochiloides* (yellow), and another common in *plumbeitarsus* (red). Nine of the LHBRs have a distinct haplotype that is common in *obscuratus* (orange). Four have yet another distinct haplotype in the northern *ludlowi* geographic region (green). The geographically adjacent *viridanus‐* and *ludlowi*‐associated haplotypes share more similarity than most other haplotypes do, being distinguished along PC3 rather than along the first two PCs. For those LHBRs for which we do not distinguish green vs. blue haplotypes, a gradient along PC3 indicates some structuring that is not clear enough to assign discretely different haplotypes.

In the following sections, we turn to consider connections between taxa, as evidenced by haploblocks.

### 3.5 Northern Overlap Zone

The great majority of the 59 new central Siberian samples cluster closely into two distinct groups (Figure 1B) corresponding to *viridanus* (30 individuals, including 7 new) and *plumbeitarsus* (69 individuals, including 51 new). However, one individual falls in the large PCA gap between the two clusters (Figure 1B). Examination of chromosome‐by‐chromosome LHBR variation of this individual compared to those in the *viridanus* and *plumbeitarsus* clusters shows that it is heterozygous for about half of the large blocks of differentiation between the two taxa and homozygous *plumbeitarsus* for the other half (see this individual in the lowest row of Figure 5). These patterns reveal that this individual is a backcross of an F_1_ hybrid and a *plumbeitarsus*.

While there is only a single recent‐generation hybrid in the dataset, there are many cases of otherwise *plumbeitarsus* individuals containing *viridanus* haploblocks across a fraction of their genomes. This can be seen in the lower part of Figure 5, which shows 34 (out of 69) of our east Siberian *plumbeitarsus* individuals have 1–3 LHBRs (out of 12) that are heterozygous or homozygous for a *viridanus* haplotype. Which of the *plumbeitarsus* individuals display *viridanus* ancestry blocks tends to be different for each chromosome. These blocks of *viridanus* ancestry explain the shape of the *plumbeitarsus* cluster in the whole‐genome PCA (Figure 1B), with the long axis of this cluster pointing towards the distant *viridanus* cluster. The evidence indicates direct introgression of *viridanus* ancestry into *plumbeitarsus*, but there is no indication of the reverse: all *viridanus* individuals form a tight cluster on these plots, and the shape of the *viridanus* PCA cluster is not pointing towards *plumbeitarsus*. The 11 autosomal LHBRs examined in Figure 5 each show between 2 and 9 instances of the *viridanus* haplotype in our sample of 69 *plumbeitarsus*, whereas the Z chromosome shows none, consistent with the general pattern of relatively limited Z chromosome introgression across avian hybrid zones. In summary, there is limited one‐way ongoing introgression between the most differentiated forms on the north side of the greenish warbler ring, as previously inferred from a genome‐wide study of SNPs (Alcaide et al. 2014) and now confirmed with the discovery of a backcross of an F_1_ and a *plumbeitarsus*.

### 3.6 Southwest Transition Zone

The pattern of consistently high differentiation between *viridanus* and *plumbeitarsus* across the northern break, with limited introgression from west to east, contrasts markedly with the much more continuous gradient of genetic signatures seen along the southwestern side of the ring (Figures 1B,C, 5). Much of this genetic gradient occurs across a roughly 200 km distance along the Chenab River in the western Himalaya, described previously as a hybrid zone (Alcaide et al. 2014). Although variation is continuous when genome‐wide SNPs are considered together, discretely different haplotypes in northwestern compared to southeastern populations are observed for each of the LHBRs examined (Figure 5). The *ludlowi* subspecies (in shades of green), which straddles the hybrid zone, contains a mixture of these types and has many individuals heterozygous for some of these haploblocks. Importantly, no individuals are heterozygous at all the LHBRs shown in Figure 5. Consequently, none show the pattern expected in a first‐generation hybrid between an individual homozygous for all blue or green LHBRs (as in northern *ludlowi*) and an individual homozygous for all yellow LHBRs (as in Nepal). Rather, the complex mix of LHBR genotypes is explained by many generations of interbreeding and backcrossing.

### 3.7 Eastern Gradient in Haplotypes

The east side of the ring also shows distinct haploblock types that are arranged in a south–north frequency gradient (Figure 5). Distinct southern *trochiloides* (yellow), mid‐latitude *obscuratus* (orange) and northern *plumbeitarsus* (red) types occur in 9 LHBRs, but only two distinct types (*trochiloides* and *plumbeitarsus*) are present in 3 LHBRs, with *obscuratus* having one or both. The five *obscuratus* samples show a variety of LHBR genotypes that all involve some types typical of *plumbeitarsus* and some types typical of *trochiloides* (see the combinations of yellow, orange and red LHBR types in the *obscuratus* individuals indicated with orange on the left and right margins). The samples from Beijing show similarity to Siberian *plumbeitarsus*, yet this group has some LHBR types that are typical of *obscuratus* (see chromosomes 19 and 28 in Figure 5). These genotypes are consistent with ongoing gene flow between *obscuratus* and *plumbeitarsus*.

### 3.8 |The Allopatric Taxon

Haploblocks are informative regarding the differentiation of the western relative, *nitidus*, from the rest of the superspecies. At all the LHBRs illustrated in Figure 5, the two *nitidus* individuals are homozygous for a distinct haplotype that shows substantial nucleotide differences from other haplotypes in the species complex. Accordingly, these *nitidus* individuals are more differentiated from *viridanus* and *ludlowi* than might be inferred from their position in the whole‐genome PCA (Figure 1B; in whole‐genome analyses *nitidus* are distinguished from other individuals along higher PC axes). While *nitidus* is clearly differentiated from other populations, its LHBR haplotypes are usually more related to those of northern *ludlowi* / southern *viridanus* than to those of other populations. One southern *viridanus* individual from Kyrgyzstan is heterozygous for *viridanus* and *nitidus* haplotypes for the chromosome 28 LHBR, implying recent genetic exchange between these groups.

### 3.9 Discordance Among LHBRs

**FIGURE 6:**
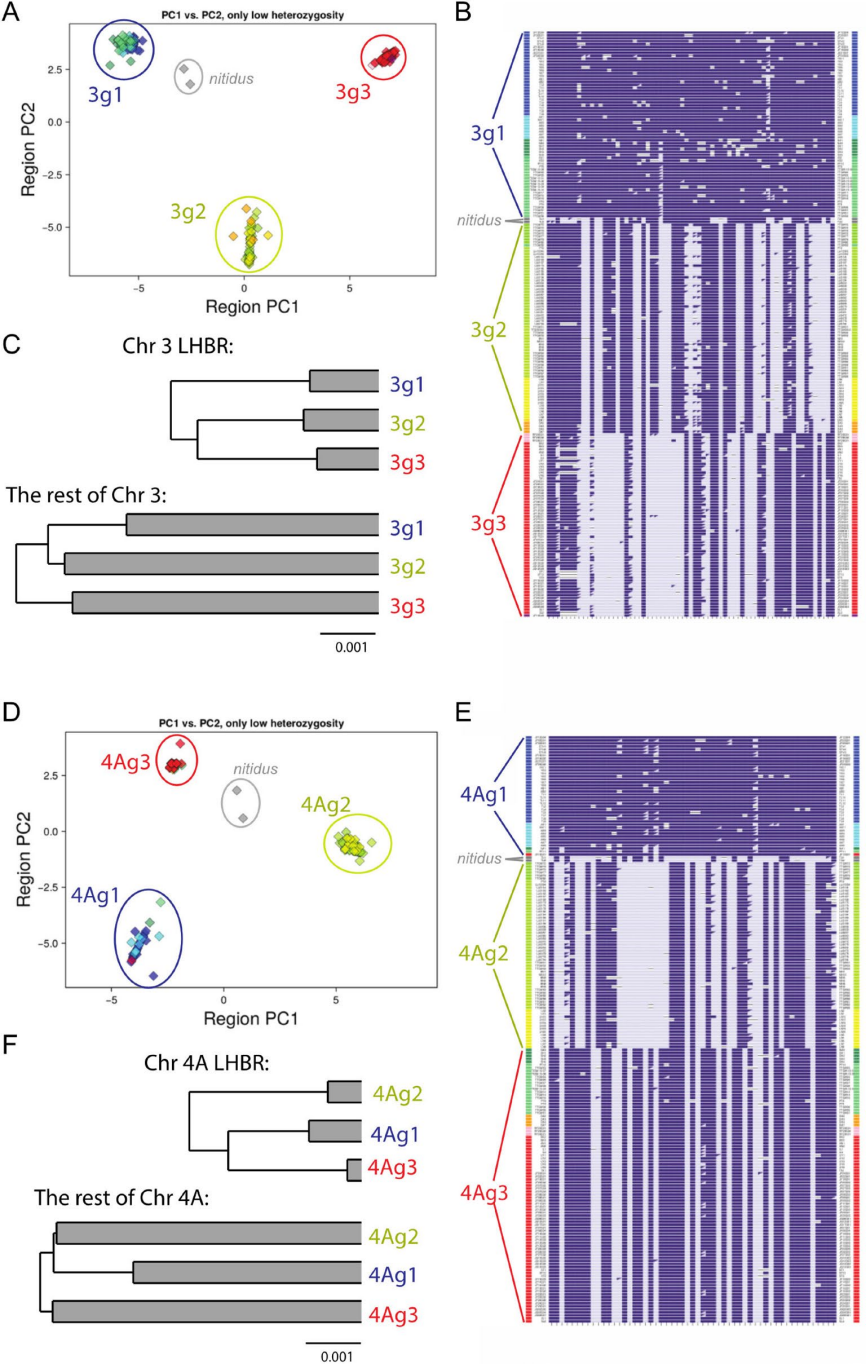
Comparison of phylogeographic variation at LHBRs on chromosome 3 (A–C) and chromosome 4A (D–F). PCAs of only low‐heterozygosity individuals (i.e., LHBR homozygotes) are shown in panels A and D, revealing that both LHBRs have four major haplotype groups, and the differences in their SNP genotypes (columns) can be clearly seen in genotype‐by‐individual plots (B, E) in which individuals (rows) are arranged according to haplotype group rather than geographic location (which is indicated by colours on the left and right of each row). Phylogenies based on within‐group (shaded portions) and between‐group pairwise nucleotide distance (π and *D*_xy_) show that within‐group variation, between‐group distance, and the ratio of within‐group to between‐group distance are lower in the LHBRs than on the rest of the chromosome. However, two LHBRs show differing relationships and distributions of the major groups. Haplogroup 4Ag3 is shared among *plumbeitarsus*, *obscuratus* and many northern *ludlowi* individuals, and has extremely low within‐group variation.

While overall there is much similarity among LHBRs in their geographic structure (Figure 5), some LHBR haplotypes show strongly discordant geographical distributions. In Figure 6 we compare variation in two example LHBRs, one (on chromosome 3; Figure 6A–C) representing a common pattern and the other (on chromosome 4A; Figure 6D–F) showing an unusual one. For each LHBR, the figure illustrates PCA locations and SNP genotypes for those individuals classified as homozygous within each LHBR, revealing that each of these LHBRs has 4 distinct haplotypes. The general pattern of biogeographic clustering of genotype groups seen at the chromosome 3 LHBR is representative of many of the LHBRs on other chromosomes (Figure 5). In contrast, the chromosome 4A LHBR has one haplotype group (4Ag3) that is found in geographically disjunct locations: it is common in *plumbeitarsus* and *obscuratus* in the east but also present at high frequency in northern *ludlowi* in the west. When considering whole‐genome or phenotypic variation, the northern *ludlowi* and *plumbeitarsus* groups are generally highly divergent (Figure 1B; Irwin, Bensch, et al. 2001; Irwin et al. 2008), making this close relationship at the chromosome 4A LHBR surprising.

### 3.10 |Differentiation Within LHBRs

Continuing with the chromosome 3 and 4A LHBRs, we compare amounts of nucleotide differentiation within and between haplotype groups, and inside and outside the LHBR (Figure 6, Table 2). For chromosome 3, mean π (the average within‐group pairwise nucleotide distance) across the three haplotype groups is 76% lower than the mean π for the rest of the chromosome, and the mean *D*_xy_ (the between‐group pairwise nucleotide distance) is 43% lower than the rest of the chromosome (Table 2, Figure 6C). Values for the chromosome 4A LHBR are 88% lower mean π and 49% lower mean *D*_xy_ than the rest of the chromosome. Notably, the widely distributed 4Ag3 genotype group, which is present in both the east and west regions but not in between, has a value of π that is extraordinarily low, 95% lower than the rest of the chromosome and much lower than the π values of the other haplotype groups (Table 2). Figures 6C,F show these patterns using phylogenies based on these distances. Relationships between locations are derived from (*D*_xy_) with (π) shaded in the tip edges. We argue in the Discussion that the low coalescence times indicated by these low π and *D*_xy_ values of these geographically widespread LHBR haplotypes imply selection is involved in their spread.

**TABLE 2:** Within‐group variance, π (on diagonals) and between‐group distance, *D*_xy_, on the off‐diagonal for chromosomes 3 and 4A, comparing three haplotype groups for each (Figure 6). Patterns inside the LHBR are separated from those on the rest of the chromosome.

	Inside the LHBR			Outside the LHBR		
	**3g1**	**3g2**	**3g3**	**3g1**	**3g2**	**3g3**
3g1	0.00125			0.00457		
3g2	0.00354	0.00136		0.00599	0.00569	
3g3	0.00398	0.00328	0.00112	0.00660	0.00653	0.00555
	**4Ag1**	**4Ag2**	**4Ag3**	**4Ag1**	**4Ag2**	**4Ag3**
4Ag1	0.00096			0.00413		
4Ag2	0.00318	0.00061		0.00556	0.00552	
4Ag3	0.00242	0.00306	0.00026	0.00557	0.00601	0.00559

## 4 Discussion

Our method of identifying chromosomal regions that have large well‐differentiated haploblock types elucidates patterns of past differentiation and gene flow between populations of greenish warblers. Each of the large haploblock regions (LHBRs) we have identified shows 4–6 divergent haplotypes that are strongly geographically localised. These results, together with biogeographic evidence that the history of Asia has involved many climatic cycles that likely separated populations for varying periods of time (Zhou et al. 2023), suggest that at least some haplotypes have arisen in genomic regions of low recombination when populations were in allopatry. This is most clear in the west Siberian *viridanus* samples, which have entirely *viridanus* homozygosity at the examined LHBRs, and the Nepal *trochiloides* samples, which have entirely *trochiloides* homozygosity. Given a role for population differentiation in the production of LHBR haplotypes, dispersal and gene flow are implicated in the production of currently observed heterozygotes. Contemporary evidence comes from the occasional presence of haplotypes outside of their normal locations (e.g., typically *viridanus* haplotypes in *plumbeitarsus*; a typically *nitidus* haplotype in *viridanus*), plus the more extensive overlap zones in the regions of *obscuratus* and *ludlowi*. We discuss the origins of the haploblocks first, then how they inform the history of the superspecies complex.

### 4.1 Causes of the LHBRs

In the Introduction, we articulated the ‘inversion’ and ‘sweep‐before‐differentiation’ hypotheses for the development of haploblocks, and we explained how these predict different ages of coalescence of haploblocks compared to the rest of the genome. Comparisons of phylogenies in the example LHBRs at chromosomes 3 and 4A show a pattern predicted by sweep‐before‐differentiation, with much lower variation both within and between haploblocks in the LHBR than elsewhere on each chromosome. More broadly, the strong associations seen between high ViSHet, high *F*_ST_ and low *D*_xy_ (Figure S34; Irwin et al. 2016) indicate that LHBRs tend to have lower coalescence times than the rest of the genome, a pattern consistent with sweep‐before‐differentiation but not the inversion hypothesis. Moreover, under the inversion hypothesis, we would need to invoke multiple inversion events with similar breakpoints to explain the presence of 4–6 major haplotypes at an LHBR. In contrast, under the sweep‐before‐differentiation hypothesis, a region of inherently low recombination does not require any rare events to evolve a variety of divergent haplotypes, especially when geographic separation is involved.

Exactly one LHBR in most greenish warbler chromosomes suggests that many LHBRs are centromeric regions, which are thought to have restricted recombination when compared to other regions of chromosomes (Bascón‐Cardozo et al. 2024; Logsdon et al. 2024). While proper evaluation requires assessment of centromere position in the greenish warbler, we aligned the LHBR regions to the zebra finch genome (see details in Supporting Information, including Table S2) using minimap2 (https://github.com/lh3/minimap2) optimised for 5% sequence divergence. We define close to the centromere as within 15% of the total chromosome length, based on zebra finch centromere positions given by Takki et al. (2022). Out of the 38 LHBR regions located on chromosomes with known centromere positions, 16 were closely linked to the centromere of 14 chromosomes, including the Z (gw2 and gw13 each had two closely linked LHBRs). A further 7 LHBRs on acrocentric chromosomes were closely linked to a telomere. Structural rearrangements between the greenish warbler and zebra finch (Hooper and Price 2017) as well as mapping errors make this a conservative figure, implying that many LHBRs are associated with centromere or telomeric chromosomal locations. Earlier studies on a pair of flycatchers ( *Ficedula albicollis* and *F. hypoleuca* ) and two subspecies of rabbits ( *Oryctolagus cuniculus* ) have also implicated centromeric regions as especially well differentiated between populations (Carneiro et al. 2014; Ellegren et al. 2012).

The pattern of both low within‐group variation and low between‐group variation seen at the LHBRs (Figure 6) can be explained if these regions experience recurrent selective sweeps that reduce variation, originally throughout the whole species complex and subsequently regionally (Cruickshank and Hahn 2014; Irwin et al. 2016, 2018). When an advantageous variant arises and undergoes a sweep, it spreads from its geographic origin and goes to high frequency over a certain geographic region. If different sweeping variants spread in different locations, they may eventually meet in a contact zone. Perhaps one outcompetes the other and spreads further, but an alternative is that they form a stable contact zone. This could be due to each having higher fitness in the location it expanded from, and neither having an advantage over the other in an intermediate ecological area. Alternatively, the two variants could have intrinsic incompatibilities with each other, causing low fitness of heterozygotes. Either way, the result is geographic regions over which different variants are nearly fixed and other regions where there is a genetic transition with many heterozygotes for the divergent haplotypes. Little evidence of within‐LHBR recombination in the contact zone can be due to suppressed recombination in that genomic region and/or to low fitness of recombinants, if there are multiple genes that have epistatic effects on fitness. This process of selective sweeps spreading and meeting each other may also be influenced by past biogeographic restrictions in gene flow between populations.

One regularly proposed mechanism of producing sweeps is that of meiotic drive of centromeres, the location where spindle fibres attach to chromosomes during cell division (Clark and Akera 2021; Iwata‐Otsubo et al. 2017). During the first cell division of female meiosis, only one of the resulting cells will divide further to produce an egg, and there are asymmetries in cell structure prior to this cell division. If one version of a centromere is more efficient than others at connecting to the spindle fibres leading to the egg, then it will have a transmission advantage (Chmátal et al. 2014). Suppressors of this advantage can evolve, leading to repeated cycles of mutations and sweeps of meiotic drivers and suppressors (Kumon et al. 2021; Meiklejohn et al. 2018), and inherent incompatibilities where different drivers and suppressors meet.

This explanation of greenish warbler LHBR biogeography builds on the idea that low but nonzero levels of gene flow between populations can allow differentiation at neutral parts of the genome while still facilitating the spread of advantageous variants that reduce differences between populations (Morjan and Rieseberg 2004; Rieseberg et al. 2004; Rieseberg and Burke 2001). In fact, Rieseberg and Burke (2001) make the case that a species can be thought of as a group in which selective sweeps are likely to spread through the whole group, whereas different species will not experience the same selective sweeps. Of course, intermediate cases are possible, in which some sweeps can cover larger geographical regions than others. Rieseberg et al. (2004) note that ring species are situations in which this is especially likely: small rates of movement between neighbouring populations in a ring allow enough gene flow for broadly adaptive variants to spread over large regions, whereas neutrally or locally adapted variants can differentiate at finer scales. Genomic variation in greenish warblers supports this model whereby some regions of the genome show distinct geographic variants, each of which shows little within‐haplotype variation across a broad region, and there are some important differences between the geographic structuring displayed by LHBRs on different chromosomes.

Alternatives to selective sweeps include drift or strong background selection on parts of the genome. We think these explanations are unlikely to fully explain the patterns seen in the greenish warbler LHBRs. First, given that the greenish warbler species complex consists of six phenotypically and genomically differentiated subspecies spread across a huge and ecologically variable continental region, it is reasonable to think that much positive selection has been involved in shaping greenish warbler genomes. Second, LHBRs tend to have dramatically higher levels of relative differentiation (*F*_ST_) between major greenish warbler geographic regions than most of the genome does (Figures S33–S34), so they are excellent candidates for regions under selection. Third, the wide geographic distribution of some LHBR haplotypes is not predicted under a drift or background selection scenario, especially given the amount of discordance between the patterns at different LHBRs. Drift tends to cause differences between populations, whereas LHBR haplotypes show little nucleotide variation (compared to other parts of the genome) over large geographic regions. Background selection, in which deleterious mutations are selected against, should not prevent neutral mutations from building up between populations that have otherwise limited gene flow.

### 4.2 Biogeographic History of the Ring

While variation in LHBRs around the ring (Figure 5) is broadly consistent with overall genomic variation (Figure 1B) in showing *viridanus* and *plumbeitarsus* as being the most divergent forms in the ring of greenish warbler populations, LHBRs are particularly useful in showing current genetic connections between populations around the ring. We discuss these connections below, first considering the northern contact zone and then contrasting this with the southwest transitional zone and the eastern distributional gap.

### 4.3 Northern Meeting of Two Species, With One‐Way Introgression

The two Siberian forms are strongly differentiated both genomically and phenotypically despite contact in central Siberia and are clearly best described as distinct species where they are in geographic contact. Despite this, haploblock sharing shows that limited gene flow is ongoing. This gene flow is consistently asymmetric: *viridanus* receives no direct input from *plumbeitarsus*, whereas *plumbeitarsus* receives alleles from *viridanus*. The blocks of *viridanus* introgression are often large (e.g., well more than tens of millions of bp on some chromosomes; Figures S3–S32), suggesting that there is selection against them and that successful recombination is rare (Sedghifar et al. 2016; Veller et al. 2023). Importantly, we have discovered a first‐generation backcross individual, which was caught in the spring and had therefore survived for at least 10 months, and both it and the F_1_ parent must have navigated a long‐distance migration to and from wintering grounds in south Asia. The implication is that these taxa can successfully hybridise, but successful hybridisation is rare (i.e., one backcross individual out of 93 samples in the central Siberian contact area) and allows gene flow in only one direction. Detecting limited hybridisation has been generally difficult through conventional means such as direct observation of pairs or putative hybrids (Ottenburghs 2023). Nevertheless, ongoing gene flow may be important in the context of speciation and adaptation, given that adaptive alleles in one taxon can rise to high frequency and then be regularly introduced to the other at a rate many times higher than the mutation rate. Large sample sizes analysed using genomics are a most promising route to evaluate levels of hybridisation and its consequences.

### 4.4 Contrast of Southwestern and Eastern Transition Zones

Species‐level reproductive isolation in the north contrasts with weaker reproductive isolation at other zones of genomic transition around the ring. In the southwestern part of the ring, within the subspecies *ludlowi*, we find a particularly steep pace of change in relation to geography, with many chromosomes showing distinct northwestern and southeastern haploblock types. Many individuals are heterozygous for haploblocks on some chromosomes and homozygous for haploblocks on other chromosomes (Figure 5), and variation among individuals is continuous when all chromosomes and SNPs are combined (Figure 1B,C). Together, these patterns show that there is not strong reproductive isolation in the *ludlowi* transition zone. However, the maintenance of large divergent haploblock groups with few observed recombinants within these blocks is suggestive of low recombination and/or somewhat reduced fitness of recombinants, consistent with the limited zone of transition.

The geographic gap between *obscuratus* and *plumbeitarsus* (Figure 1) also corresponds to an area of steep genomic transition. Nonetheless, haploblock sharing on either side of the gap indicates some gene flow. Furthermore, *obscuratus* has genomic characteristics that are largely intermediate between *trochiloides* in the south and *plumbeitarsus* in the northeast, while also showing some signature of its own independent evolution. The Beijing individuals are further on the PC1 axis from *viridanus* than the central Siberian *plumbeitarsus* cluster is, which can be understood to result from the central Siberian *plumbeitarsus* being pulled slightly towards the *viridanus* cluster by the introgression from *viridanus*.

While the southwestern and eastern transition zones described above are widely separated and involve mostly independent transitions (*viridanus*‐*ludlowi*‐*trochiloides* vs. *trochiloides*‐*obscuratus*‐*plumbeitarsus*), our data also indicate some relatively recent gene flow on two chromosomes between northern *ludlowi* and *obscuratus*/*plumbeitarsus*. This is seen in the sharing of LHBR haplotypes on chromosomes 4A and 17 (see red haplotypes in Figure 5). This gene flow might have occurred through long‐distance dispersal of an individual between these regions or may have occurred through gradual multi‐generational gene flow through the *trochiloides* population. That we see no evidence of these haplotypes in our Nepal sample of *trochiloides* suggests that long‐distance dispersal may be the best explanation. Whichever way these haplotypes moved between these distant regions, we think that their wide geographic range and low within‐haplotype variation are best explained by selection favouring the expansion of these haplotypes over these geographic regions.

To investigate the genes involved in the unusual chromosome 4A LHBR geographic distribution, we used Liftoff (https://github.com/agshumate/Liftoff) to transfer known annotations from the zebra finch to the greenish warbler genome. We obtained a list of 20 genes in this region (see Supporting Information). It is possible that selection on one or more of these genes has contributed to the unusual geographic distribution of the haplotype shared between northern *ludlowi* and *obscuratus*/*plumbeitarsus*.

### 4.5 Gene Flow Around the Ring

One question regarding ring species is the extent to which gene flow limits differentiation (Kuchta and Wake 2016). The haploblock distributions are consistent with selection‐driven differentiation as well as gene flow influencing genetic variation of populations. Even the small amount of hybridisation and backcrossing from *viridanus* into central Siberian *plumbeitarsus* is noticeably influencing genomic variation in *plumbeitarsus*. It is likely then that the unique genomic clustering of central Siberian *plumbeitarsus* is a balance between divergent adaptation and gene flow from two sources: limited introgression from *viridanus* from the west, and gene flow from the rest of the *plumbeitarsus* range from the east and southeast, for example, the Beijing area, which receives genetic input from *obscuratus* further south. At each point around the ring, the pattern of a progression in local high‐frequency haploblocks with some haploblock sharing between neighbouring populations points to a mix of selection and gene flow in shaping greenish warbler genomes. While in the present analysis we have focused on the small fraction of the greenish warbler genome that has strongly differentiated haploblocks, lower relative differentiation between populations at many other parts of the genome is consistent with higher gene flow around the ring in those regions. Overall, haploblock variation around the greenish warbler ring shows the influence of both geographic variation in selective forces as well as the moderating effects of gene flow on population differentiation.

### 4.6 Reproductive Isolation

Genomic regions of low recombination, including both inversions and centromeric regions, have been widely implicated in speciation. Here we have identified well‐differentiated large genomic regions that are distributed geographically, pointing to an important role for divergence in inherently low‐recombining genomic regions as a driver of differentiation. Such regions may often be near centromeres, potentially subject to meiotic drive. Previous suggestions of the cause of post‐mating isolation across the northern break have included intermediate migratory behaviour (Irwin and Irwin 2005; Justen et al. 2021) and the difficulties of establishing a territory given song differences (Scordato 2018). Both these mechanisms appear leaky, given that a backcross male has been detected on the breeding grounds, and mixed singers are regularly observed (Irwin 2012; Kovylov et al. 2012) (the backcross male was singing a mixed song when caught). Our analysis suggests the possibility that genetic incompatibilities also contribute. If such incompatibilities accumulated around the ring are associated with haploblocks, then the northern contact zone would contain more intrinsic incompatibilities than do the two populations on either side of transition zones to the south (for an example of such stepwise build‐up of incompatibilities among three taxa, see Hermansen et al. 2014; Trier et al. 2014).

### 4.7 Conclusion

In this study, we have emphasised the role of geographic differentiation in the origin and spread of haploblocks, and how they subsequently introgress between taxa through hybridisation to varying degrees, as well as occasional long‐distance dispersal events. Our findings emphasise a likely important role in both transferring adaptive material between taxa, as well as contributing to reproductive isolation between taxa. Further clarification of genomic location and identification of gene content in haploblocks will improve understanding of the role of geographic differentiation, gene flow and intrinsic incompatibilities in speciation.

## Author Contributions

D.I. and T.D.P. conceived and designed the study, with contributions from S.B., G.D., J.H.I., B.H., I.M.M. and S.K.G. New samples were acquired by G.D., P.H., S.K.G., V.V.I., I.M.M., S.S., Y.W., S.Z. and T.D.P.; DNA extraction and amplification were conducted by A.G., S.K.G., A.S., and B.H.; and A.G. prepared GBS libraries. Sequencing and assembly of the reference genome were overseen by Y.N.; C.C. annotated the reference genome. D.I. performed the bioinformatic analysis of GBS reads and wrote Julia language scripts for summarising data and producing figures. B.H. analysed LHBR gene content and locations with respect to centromeres. The manuscript was drafted by D.I. and T.D.P., and all authors contributed to revisions.

## Conflicts of Interest

The authors declare no conflicts of interest.

## Benefit‐Sharing Statement

This paper is the result of a large international collaboration between scientists from eight countries, five of which are within the range of the study system, the greenish warblers in Eurasia. All collaborators are included as coauthors, and the results of the research are being shared openly with scientists and the broader community in those countries and beyond, via this paper and via the sharing of data and software as described in the Data Accessibility Statement.

## Supporting information


Data S1.

Table S2.

## Data Availability

The new *Phylloscopus trochiloides* reference genome is provided at NCBI under PRJNA1210605. New GBS reads have been deposited at NCBI SRA under accession PRJNA1207594; within this accession are data for 3 sets of samples: runs SRR31958018, SRR31958020, and SRR31958019. This study also used GBS reads from a previously‐sequenced set of samples, run SRR1176844 from accession PRJNA238841 (Alcaide et al. 2014). Genotype calls, sample metadata, and processing scripts are available at this Dryad archive: https://doi.org/10.5061/dryad.8w9ghx3xr. Julia functions used in data processing and graphing are provided in the new GenomicDiversity.jl package (https://github.com/darreni/GenomicDiversity.jl), and the complete analysis scripts with explanatory comments is at a GitHub Pages site (https://darreni.github.io/GreenishWarblerGenomics2025) and at the Dryad archive and at a Github repository (https://github.com/darreni/GreenishWarblerGenomics2025).
