# Supplementary material for: The Distribution and Dispersal of Large Haploblocks in a Superspecies
Source: Mol Ecol. 2025 Mar 17;34(21):e17731. doi: 10.1111/mec.17731 (PMC12573742; doi:10.1111/mec.17731)
Supplement: Supplementary file 1 — Data S1. [file MEC-34-e17731-s002.pdf]

Supplemental Information for:

The distribution and dispersal of large haploblocks in a superspecies.

Darren Irwin, Staffan Bensch, Caleigh Charlebois, Gabriel David, Armando Geraldes, Sandeep Kumar Gupta, Bettina Harr, Paul Holt, Jessica H. Irwin, Vladimir V. Ivanitskii, Irina M. Marova, Yongchao Niu, Sampath Seneviratne, Ashutosh Singh, Yongjie Wu, Shangmingyu Zhang, Trevor D. Price

Supplementary Table S1. Sampling sites and sample sizes (total of 257 individuals, plus the reference genome).

Location	Code	Samples	Subspecies	Latitude N	Longitude E	Ring location (km)
Stolbi *viridanus*	ST_vi	6	*viridanus*	55.9	92.6	-4980.5
Divnogorsk *viridanus*	DV_vi	2	*viridanus*	56.0	92.4	-4978.8
Verkh. Biryusa *viridanus*	VB_vi	2	*viridanus*	55.9	92.0	-4953.3
Yekaterinburg	YK	7	*viridanus*	56.8	60.6	-4799.4
Abakan	AB	2	*viridanus*	52.0	89.5	-4548.4
Teletsk Lake	TL	11	*viridanus*	51.7	87.6	-4428.8
Ala Archa	AA	8	*viridanus*	42.5	74.5	-3027.0
Turkey	TU	2	*nitidus*	41.0	42.0	n/a
Shogran	SH	4	*ludlowi*	34.6	73.5	-2171.6
Naran	NR	2	*ludlowi*	34.9	73.7	-2166.6
Overa	OV	2	*ludlowi*	34.0	75.2	-1998.2
Satharundhi	SA	8	*ludlowi*	33.0	76.2	-1861.9
Killar	KL	3	*ludlowi*	33.1	76.4	-1854.7
Sural	SR	18	*ludlowi*	33.1	76.5	-1852.6
Thalighar	TH	7	*ludlowi*	32.8	76.4	-1835.4
Tindi	PA	2	*ludlowi*	32.8	76.5	-1830.4
Sukhto	SU	6	*ludlowi*	32.9	76.9	-1805.4
Nainaghar	NG	9	*ludlowi*	32.7	76.9	-1797.0
Mooling	ML	44	*ludlowi*	32.5	77.0	-1774.6
Manali	MN	10	*ludlowi*	32.2	77.1	-1747.3
Spiti	SP	10	*ludlowi*	32.4	77.3	-1743.1
Langtang	LN	14	*trochiloides*	28.2	85.5	-830.7
Gongga	GG	1*	*obscuratus*	29.5	102.0	783.7
Emeishan	EM	1	*obscuratus*	29.5	103.3	890.4
Xining	XN	4	*obscuratus*	37.0	102.0	1481.8
Beijing	BJ	3	*plumbeitarsus*	40.0	115.5	2480.1
Baikal	BK	2	*plumbeitarsus*	51.9	104.9	4027.0
Arshan	AN	2	*plumbeitarsus*	51.9	102.5	4134.3
Ilinka	IL	2	*plumbeitarsus*	51.1	95.5	4451.8
Uyukski	UY	6	*plumbeitarsus*	51.9	94.1	4581.1
Tuva	TA	1	*plumbeitarsus*	51.3	92.0	4673.0
Manskoe Belogorie	MB	4	*plumbeitarsus*	54.7	94.0	4762.5
Stolbi *plumbeitarsus*	ST_pl	35	*plumbeitarsus*	55.9	92.6	4913.2
Stolbi hybrid	ST_hyb	1	hybrid backcross	55.9	92.6	4913.2
Divnogorsk *plumbeitarsus*	DV_pl	5	*plumbeitarsus*	56.0	92.4	4929.8
Verkh. Biryusa *plumbeitarsus*	VB_pl	5	*plumbeitarsus*	55.9	92.0	4943.4
Predivinsk	PR	5	*plumbeitarsus*	57.1	93.5	4951.7
Solgonski	SL	2	*plumbeitarsus*	55.7	91.0	4982.0

* The reference genome was generated from this individual.

Naming of reference genome scaffolds

For scaffolds in our reference genome that show clear homology to the zebra finch *Taeniopygia guttata* genome (version 3.2.4; Genbank accession ABQF01000000), we designated the greenish warbler scaffold as "gw#" where "#" is the name of the zebra finch chromosome. This was the case for chromosomes gw1 – gw15, gw17 – gw28, gw1A, gw1B, gw4A, and gwZ. Additional scaffolds in the greenish warbler assembly, which were in most cases quite small, were designated as "gws###" where "###" is a number 100 or above, in descending order of scaffold length.

Filtering to obtain true Z chromosome SNPs

Initial inspection of the PCA plot for the Z chromosome revealed an unusual pattern (compared to other scaffolds) that we suspected to be due to differences between females, which have one Z and one W chromosome, and males, which have two Z chromosomes. Some GBS sequences from the nonrecombining part of the W chromosome can map to regions on the Z that share ancient homology, and if the Z and W sequences have a different nucleotide at one site in an otherwise similar sequence, the different Z and W variants can be mistaken for a heterozygous Z genotype. Because males do not have a W, they would not display the allele from the W. To check for this problem and remove the SNPs that are responsible, we did the following.

First, we used vcftools to calculate mean sequencing depth for SNPs that map to the Z chromosome markers and for those that map to chromosome 2, the largest autosome. Among individuals, the ratio of chromosome Z read depth to chromosome 2 read depth showed a strongly bimodal distribution, with one peak at about 1.1 and another at about 0.65, with no individuals falling in between (in the 0.7 to 1.0 range). This is consistent with individuals at the higher ratio being males and the lower ratio being females. (The female ratio is higher than 0.5 because some Z chromosome SNPs are in the pseudoautosomal region and thereby have recombining Z and W versions; the male ratio is higher than 1 likely because of more repetitive regions on the Z than on the autosomes.)

Using those ratios to determine sex, we then examined a graph showing, for all SNPs, the proportion of females that were heterozygous vs. the proportion of males that were heterozygous. True Z chromosome markers should show no female heterozygosity or, in the case of pseudoautosomal loci, similar heterozygosity in males and females. While the great majority of markers met those expectations, some SNPs had high female heterozygosity and low male heterozygosity, a pattern expected of divergent Z and W variants incorrectly mapped to a single Z locus. We removed these loci from the dataset by applying this rule: If female heterozygosity was above 0.05 and the ratio of female to male heterozygosity was greater than 2, then we removed the SNP. We also removed loci with male heterozygosity above 0.5, as those are likely due to duplications being treated as a single locus. This filtering removed 1,831 Z-chromosome loci, leaving 51,505 remaining. Imputation and PCA plotting was then performed on this filtered

Z-chromosome dataset, which resulted in a PCA plot that included both sexes but no longer showed a sex difference Z chromosome relatedness.

Localizing LHBRs with respect to centromere position

We extracted zebra finch centromere genomic positions from Takki et al. (2022). Chromosomes 1B, 16 and 27 do not have known centromeres. The 39 LHBRs identified in this study range in size from 140kb – 7.8Mb, and together account for 63.9Mb of the greenish warbler genome. Chromosome 27 LHBR was removed from the analysis due to lack of known centromere position.

We extracted fasta sequences for all LHBRs from the greenish warbler genome sequence. We used minimap2 (https://github.com/lh3/minimap2) with -asm 5 option (optimized for 5% sequence divergence) to align these sequences to the repeat masked zebrafinch genome downloaded from ENSEMBL (https://useast.ensembl.org/Taeniopygia_guttata/Info/Index?db=core). Alignments were filtered for mapping quality equal to 60, and number of bases matching between the two species >=1000bp, resulting in 480 alignments across the 39 LHBR regions. Note that alignments are interrupted by presence of repeats (which are not possible to align uniquely) and the presence of genomic rearrangements, insertions, and deletions between the species. With the exceptions of two alignments, which mapped to different chromosomes, all 480 retained greenish warbler alignments mapped to the corresponding chromosome and genomic region in the zebra finch.

Having identified genomic coordinates of warbler LHBRs in the zebra finch, we next calculated the distance for each alignment from the known centromere. We defined an LHBR region to be closely linked to a centromere if at least one alignment within the LHBR region was within 15% of the total chromosome length. Similarly, we defined an LHBR to be closely linked to a centromere opposing telomere if at least one alignment within the LHBR was further away from the centromere than 85% of the total chromosome length. Assignment to telomeres is only possible for those chromosomes with acrocentric centromeres.

Characteristics of alignments within each LHBR are shown in Supplementary Table S2 (as a separate Excel file), together with their distance from the centromere, expressed in both absolute number of base pairs as well as in % of total chromosome length.

Out of the 38 LHBR regions located on chromosomes with known centromere positions, 16 were closely linked to the centromere of 14 chromosomes, including the Z (note that gw2 and gw13 each had two closely linked LHBRs). A further 7 LHBRs were closely linked to the telomere based on distance from an acrocentric centromere. This implies that many LHBRs are associated with centromere or telomeric chromosomal locations. The results are summarized below:

LHBR regions closely linked to centromere

gw1A_4674_3771263
gw2_54537375_59262130 & gw2_60234161_61533451
gw5_10095304_10956815

gw6_34584054_35259663
gw7_40285518_41004802
gw9_18659213_19160528
gw10_19303093_19842830
gw11_20986644_21509352
gw13_13574177_13722280 & gw13_14099239_15243036
gw14_7443251_9703930
gw17_11771676_12624044
gw20_27354_721651
gw25_5185626_5473966
gwZ_68372986_73749599

LHBR regions far from the acrocentric centromere

gw1_15689747_23478124
gw8_399852_757312
gw12_6356015_7992323
gw18_6079784_8833872
gw24_3468239_4001782
gw26_4153299_5549635
gw28_1822776_2522648

Gene annotation for the chromosome 4A LHBR

The chromosome 4A LHBR encompasses a ~400kb region that localizes to the 14.9Mb chromosomal position in the zebra finch. To identify genes that might be targets of selection responsible for the unusual geographic distribution of this region, we used Liftoff (https://github.com/agshumate/Liftoff) to transfer gene annotations from the zebra finch genome to the greenish warbler genome. We obtained a list of 20 genes in this region (see Supplementary Table S3 below). The gene with the most obvious possible function in the context of our study is MID2, which is mentioned as a candidate gene for migration in an unpublished thesis (https://oaktrust.library.tamu.edu/bitstream/handle/1969.1/196498/LENNON-FINALTHESIS-2022.pdf?sequence=1&isAllowed=y). Note that the disjunct distribution of this haplotype is associated with two forms found at similar latitudes to the east and west of the Tibetan plateau, and similar migration directions and distances. We also found that the gene BMP15 (bone morphogenetic protein 15) is located only 100kb upstream of the 4A LHBR region. This gene is an ancient paralog of BMP4, which has been identified as causal gene for morphological variation of beaks in Darwin's finches. Like migration distance, tarsus length also shows parallel clines (Irwin et al., 2001), and we speculate BMP could play a role in development.

Irwin, D. E., Bensch, S., & Price, T. D. (2001). Speciation in a ring. *Nature*, *409*(6818), 333–337
Takki, O., Komissarov, A., Kulak, M., & Galkina, S. (2022). Identification of centromere-specific repeats in the zebra finch genome. *Cytogenetic and Genome Research*, *162*(1–2), 55–63.

Supplementary Table S3. List of 20 genes inferred to be in the chromosome 4A LHBR.

Gene stable ID	Chromosome	Gene start (bp)	Gene end (bp)	Gene name	Transcript count	Gene description
ENSTGUG00000004925	4A	14,687,412	14,696,499	CLIC2	1	chloride intracellular channel protein 2 [Source:NCBI gene;Acc:100229375]
ENSTGUG00000004933	4A	14,709,358	14,715,954	FAAH2	1	fatty acid amide hydrolase 2 [Source:HGNC Symbol;Acc:HGNC:26440]
ENSTGUG00000004946	4A	14,813,234	14,875,666	**MID2**	3	probable E3 ubiquitin-protein ligase MID2 [Source:NCBI gene;Acc:100221705]
ENSTGUG00000004983	4A	14,935,592	14,937,427	PIN4	2	Taeniopygia guttata peptidylprolyl cis/trans isomerase, NIMA-interacting 4 (PIN4), mRNA. [Source:RefSeq mRNA;Acc:NM_001245417]
ENSTGUG00000004992	4A	14,938,745	14,943,026	ERCC6L	1	ERCC excision repair 6 like, spindle assembly checkpoint helicase [Source:NCBI gene;Acc:100222721]
ENSTGUG00000004995	4A	14,942,414	14,965,069	OCRL	4	OCRL inositol polyphosphate-5-phosphatase [Source:NCBI gene;Acc:100219842]
ENSTGUG00000005061	4A	14,972,756	14,979,144	XPNPEP2	1	X-prolyl aminopeptidase 2 [Source:HGNC Symbol;Acc:HGNC:12823]
ENSTGUG00000005127	4A	14,979,400	14,984,727	SASH3	1	SAM and SH3 domain containing 3 [Source:HGNC Symbol;Acc:HGNC:15975]
ENSTGUG00000005130	4A	14,998,889	15,005,639		1	
ENSTGUG00000005167	4A	15,041,733	15,056,798	AIFM1	3	apoptosis inducing factor mitochondria associated 1 [Source:NCBI gene;Acc:100226565]
ENSTGUG00000005225	4A	15,055,908	15,061,374	MARS2	1	methionyl-tRNA synthetase 2, mitochondrial [Source:HGNC Symbol;Acc:HGNC:25133]
ENSTGUG00000005228	4A	15,061,911	15,065,231	RAB33A	1	RAB33A, member RAS oncogene family [Source:NCBI gene;Acc:100221653]
ENSTGUG00000005239	4A	15,065,522	15,067,345		1	
ENSTGUG00000005241	4A	15,068,031	15,075,171	SLC25A14	1	solute carrier family 25 member 14 [Source:NCBI gene;Acc:100231274]
ENSTGUG00000018290	4A	15,077,318	15,078,926	GPR119	1	G protein-coupled receptor 119 [Source:HGNC Symbol;Acc:HGNC:19060]
ENSTGUG00000019242	4A	14,753,447	14,770,954	TSC22D3	3	TSC22 domain family member 3 [Source:NCBI gene;Acc:100227519]
ENSTGUG00000020838	4A	15,021,673	15,038,088	BCORL1	2	BCL6 corepressor like 1 [Source:NCBI gene;Acc:100217816]
ENSTGUG00000020851	4A	14,917,101	14,934,705	NHSL2	2	NHS like 2 [Source:NCBI gene;Acc:100231328]
ENSTGUG00000021007	4A	14,968,207	14,969,195		1	
ENSTGUG00000025832	4A	14,984,652	14,996,721	ZDHHC9	1	zinc finger DHHC-type containing 9 [Source:NCBI gene;Acc:115495256]

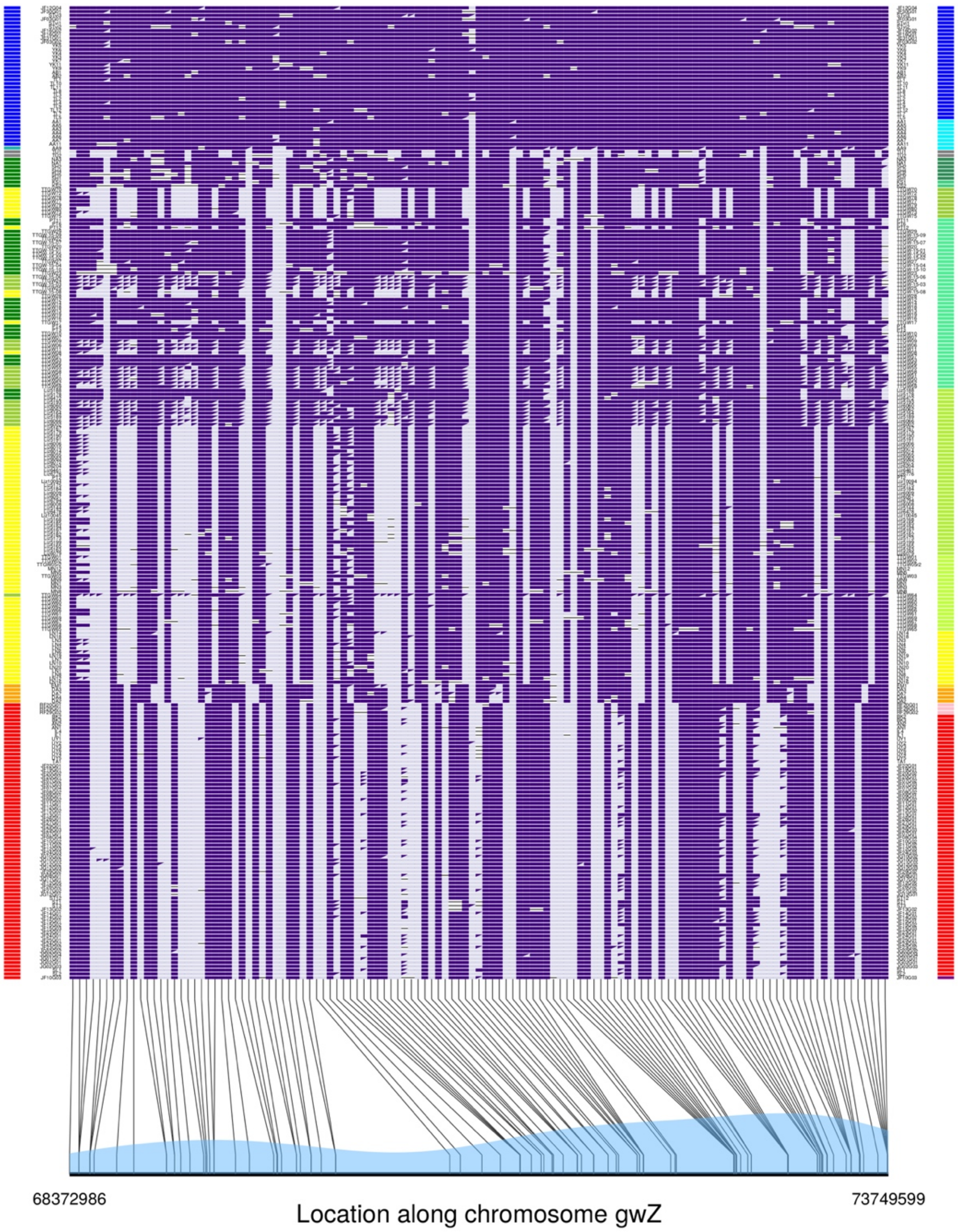

Figure S1. SNP genotypes within the Z chromosome LHBR, for all individuals in the study. Individuals are in rows and SNPs are in columns. Only those SNPs that are highly differentiated are shown (see Methods). Colours on the left side indicate the PCA clusters, and colours on the right side indicate sampling site. Individual identification codes are near those colored boxes. This figure is similar in format to Figure 4, but here all individuals are shown and they are arranged in order of their sampling sites around the ring. At the bottom of the figure, a line represents the length of the chromosome Z LHBR (spanning nucleotides 68,372,986 and 73,749,599 in the reference genome), with locations of illustrated SNPs indicated (with the pale blue curve representing the density distribution of all SNPs).

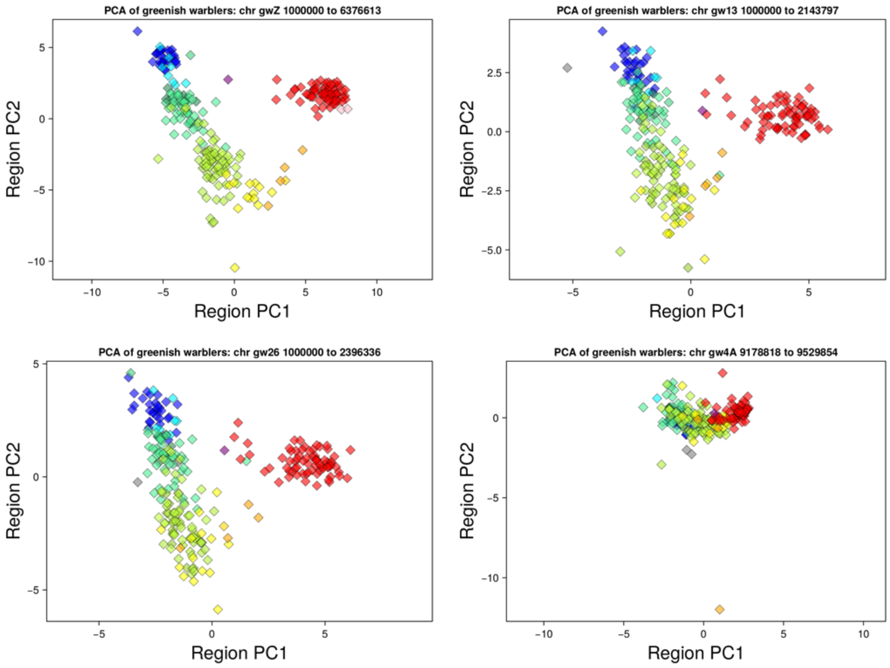

Figure S2. PCAs of variation in non-LHBR parts of chromosome Z (top left), chromosome 13 (top right), chromosome 26 (bottom left), and chromosome 4A (lower right). Each is based on an arbitrary non-LHBR part of the same length as the LHBR on that chromosome. The arbitrary location was chosen as being 1 Mb from the left or right end of the chromosome, far from the LHBR.

Figures S3-S32 (pages 9-38). Genotype-by-individual plots for greenish warbler scaffolds corresponding to chromosomes Z, 1, 1A, 2-4, 4A, 5-15, and 17-28. Each row represents an individual (257 total), with geographic location / subspecies indicated with colored boxes on left and right (see Fig. 1A). Individual identification codes are near those colored boxes. The *viridanus* / *plumbeitarsus* hybrid backcross (JF10G03) is in the lowest row. Columns represent highly differentiated SNPs ($F_{ST} > 0.9$ for the Z chromosome, $F_{ST} > 0.8$ for all others, among any pairs of these three sample sets: west Siberian *viridanus*, *trochiloides* from Nepal, and east Siberian *plumbeitarsus*), with dark and light purple representing two alleles, with the dark purple allele have a frequency greater than 50% in *viridanus*. Homozygous genotypes are solid rectangles, whereas heterozygous genotypes are represented by dividing that rectangle into two triangles. Missing genotypes are indicated with a black line through white space. The lower part of the figure shows where each illustrated SNP is located on the scaffold (with the pale blue curve representing the density distribution of all SNPs), and the locations of large haploblock regions (LHBRs) is indicated by magenta bars along the lower line representing each scaffold.

Figure S3:

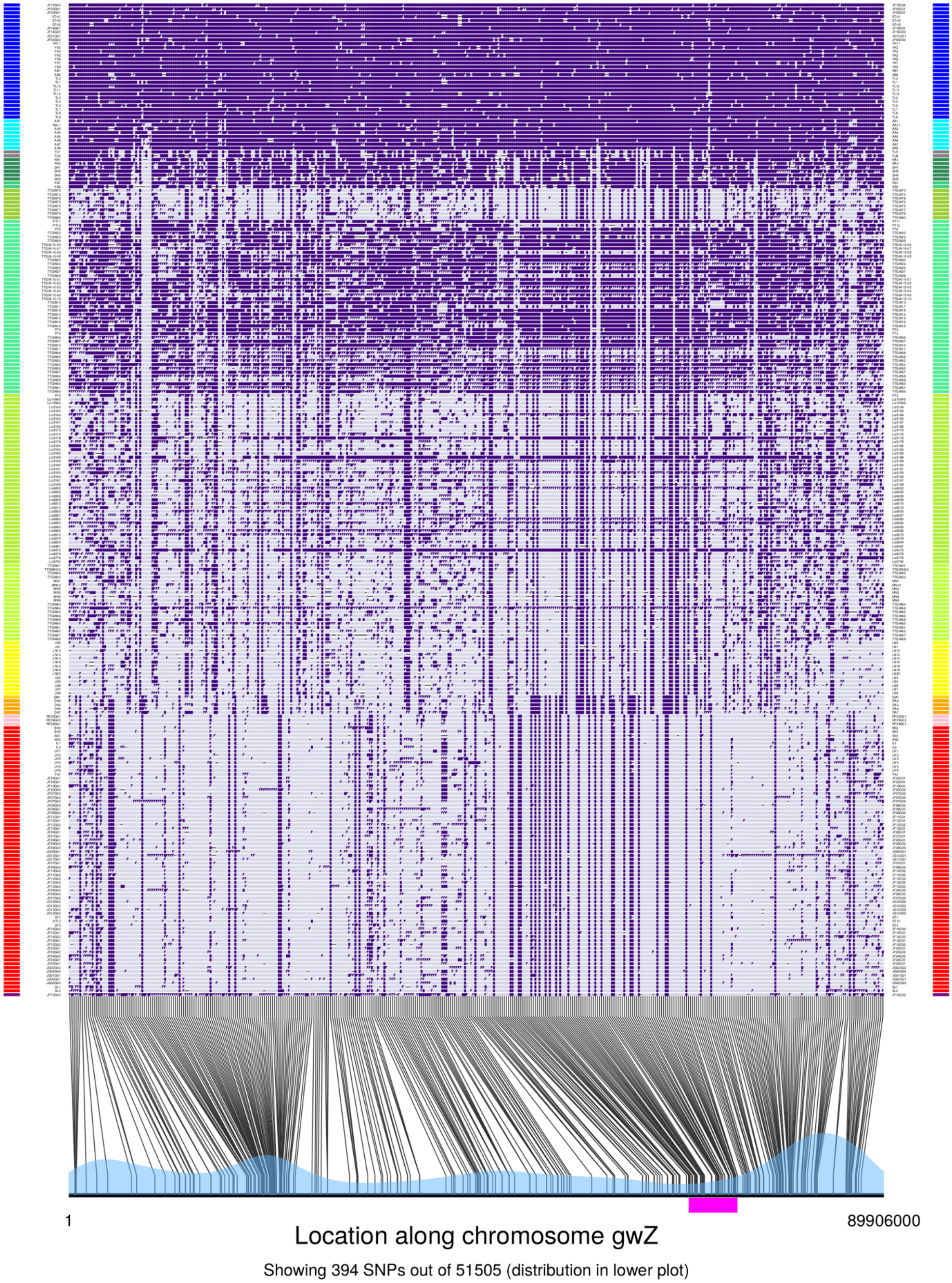
1
89906000
Location along chromosome gwZ
Showing 394 SNPs out of 51505 (distribution in lower plot)

Figure S4:

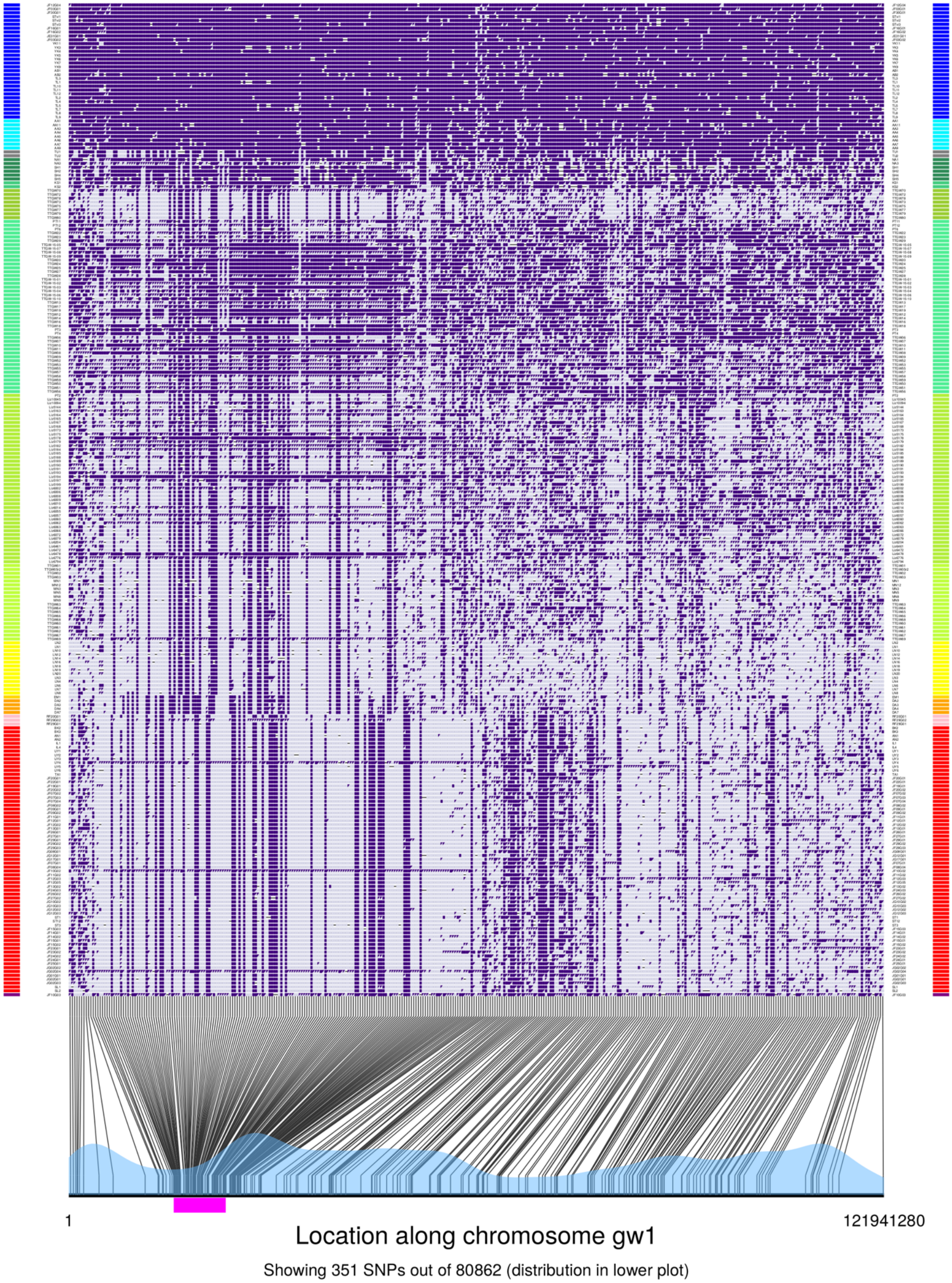
1
121941280
Location along chromosome gw1
Showing 351 SNPs out of 80862 (distribution in lower plot)

Figure S5:

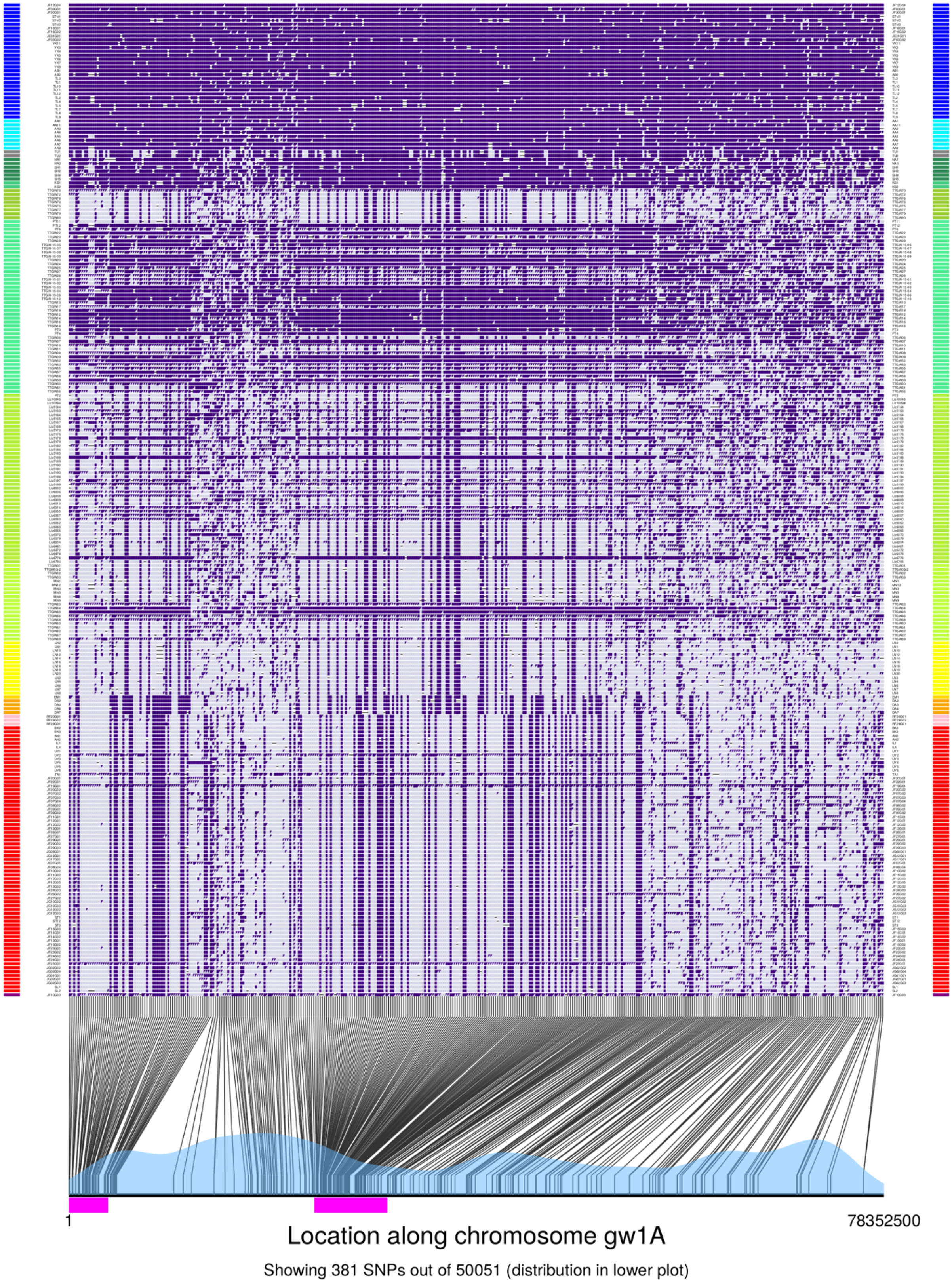
1
78352500
Location along chromosome gw1A
Showing 381 SNPs out of 50051 (distribution in lower plot)

Figure S6:

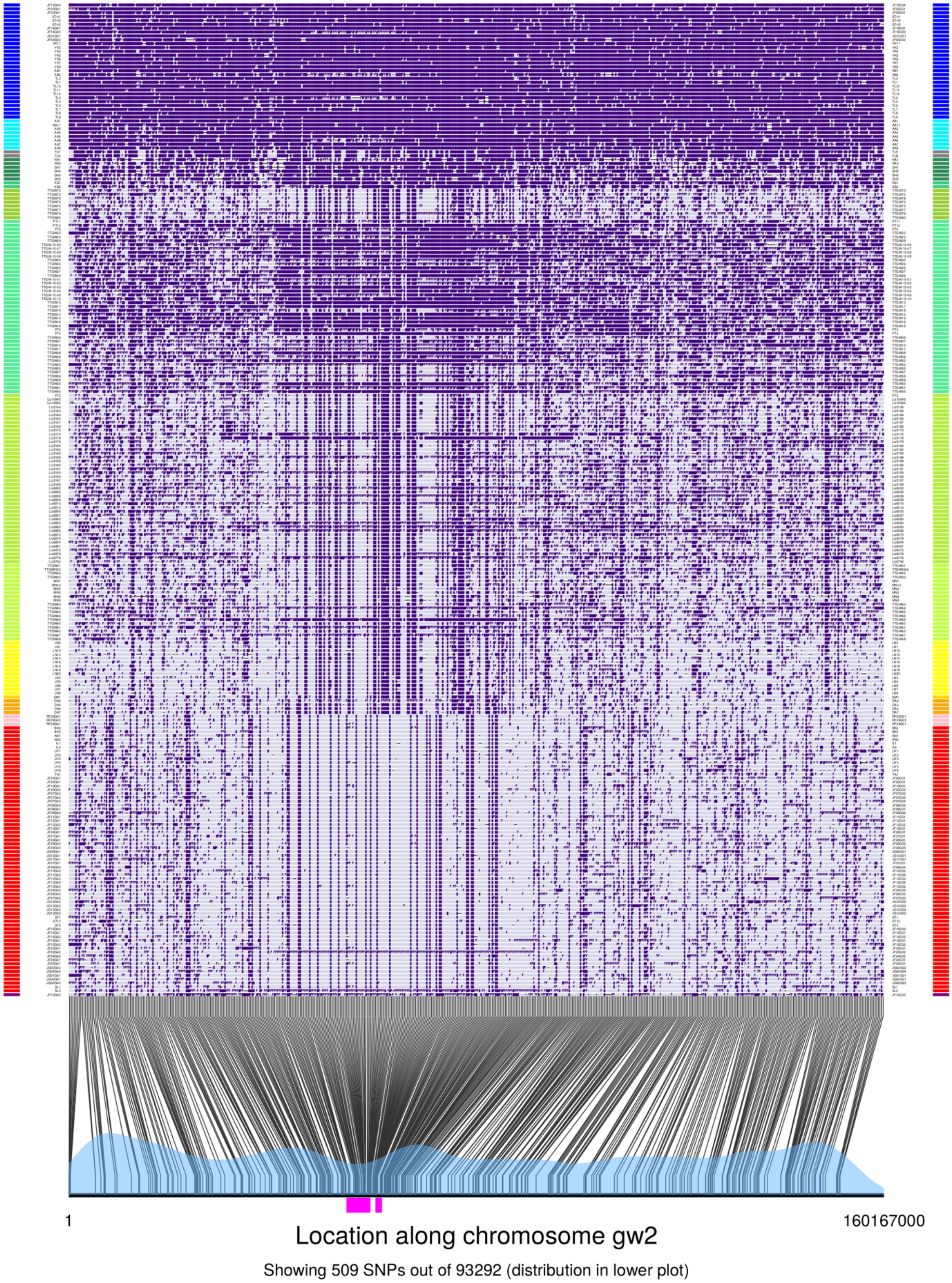
1
160167000
Location along chromosome gw2
Showing 509 SNPs out of 93292 (distribution in lower plot)

Figure S7:

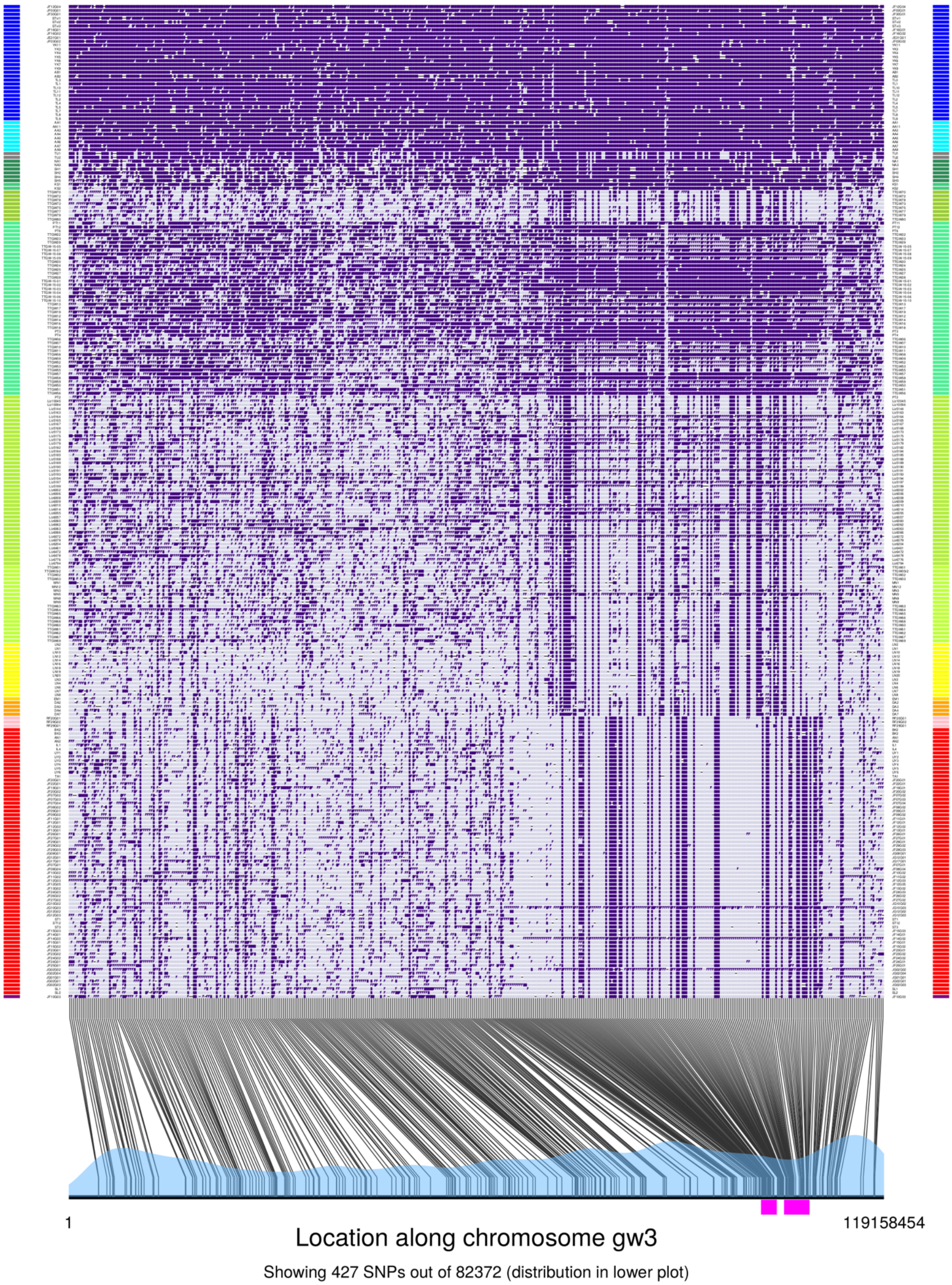
1
119158454
Location along chromosome gw3
Showing 427 SNPs out of 82372 (distribution in lower plot)

Figure S8:

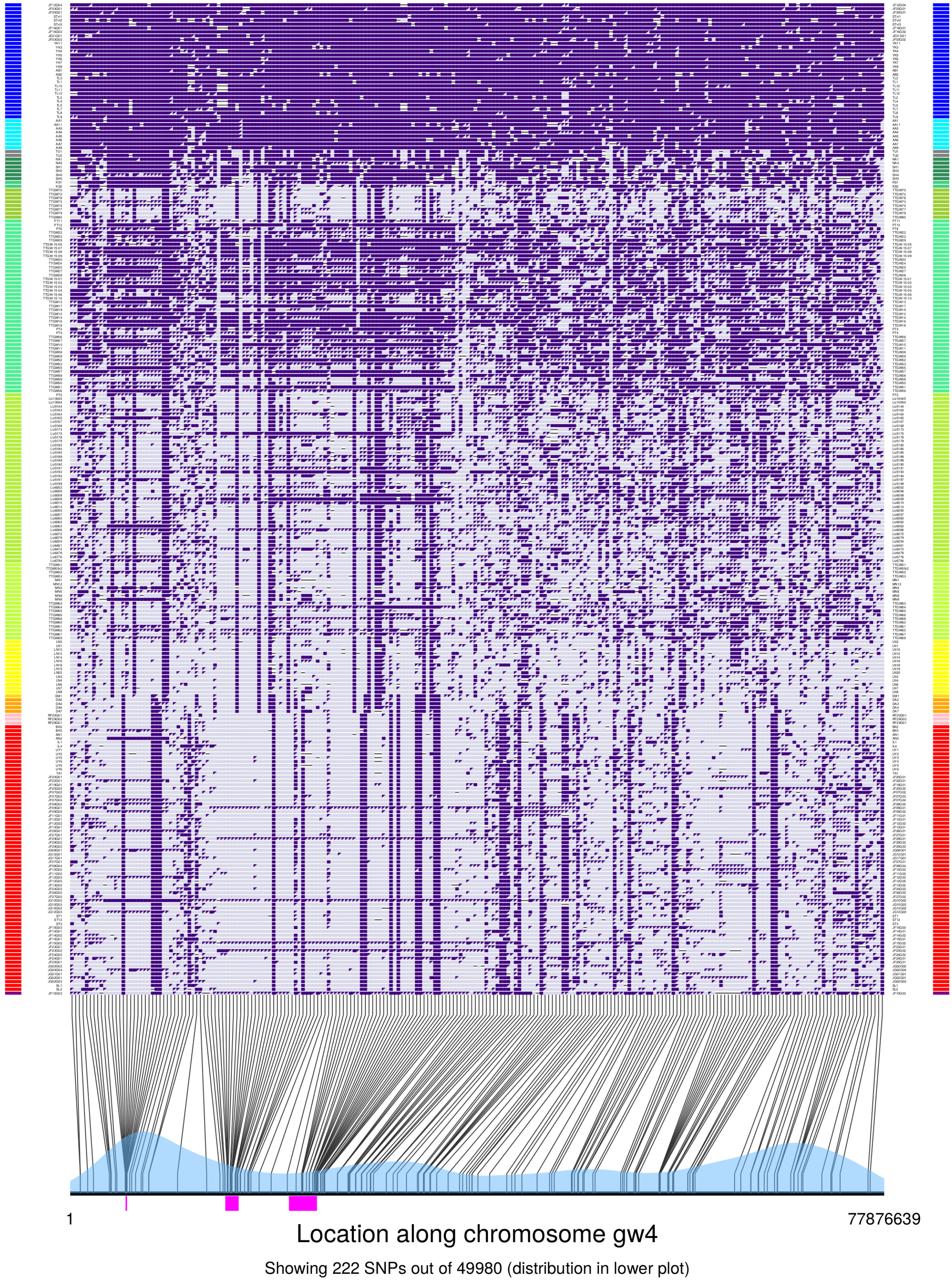
1
77876639
Location along chromosome gw4
Showing 222 SNPs out of 49980 (distribution in lower plot)

Figure S9:

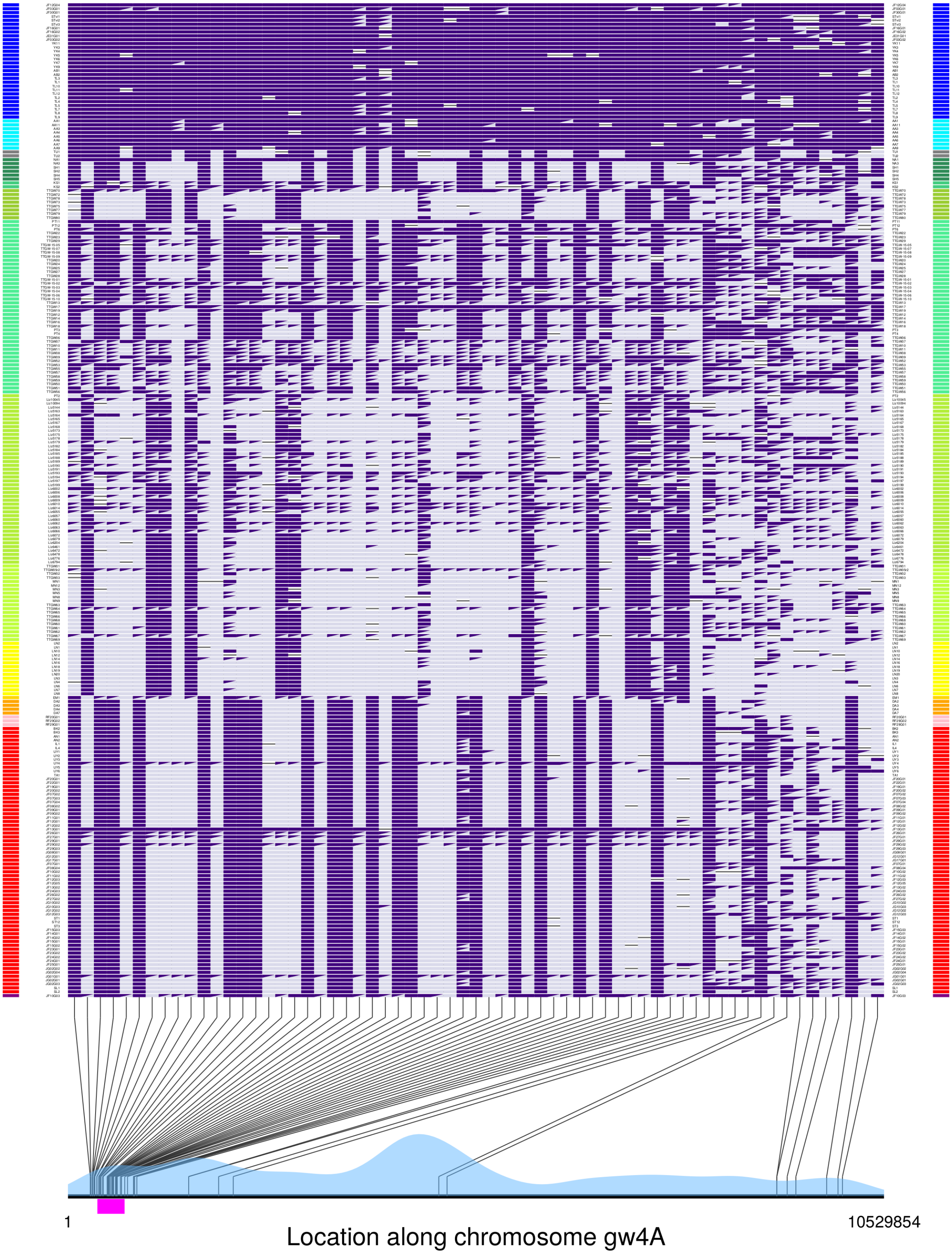

Showing 63 SNPs out of 18467 (distribution in lower plot)

Figure S10:

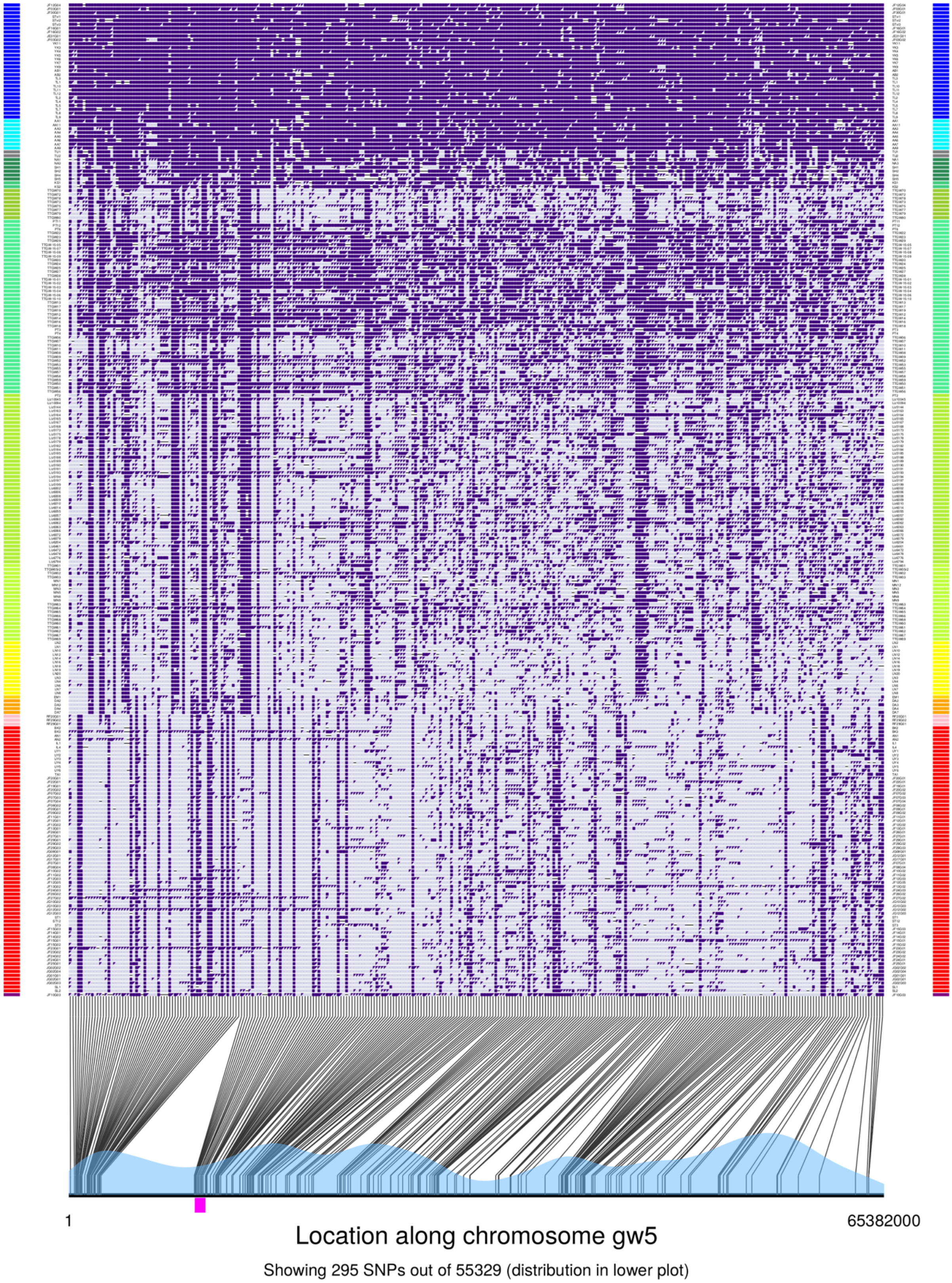
1
65382000
Location along chromosome gw5
Showing 295 SNPs out of 55329 (distribution in lower plot)

Figure S11:

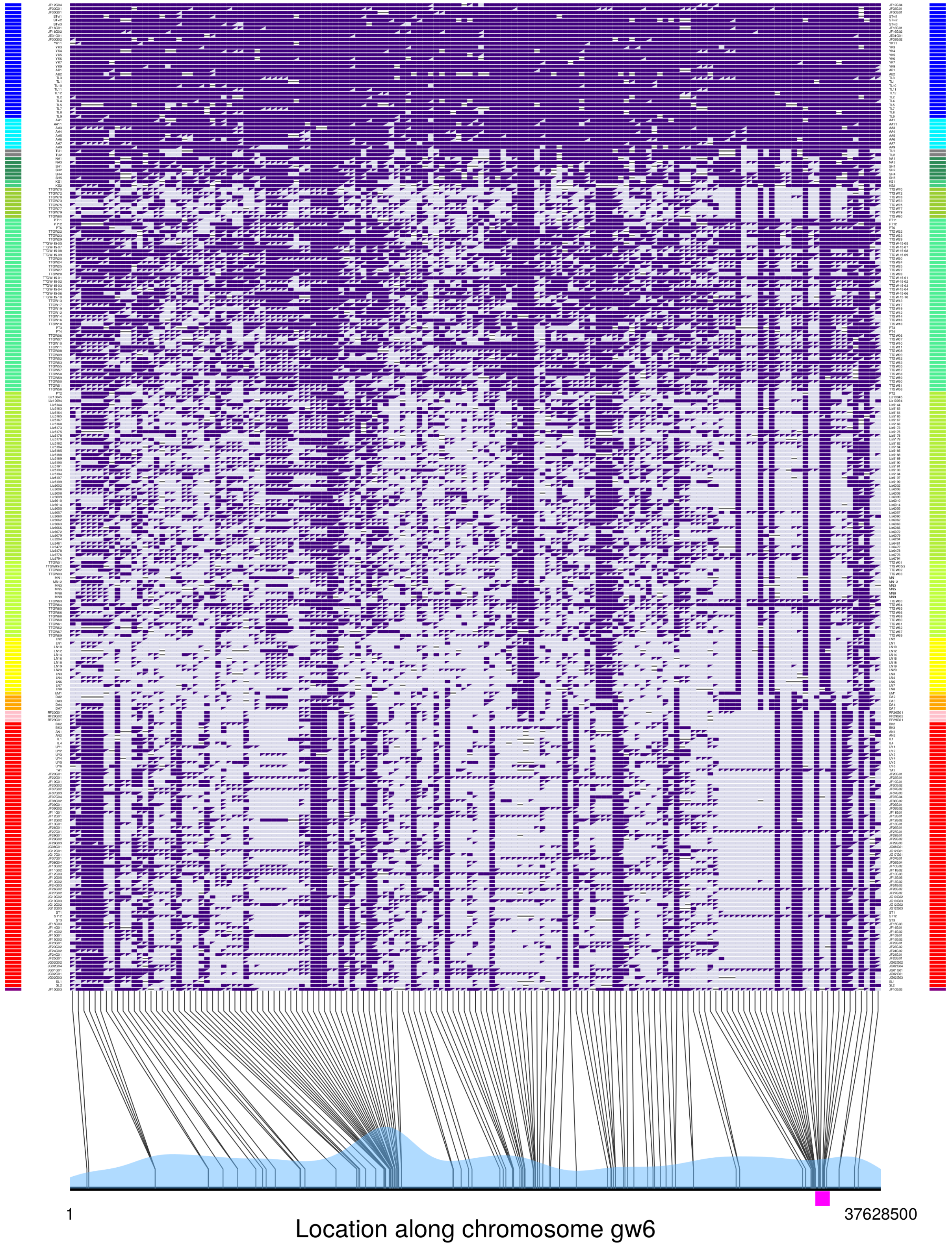

Showing 145 SNPs out of 40175 (distribution in lower plot)

Figure S12:

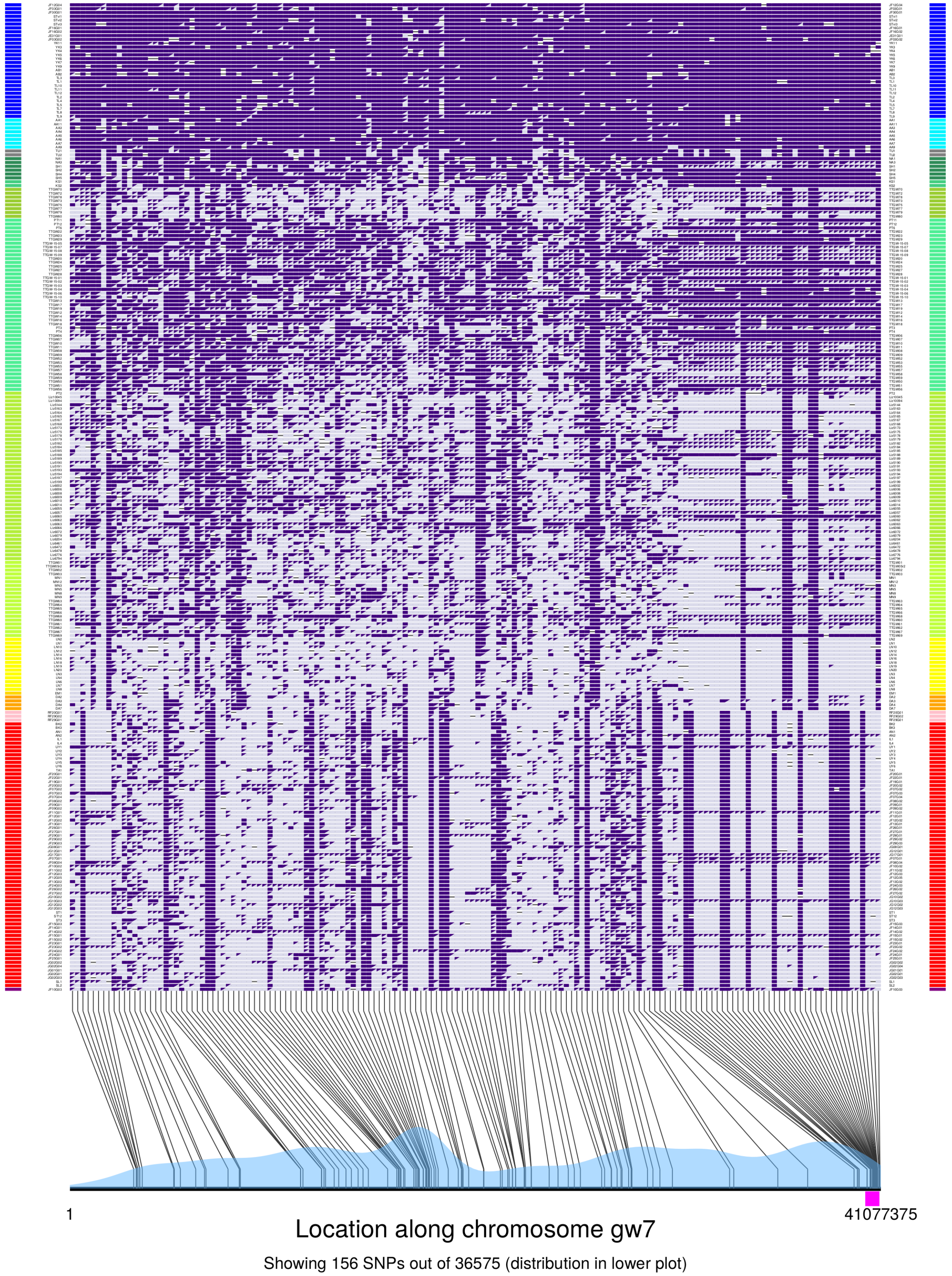
1
41077375
Location along chromosome gw7
Showing 156 SNPs out of 36575 (distribution in lower plot)

Figure S13:

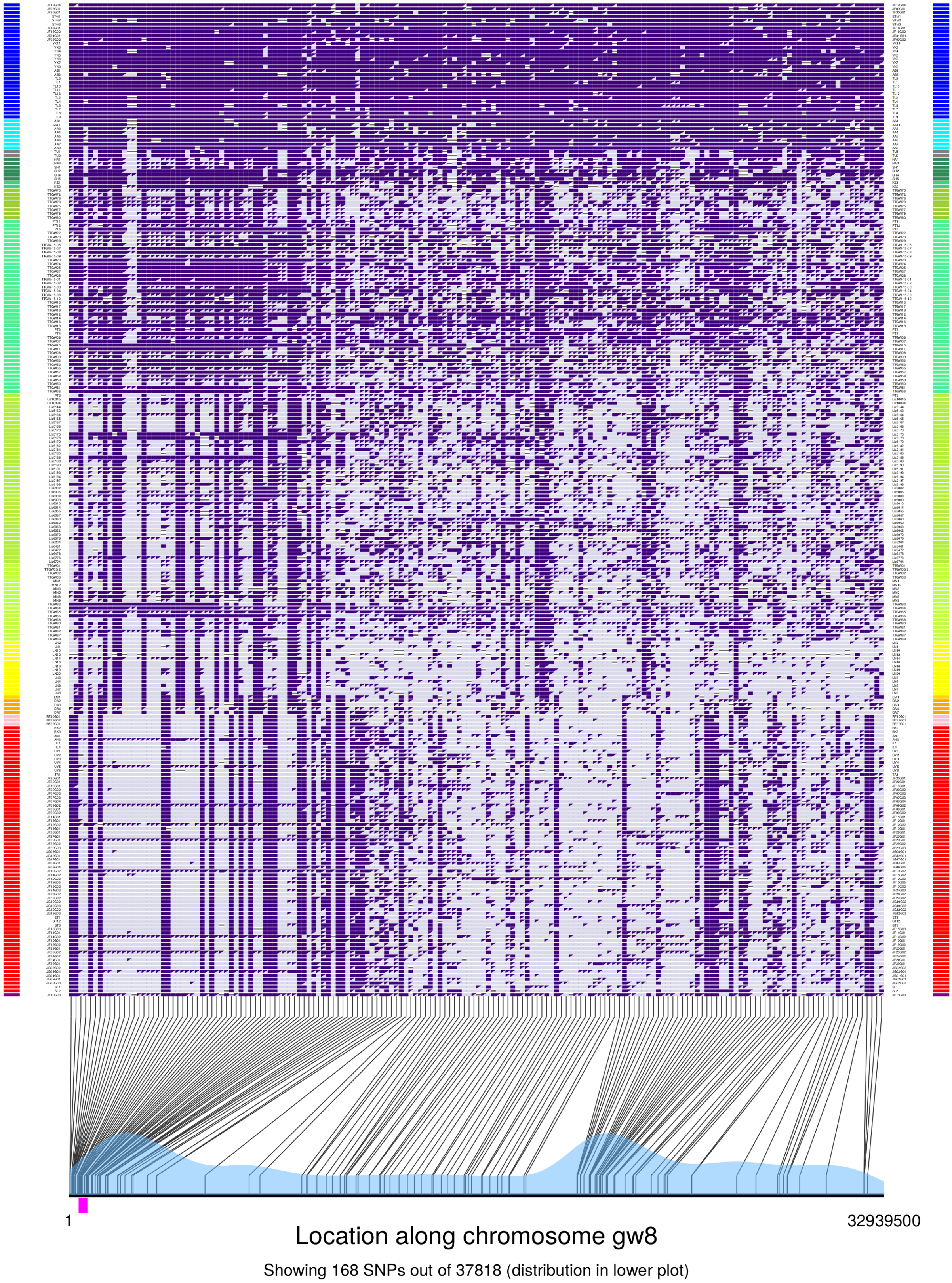
1
32939500
Location along chromosome gw8
Showing 168 SNPs out of 37818 (distribution in lower plot)

Figure S14:

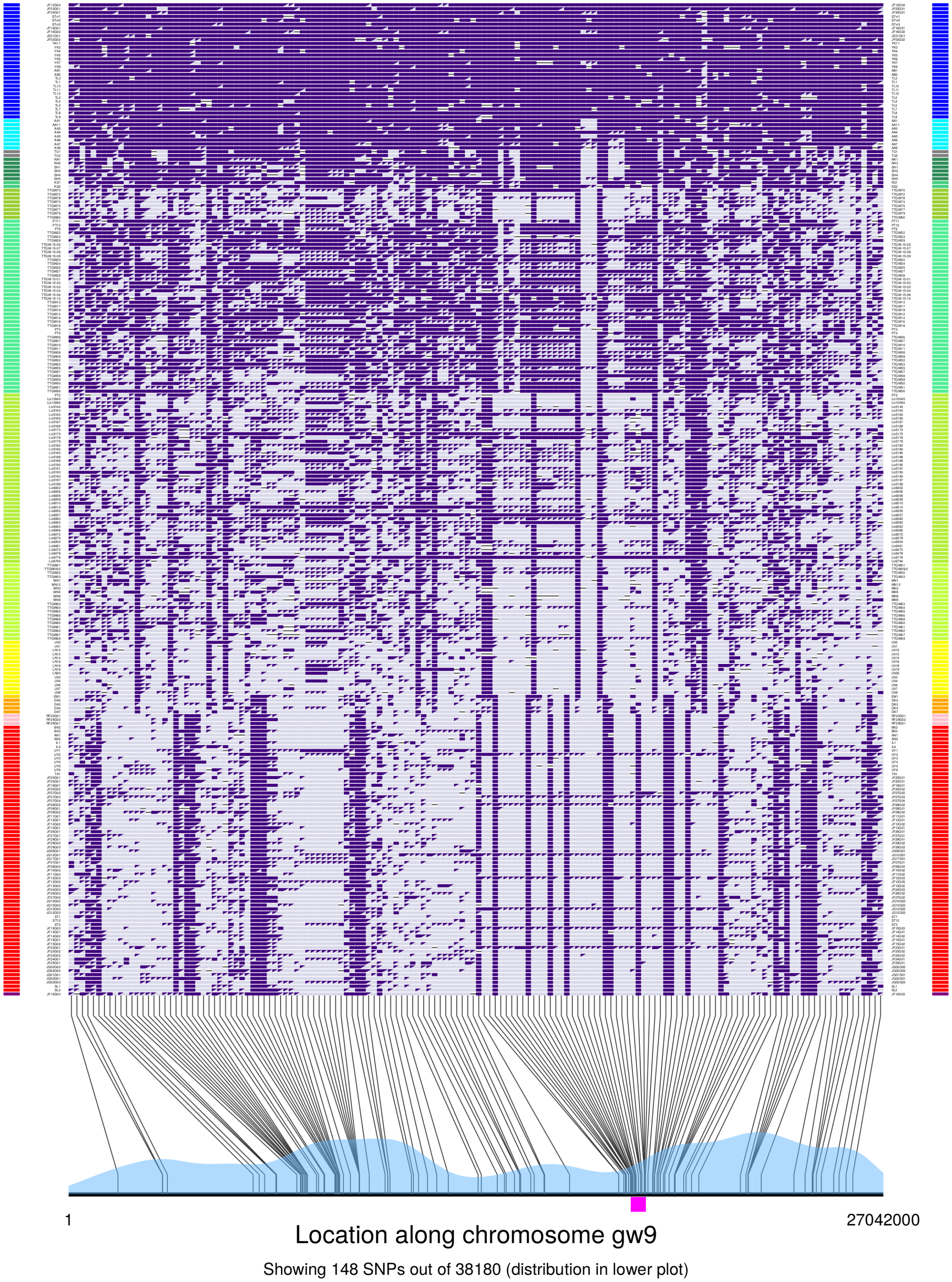
1
27042000
Location along chromosome gw9
Showing 148 SNPs out of 38180 (distribution in lower plot)

Figure S15:

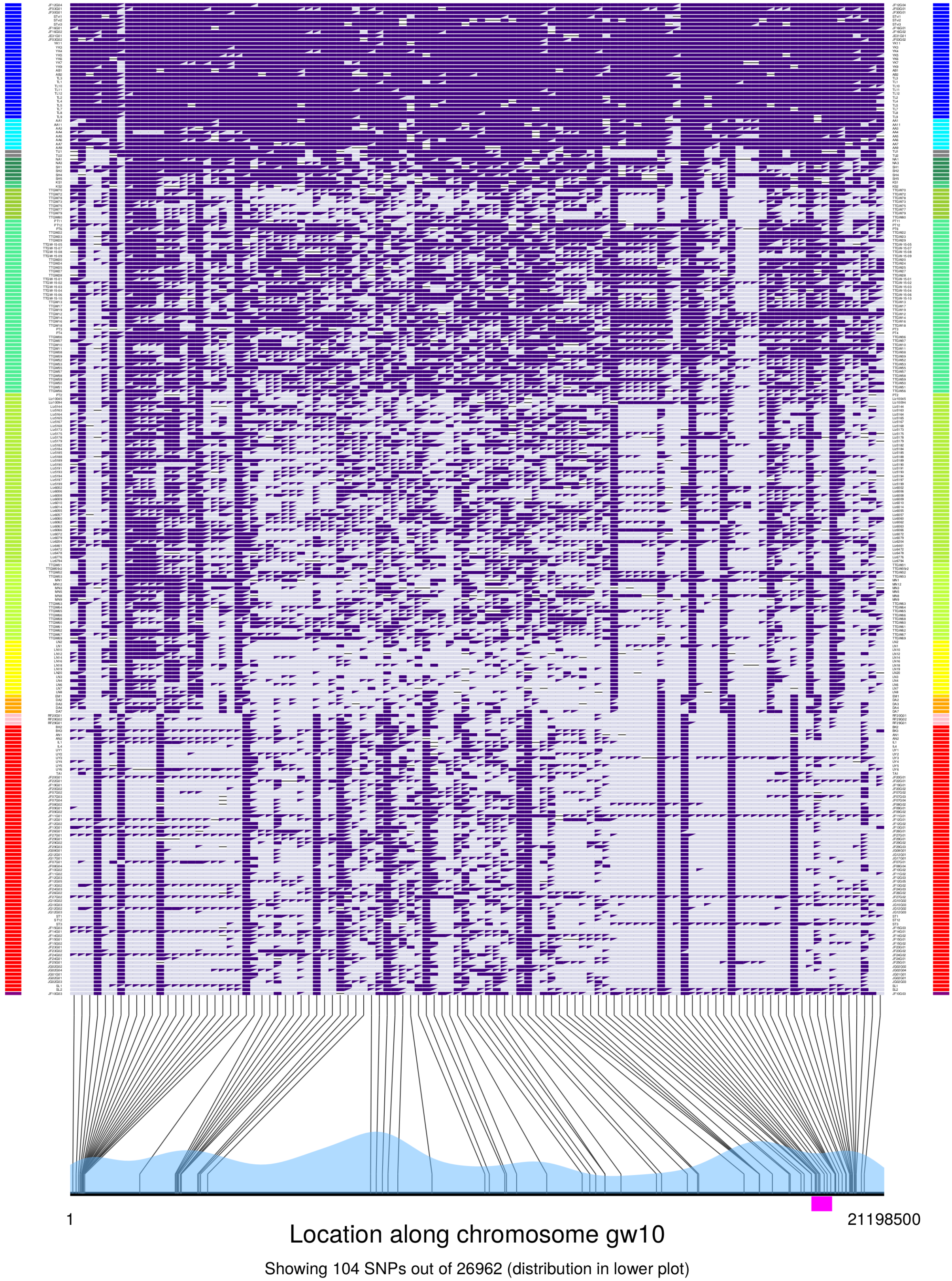
1
21198500
Location along chromosome gw10
Showing 104 SNPs out of 26962 (distribution in lower plot)

Figure S16:

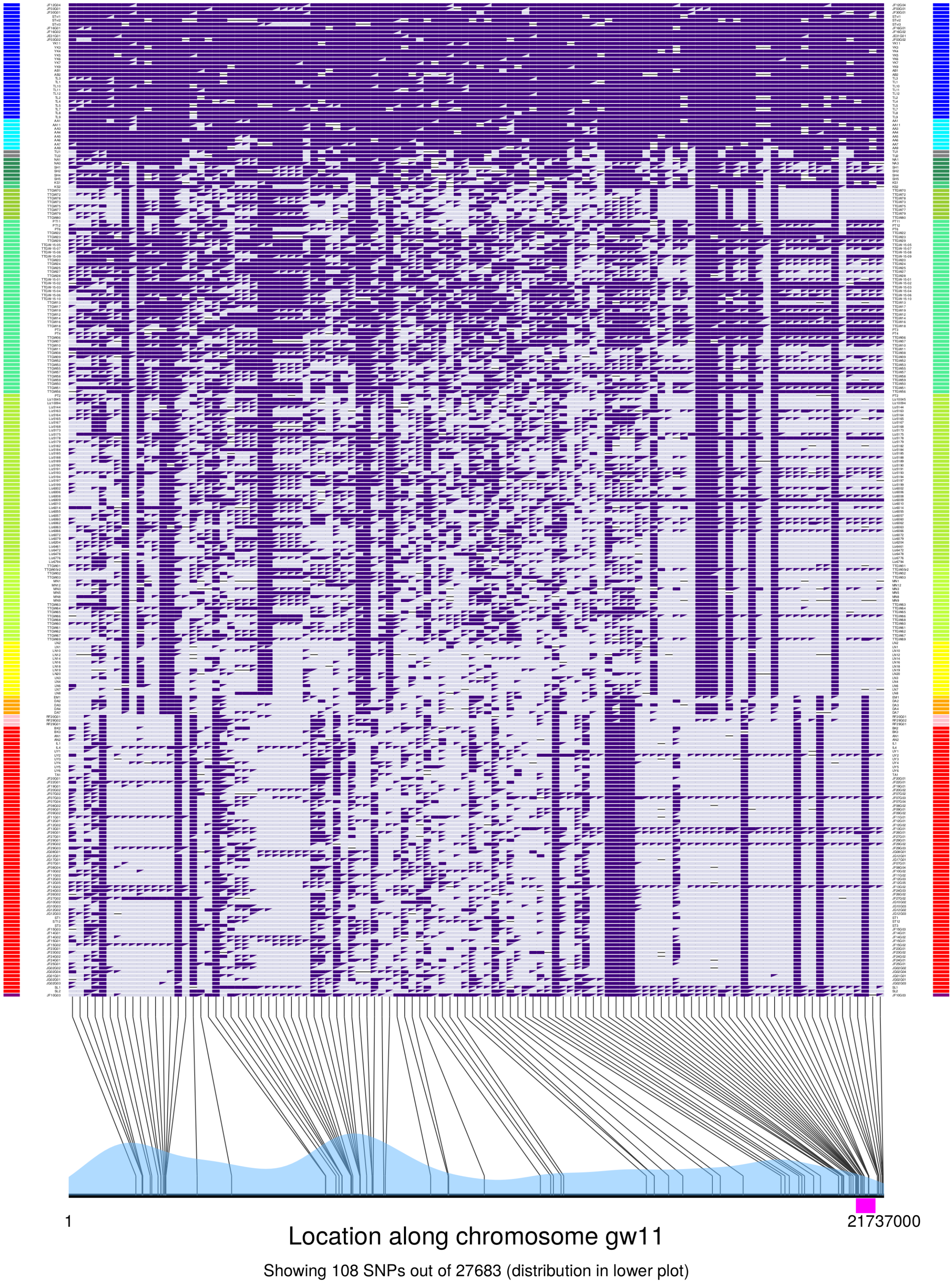
1
21737000
Location along chromosome gw11
Showing 108 SNPs out of 27683 (distribution in lower plot)

Figure S17:

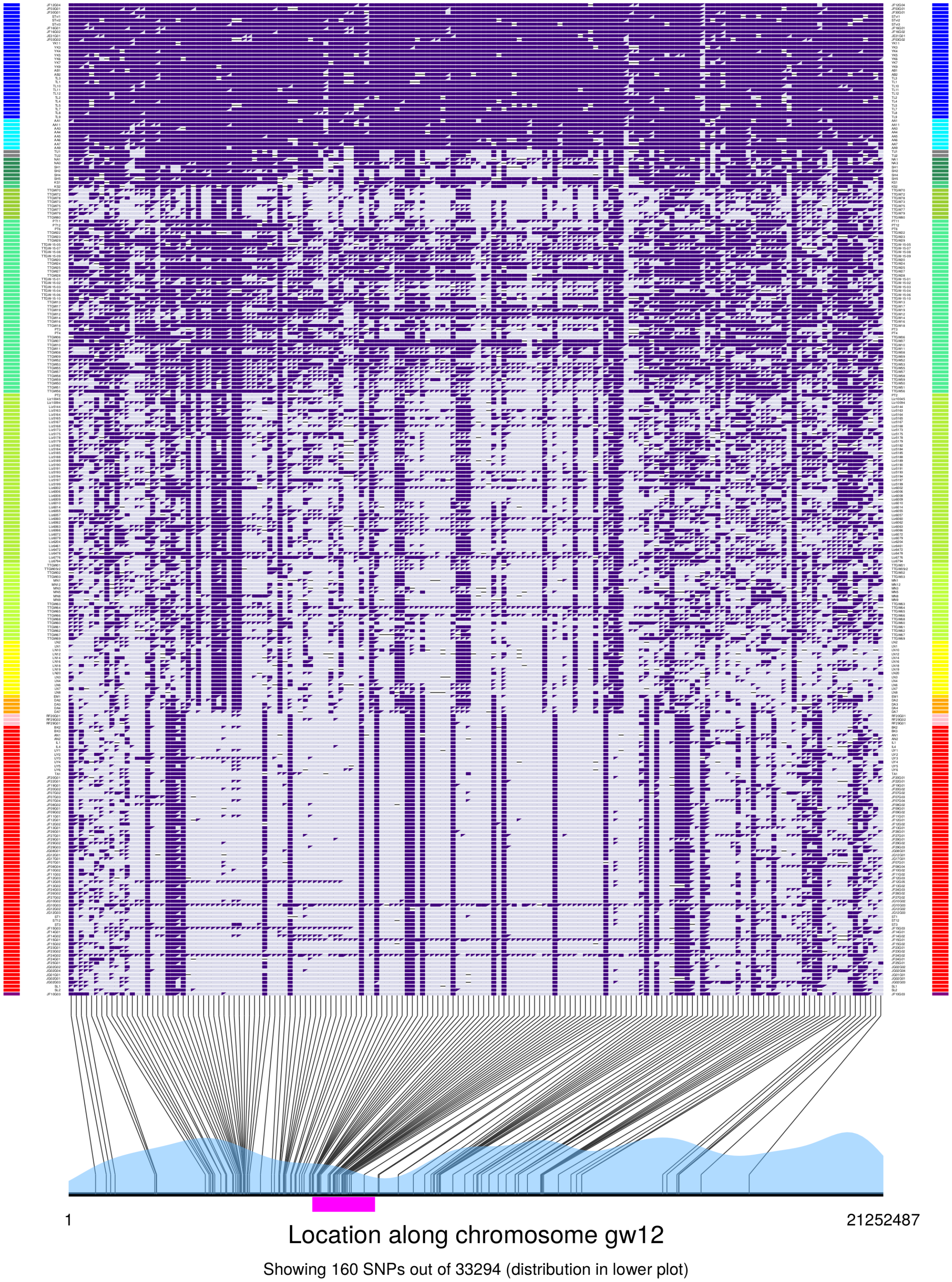
1
21252487
Location along chromosome gw12
Showing 160 SNPs out of 33294 (distribution in lower plot)

Figure S18:

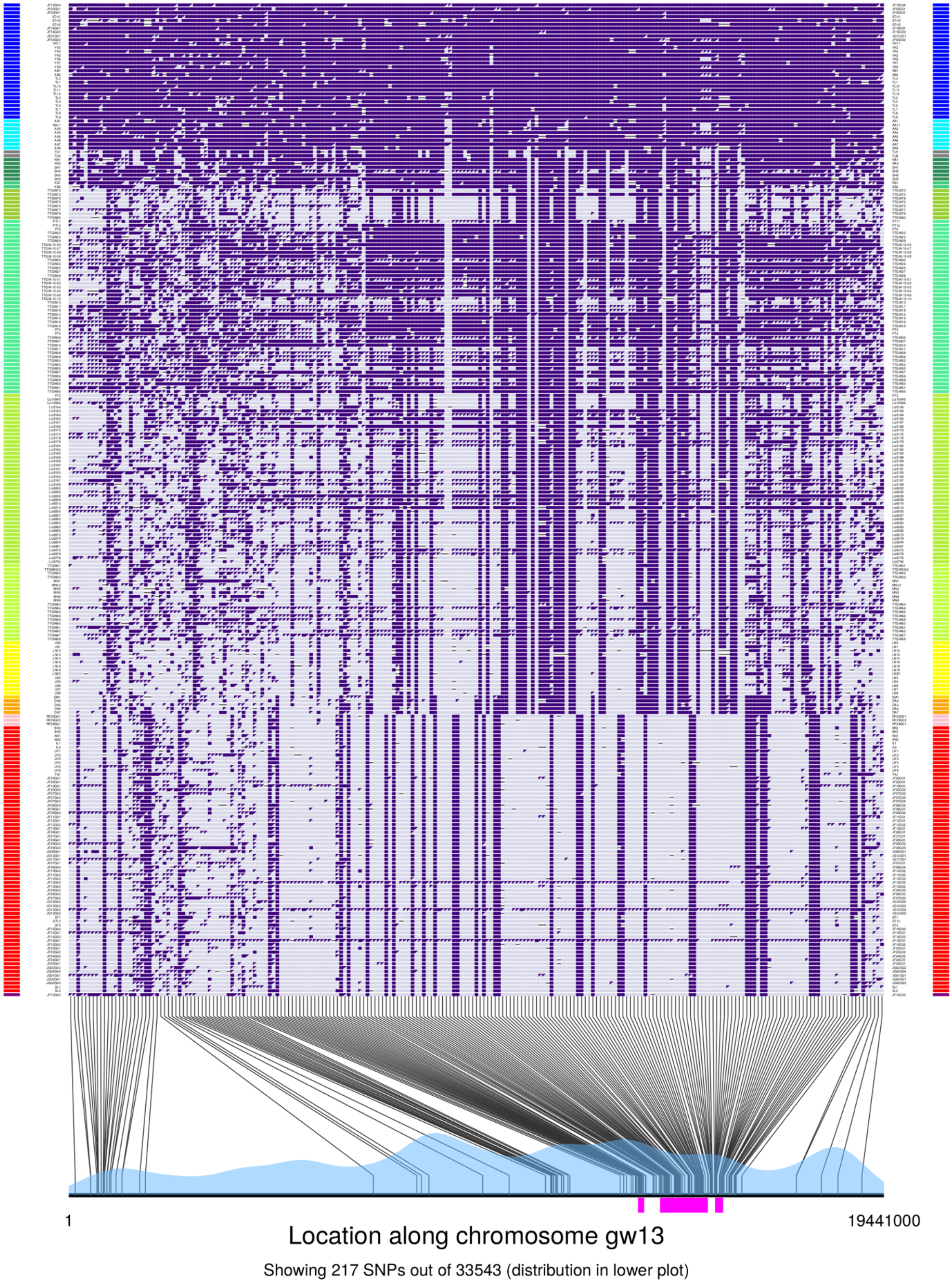
1
19441000
Location along chromosome gw13
Showing 217 SNPs out of 33543 (distribution in lower plot)

Figure S19:

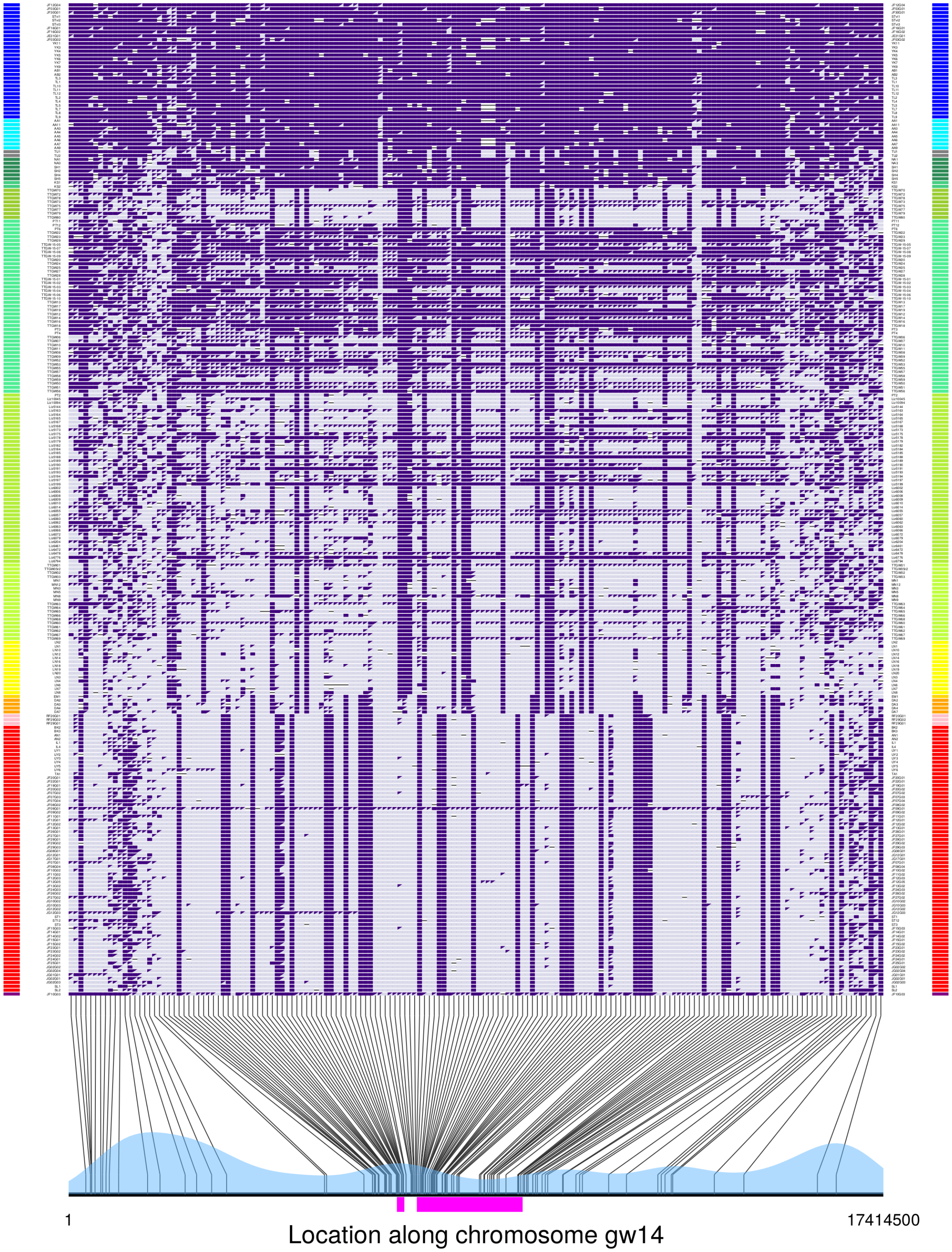

Showing 166 SNPs out of 30969 (distribution in lower plot)

Figure S20:

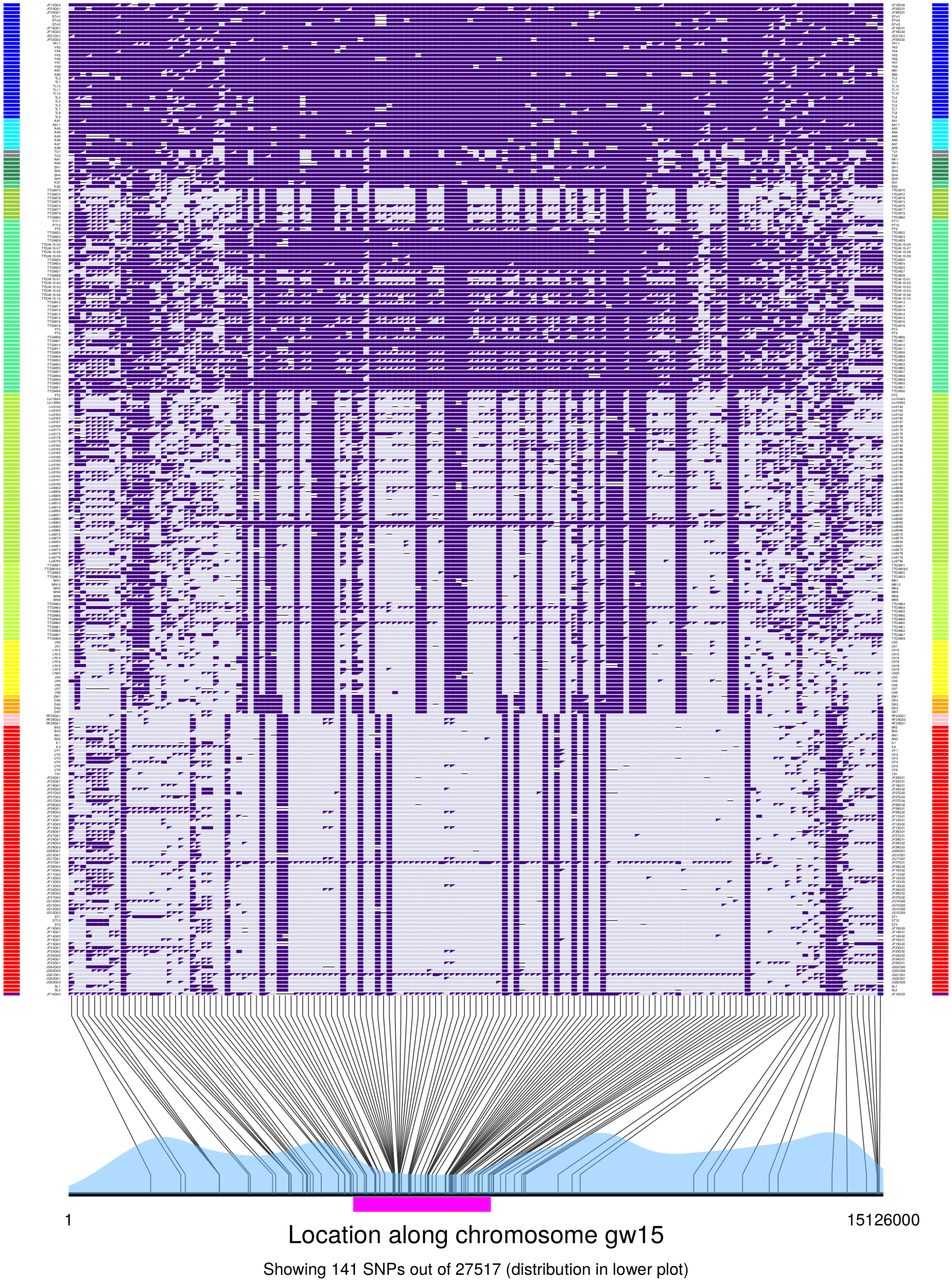
1
15126000
Location along chromosome gw15
Showing 141 SNPs out of 27517 (distribution in lower plot)

Figure S21:

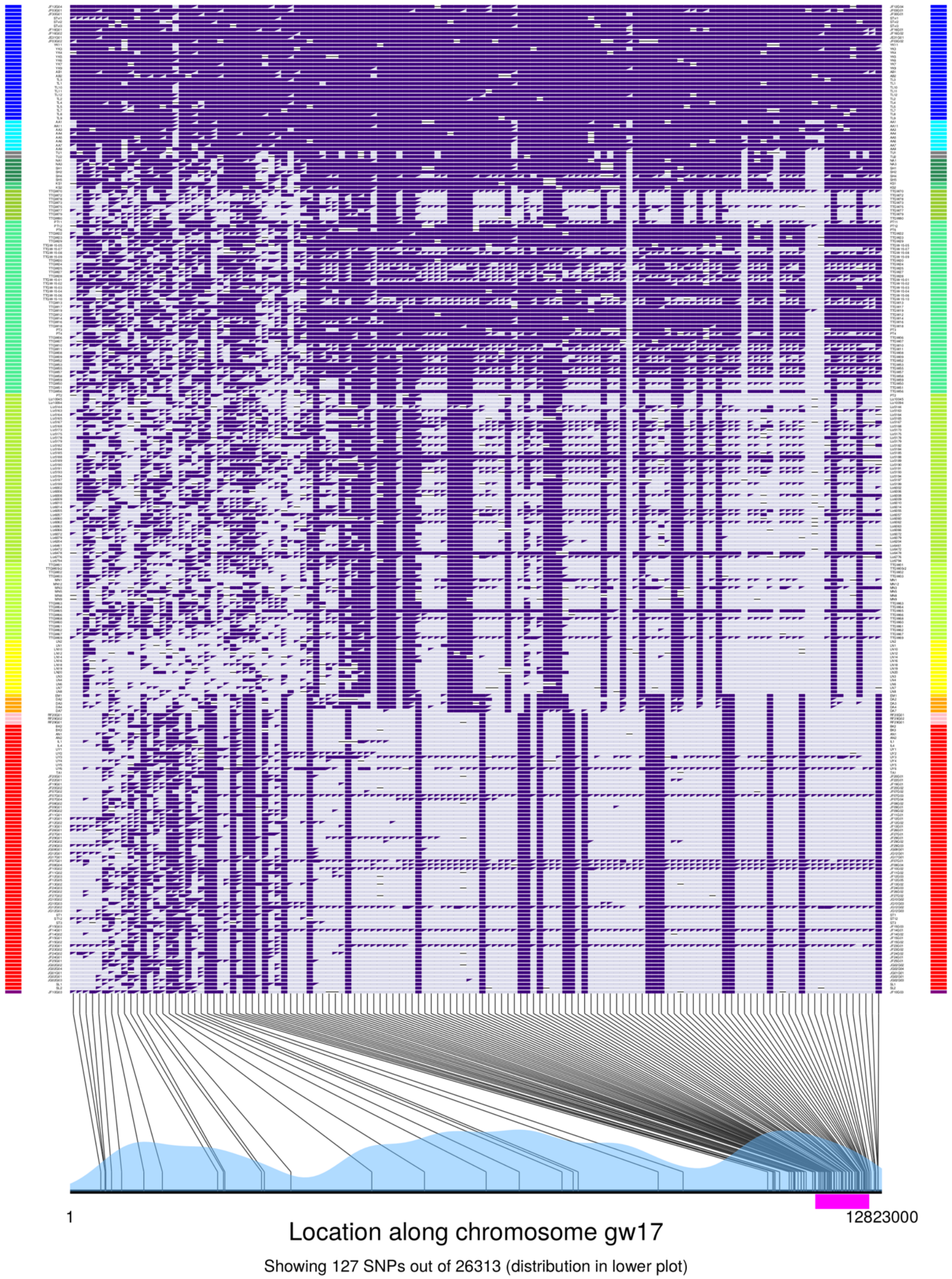
1
12823000
Location along chromosome gw17
Showing 127 SNPs out of 26313 (distribution in lower plot)

Figure S22:

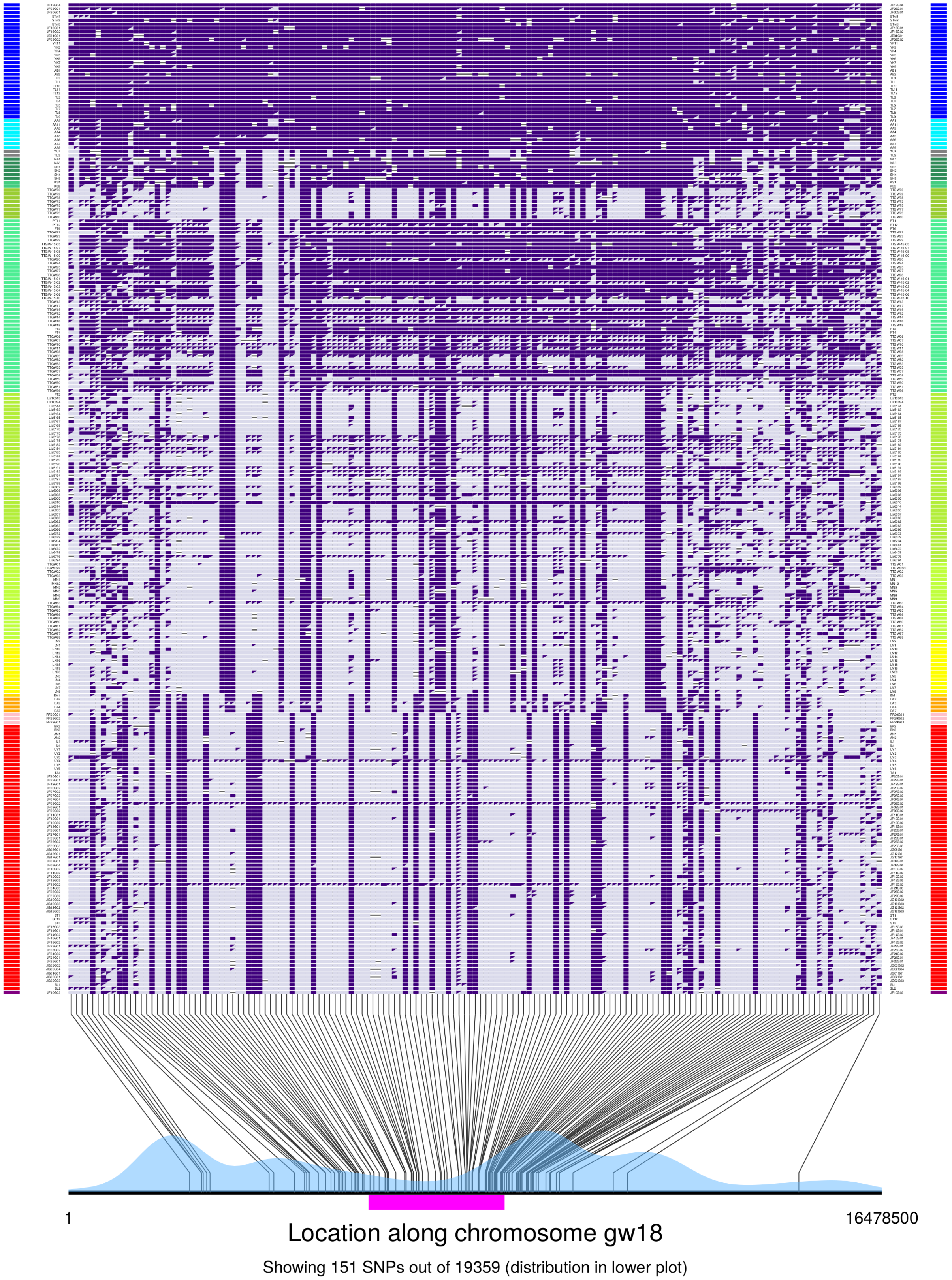
1
16478500
Location along chromosome gw18
Showing 151 SNPs out of 19359 (distribution in lower plot)

Figure S23:

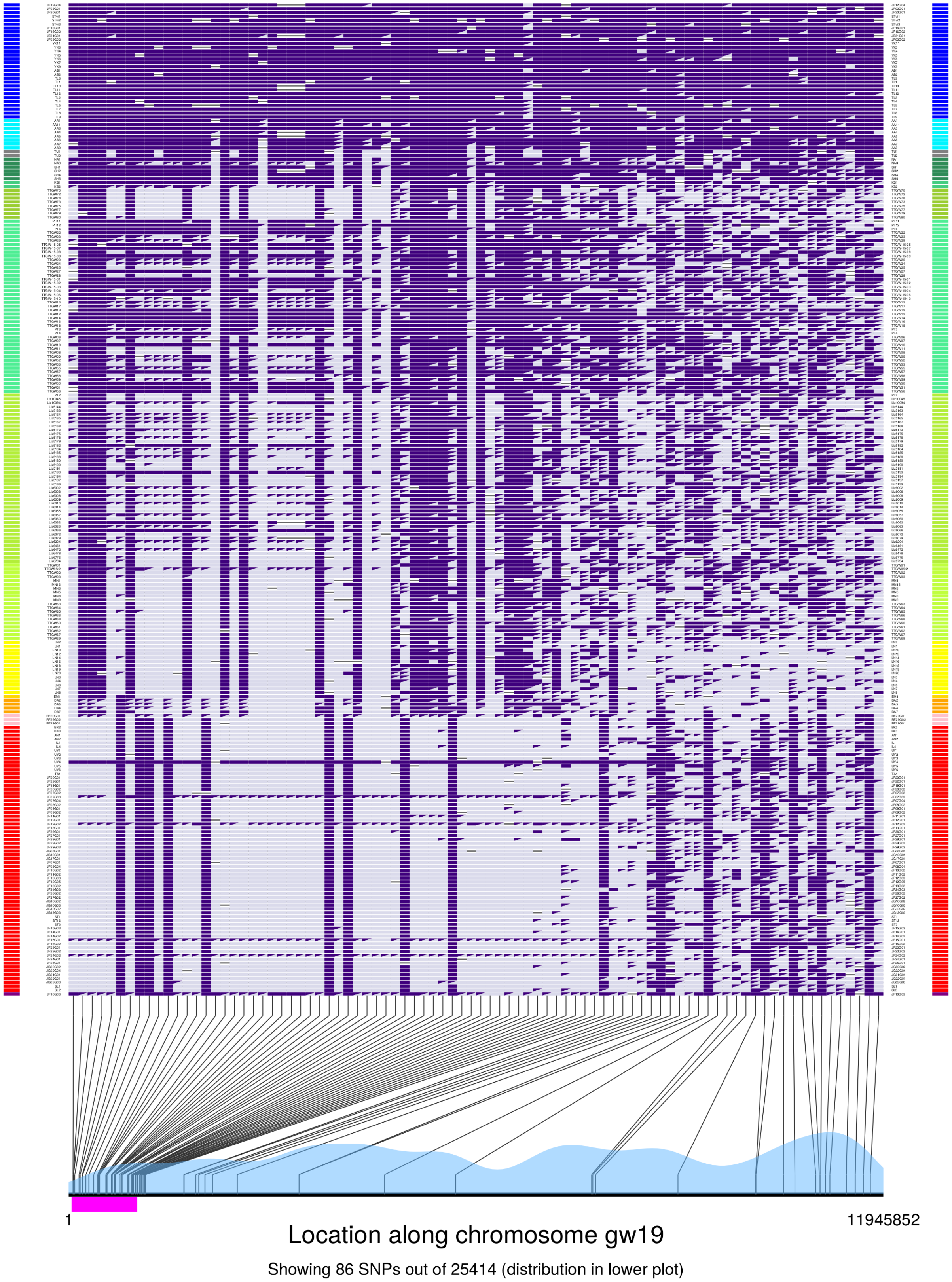

1
11945852
Location along chromosome gw19
Showing 86 SNPs out of 25414 (distribution in lower plot)

Figure S24:

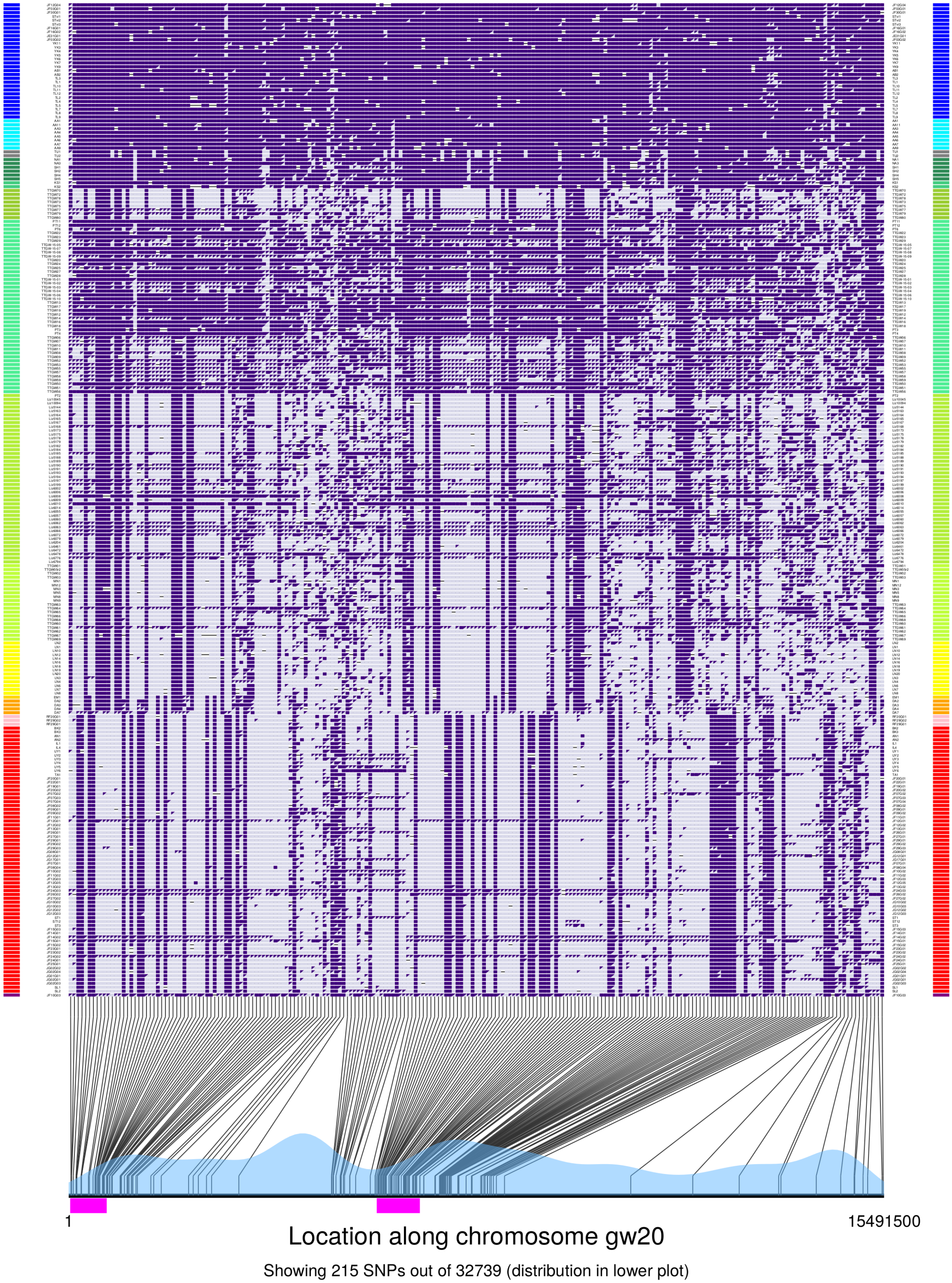
1
15491500
Location along chromosome gw20
Showing 215 SNPs out of 32739 (distribution in lower plot)

Figure S25:

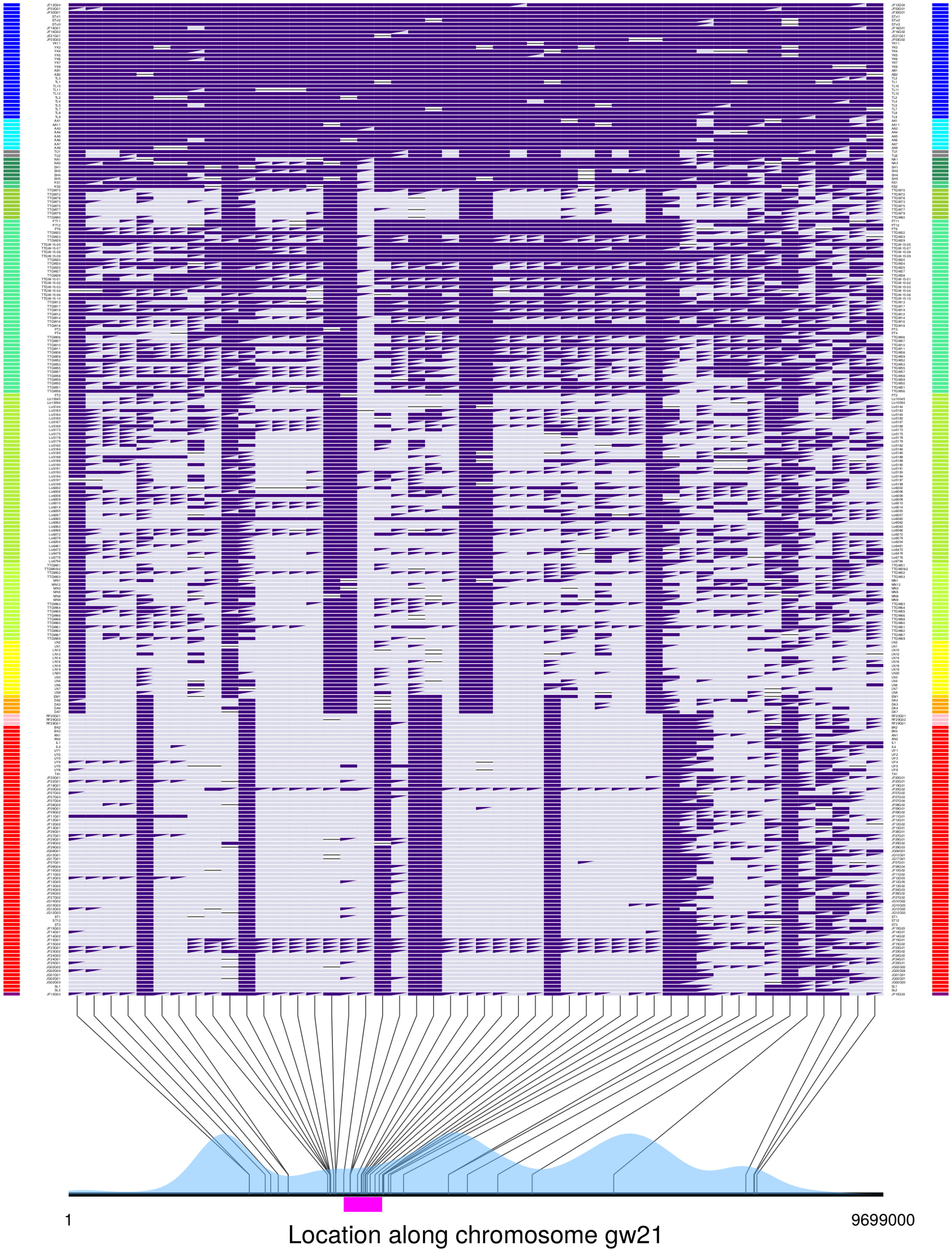

Showing 48 SNPs out of 13321 (distribution in lower plot)

Figure S26:

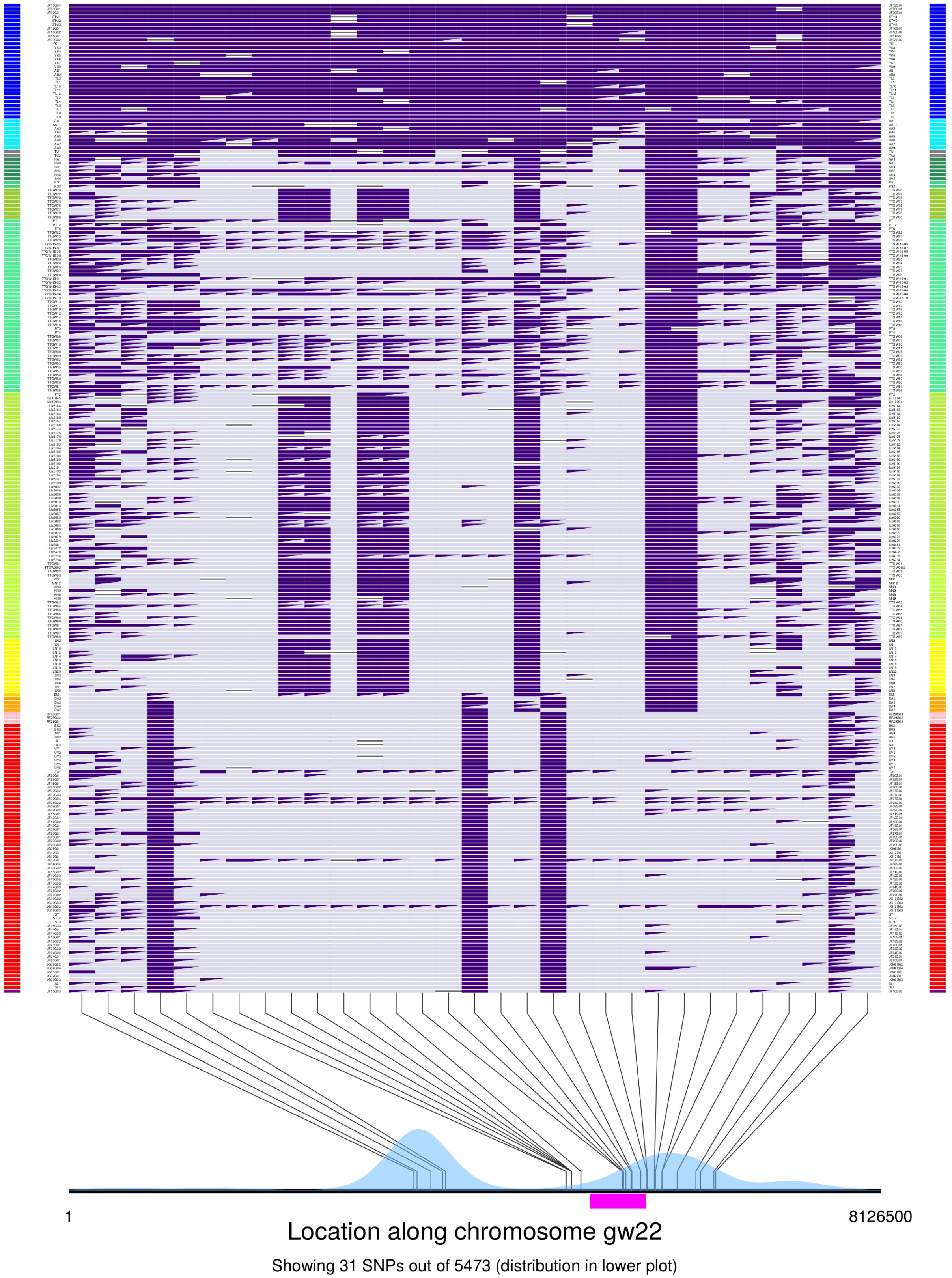

Location along chromosome gw22

Showing 31 SNPs out of 5473 (distribution in lower plot)

Figure S27:

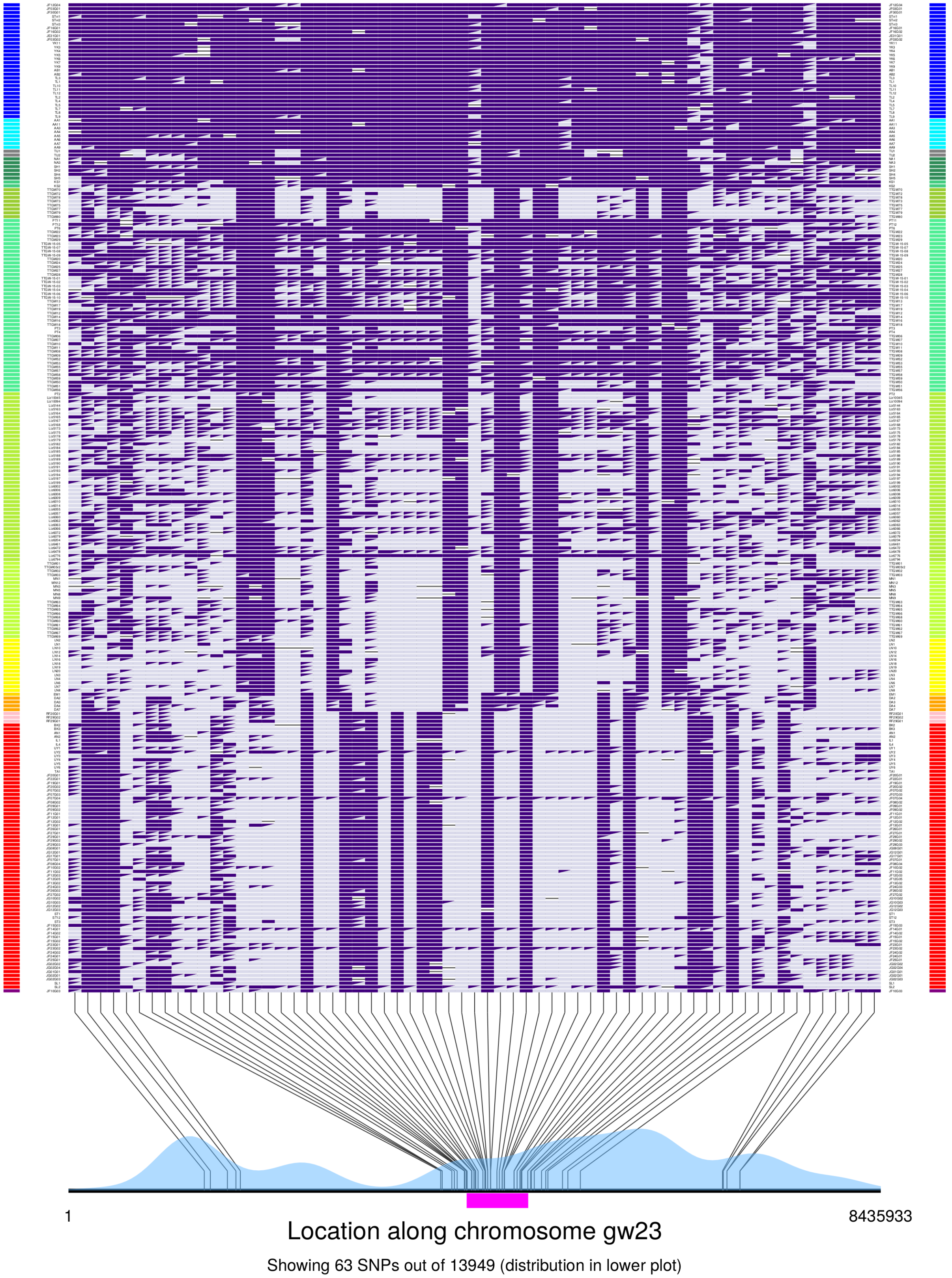
1
8435933
Location along chromosome gw23
Showing 63 SNPs out of 13949 (distribution in lower plot)

Figure S28:

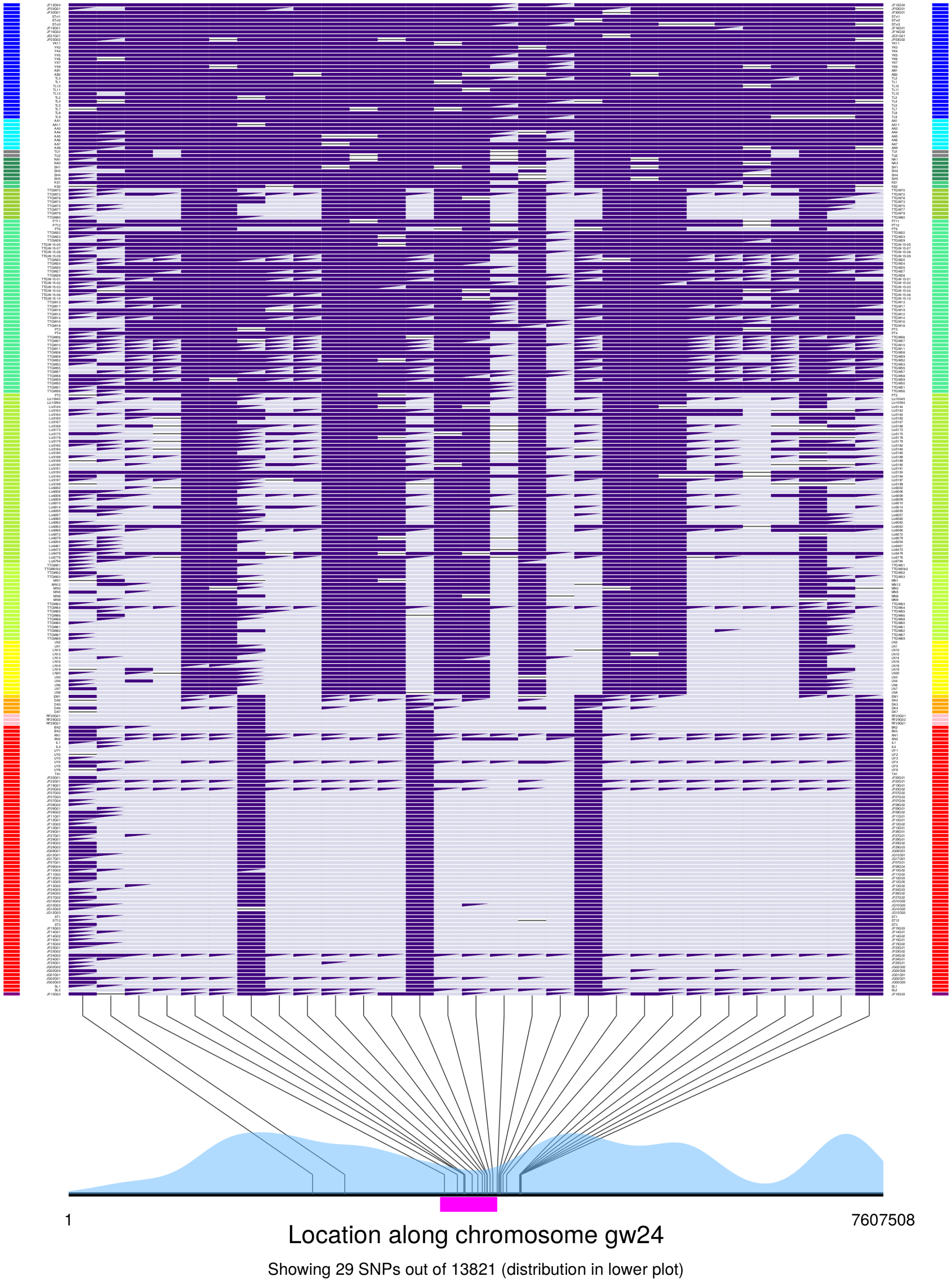
1
7607508
Location along chromosome gw24
Showing 29 SNPs out of 13821 (distribution in lower plot)

Figure S29:

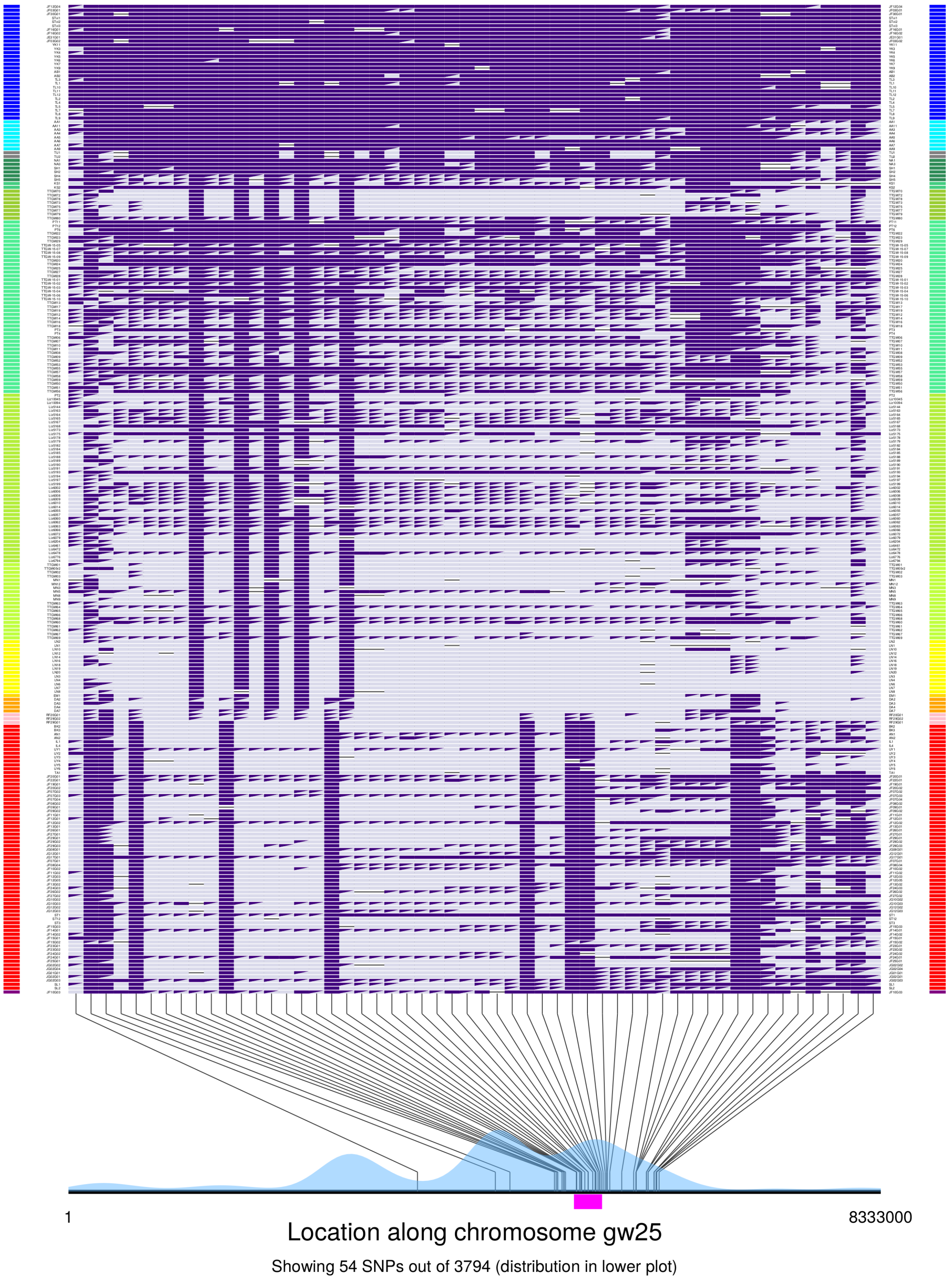
1
8333000
Location along chromosome gw25
Showing 54 SNPs out of 3794 (distribution in lower plot)

Figure S30:

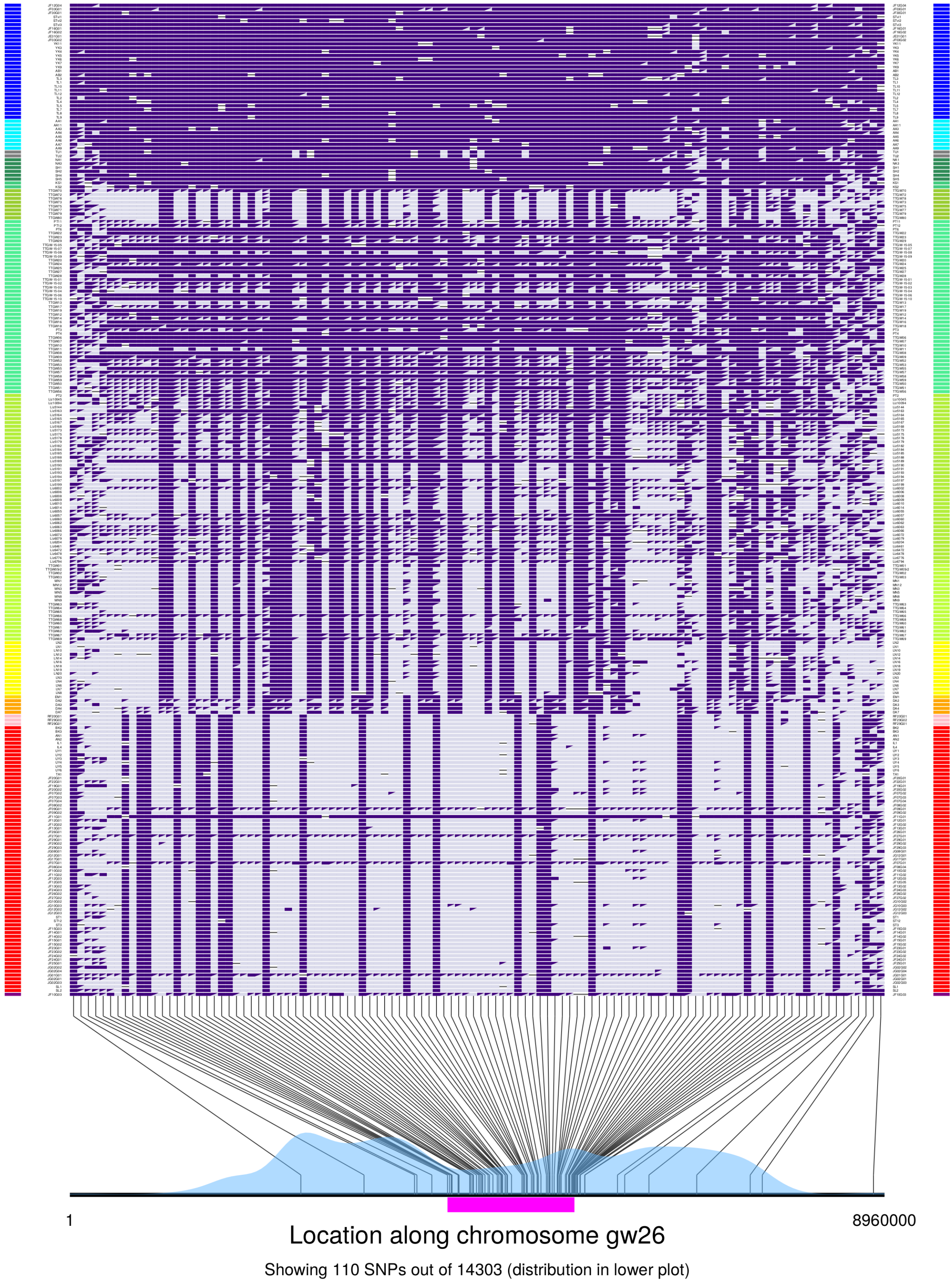

Location along chromosome gw26

Showing 110 SNPs out of 14303 (distribution in lower plot)

Figure S31:

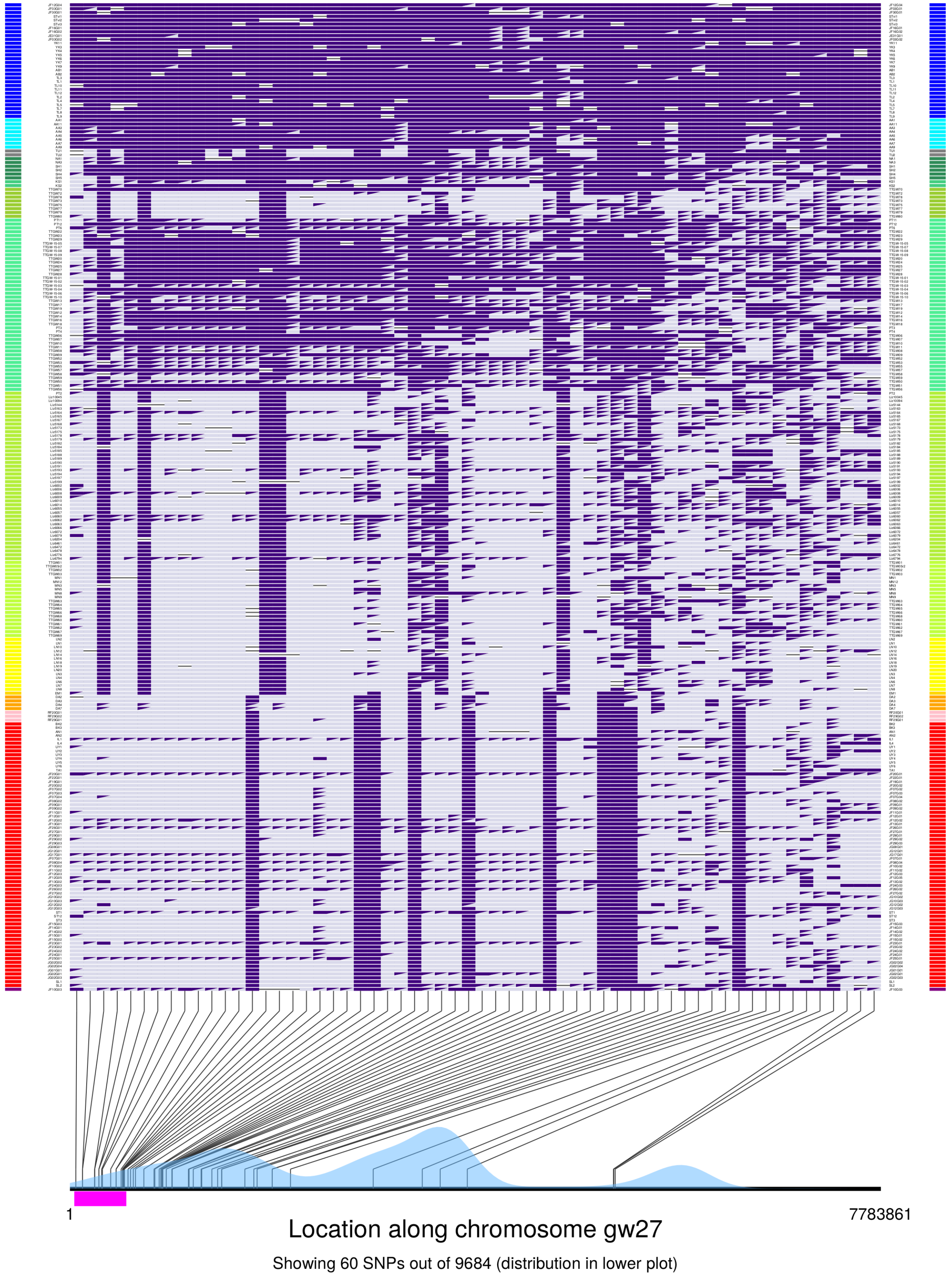
1
7783861
Location along chromosome gw27
Showing 60 SNPs out of 9684 (distribution in lower plot)

Figure S32:

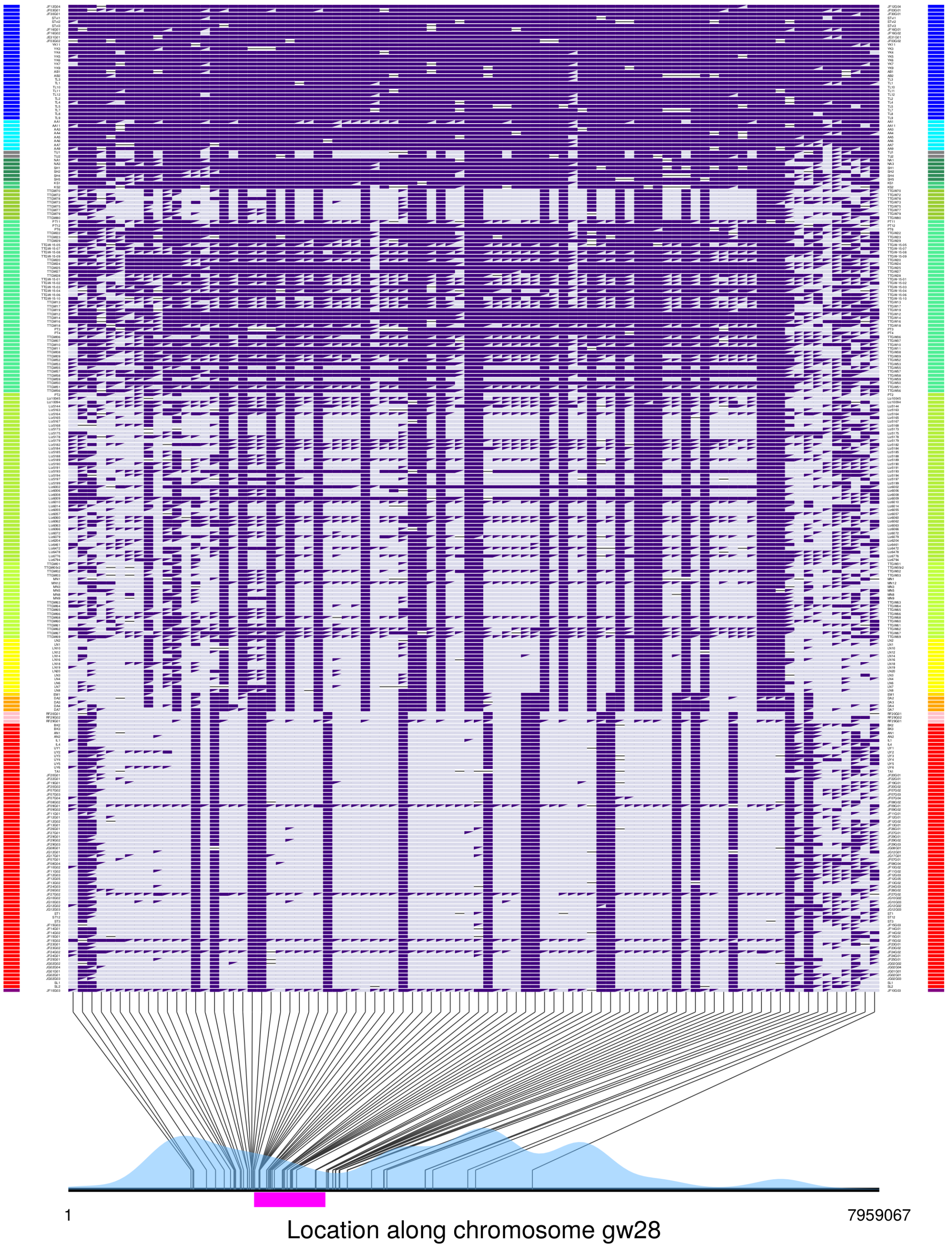

Showing 86 SNPs out of 11180 (distribution in lower plot)

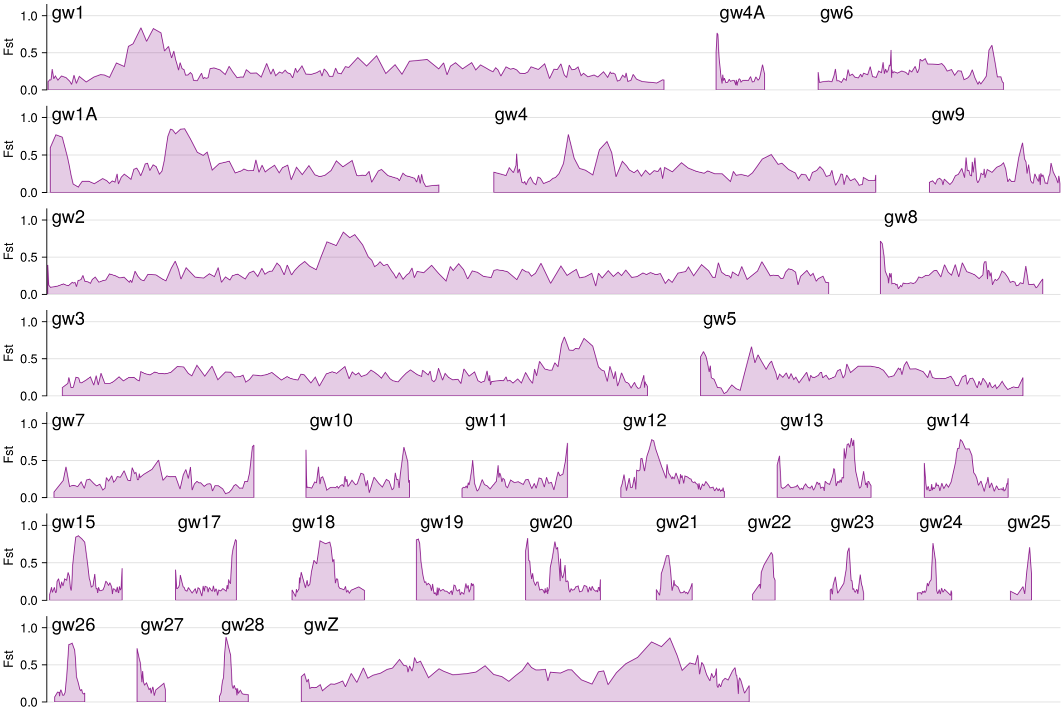

Figure S33. Windowed F_{ST} across the genome, based on a 500 SNP window size and the comparison of northern *viridanus* and *plumbeitarsus*.

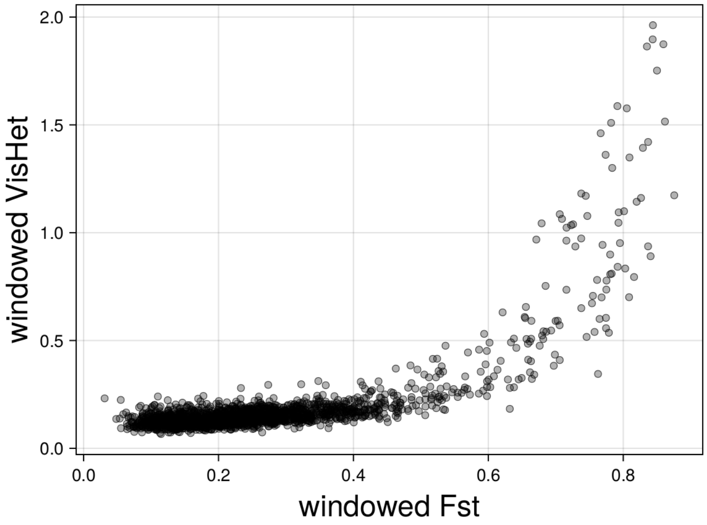

Figure S34. Windowed ViSHet and F_{ST} are strongly associated, but ViSHet distinguishes fewer windows with high values compared to F_{ST}. This figure is based on windows of 500 SNP, and genomic landscapes for each statistic can be seen in Figs. 2 and Fig. S33.
